# Stable acyclic aliphatic solid enols: synthesis, characterization, X-ray structure analysis and calculations

**DOI:** 10.1038/srep01058

**Published:** 2013-01-14

**Authors:** Yu-Qiang Zhou, Nai-Xing Wang, Yalan Xing, Yan-Jing Wang, Xiao-Wei Hong, Jia-Xiang Zhang, Dong-Dong Chen, Jing-Bo Geng, Yanfeng Dang, Zhi-Xiang Wang

**Affiliations:** 1 Technical Institute of Physics and Chemistry, Chinese Academy of Sciences, Beijing, 100190, China

## Abstract

A synthetic approach to stable enols was introduced and series of acyclic aliphatic solid enols were obtained and characterized. Relationship between the structure and the stability of these enols was discussed. Gaussian 09 calculations had been carried out to rationalize the stability of the enols. These enol structures were confirmed by ^1^H NMR, ^13^C NMR, MS, IR, partly by single crystal X-ray structure analysis and the protons exchange experiments. This work showed that very stable acyclic aliphatic enols can be synthesized efficiently without any purification.

“Simple” enols are defined as compounds with substituents, such as hydrogen, alkyl, or aryl groups, bounded to their double-bond. They are known as very unstable compounds. Enols are usually short-lived as a result of their kinetically and thermodynamically unstable properties compared to their keto forms, so they are usually present as transient intermediates at very low concentrations in various organic reactions involving aldehydes and ketones^1^,^2^,^3^. Enol compounds have been under active investigations for over one century^4^,^5^,^6^, not only from the viewpoints of synthesis^7^,^8^,^9^ and their spectroscopic behavior^10^ but also because of their roles in DNA damage^11^,^12^,^13^,^14^ and biological function^15^,^16^,^17^. However, most of the recent investigations are focused on the spectroscopy equilibrium between keto and enol formation and the factors influencing their equilibrium^18^,^19^,^20^,^21^,^22^. There are still only a few reports on the synthesis of stable enol in the literature.

Early efforts to synthesize stable enols failed because of the limited synthetic means and the misunderstanding between the stability and structure of enols. Remarkable progress in the synthesis of simple enols in early years was achieved by Capon′s^23^,^24^ and Kresge′s groups^25^,^26^. Later, scientists also made some contributions in this area^27^,^28^,^29^. However, in literature, with an equilibrium with its keto tautomers, enols are always obtained as mixtures with their carbonyl isomers. Furthermore, the existence of these enols are conditional, usually in specific solvent^30^,^31^,^32^, at low temperature^33^, in gas form^34^,^35^, or in surrounding capsule cage^36^. The concentrations of the enols are usually too low that they have to be determined indirectly. Moreover, most of these enols are stabilized by aryl^27^,^28^,^29^ or existed as cyclic structures^37^,^38^,^39^, they rapidly tautomerized to their carbonyl isomers as a result of their kinetic instability under inappropriate conditions^23^,^24^,^40^. Synthesis of stable acyclic aliphatic enols is still a challenge for chemists.

In 1922, Diels′ group reported the possibility of the existence of enol, but they were not able to confirm it^41^. They neither characterized the structure of the products of acetylacetone with DEAD nor discussed the relationship between the stability and structure of enols. After Diels′ report, in 1980, Nelson reported a Nickel-catalyzed Michael addition of *β*-carbonyl ester with azodicarboxylate, to give 1,3-dicarbonyl compounds^42^. Later, other catalysts, such as InCl_3_ and SiO_2_, were used in similar reactions to afford 1,3-dicarbonyl compounds^43^,^44^,^45^. However, no stable enol compounds were reported in these reactions. Until 2010, Lawrence described the first example of a stable phenylogous enol, resulting from an extended keto-enol tautomerization across a benzene ring. The enol has been isolated, and its structure was proved by X-ray crystallography^39^.

In this manuscript, an efficient way to synthesize very stable hydrazine substituted enol is provided and series of stable aliphatic solid enols, which are different from Lawrence′s phenylogous enol, were obtained and studied. These enols are remarkably stable under open atmosphere, and no tautomerization to their carbonyl isomers were observed. The relationship between structure and the stability of the enol was investigated. Gaussian 09 calculations had been carried out, proving that enol forms are more superior than their keto forms in energy. These enol structures are unambiguously confirmed by ^1^H NMR, ^13^C NMR, MS, IR spectra partly by single crystal X-ray structure analysis and other methods. This work showed some enol compounds can be synthesized easily without any purification. We believe this complete study on the stable enols will bring a new insight on the stability of this important species in organic synthesis.

## Results

We used ethyl acetoacetate as a standard substrate to react with DEAD in the presence of quinine and expected to get product bearing one chiral centre. After the pure product was obtained, we detected it through a chiral column on HPLC. With only one signal was detected by the chiral column, we thought a product with very high *ee* value might be obtained. When we changed the chiral base quinine into an achiral base triethylamine, we thought two signals should be observed via HPLC in the same condition. To our surprise, still only one signal was observed at the same HPLC retention time. And two broad signals at 12.11 ppm and 6.80 ppm were observed in the ^1^H NMR spectra. Then, we did rotons exchange experiments, signal at 12.11 ppm disappeared when this product was dissolved in CDCl_3_ with a drop of D_2_O, indicating the hydroxyl group is existent in the product. Besides, absorbtions at about 3280 cm^−1^ were found in IR spetrum. Based on these results, we proposed the product should be enol. This conclusion was subsequently confirmed through many ways.

**Figure 1:**
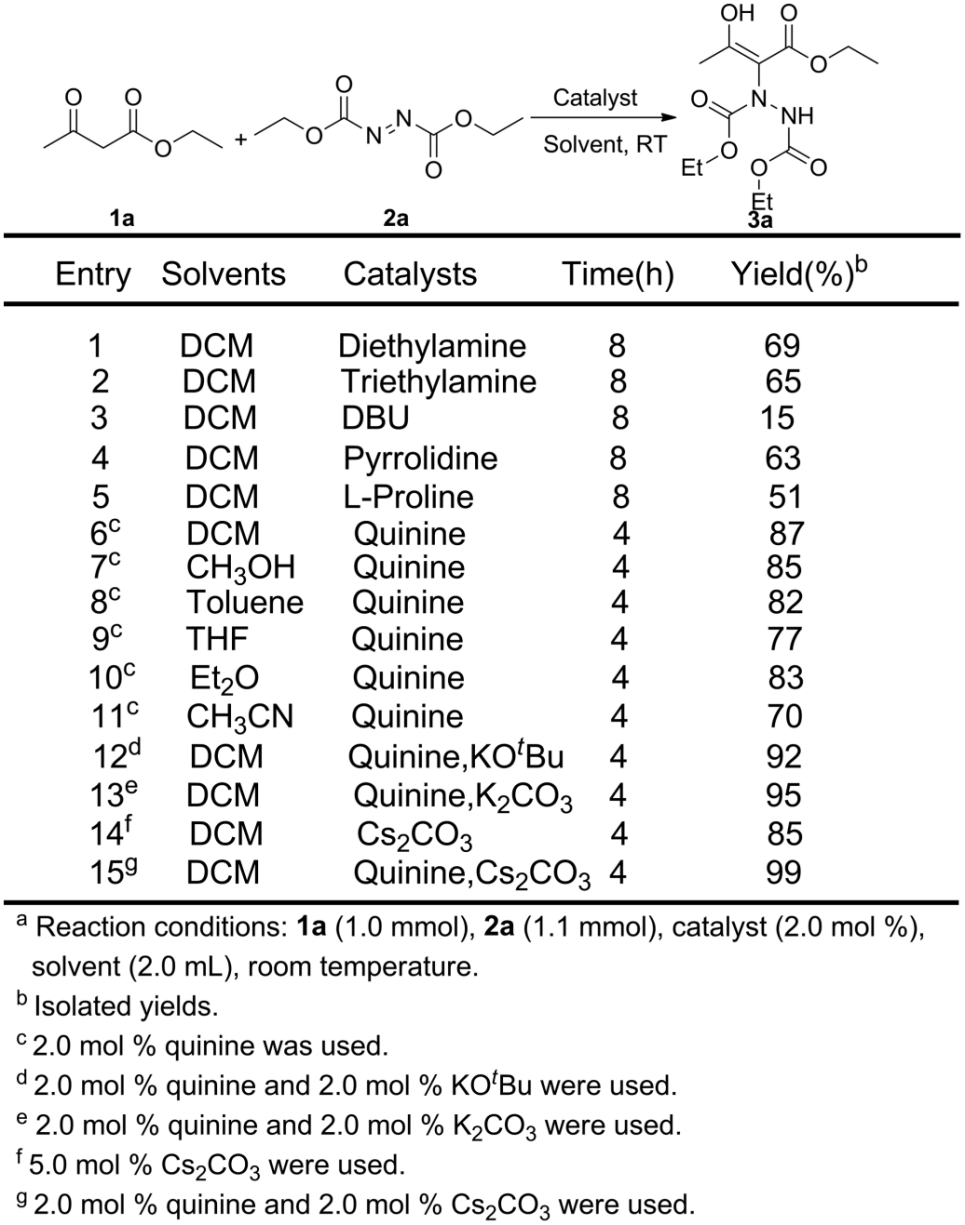
Optimized conditions for the reaction of ethyl acetoacetate 1a with DEAD 2a^a^.

As initial optimization of the reaction conditions, ethyl acetoacetate **1a** and DEAD **2a** were chosen as the model substrates. The results are summarized in Fig. 1. Different organic bases, such as diethylamine, triethylamine, DBU, pyrrolidine, L-proline and quinine were tested in dichloromethane (DCM) at room temperature (entries 1–6). It was confirmed that all of the organic bases mentioned can be used as catalysts in this reaction. However, quinine seems to be the best one, because it can offer both the fastest rate and excellent yields with lower catalyst loading. The solvents were subsequently examined, we found DCM is a suitable one among various solvents listed in Fig. 1 (entries 7–11). To our delight, the best result (99%) was achieved when quinine and Cs_2_CO_3_ were employed at the same time in this reaction (entries 15), compared with using KO*t*Bu, K_2_CO_3_ (entries 12, 13) or only use of Cs_2_CO_3_(entries 14).

**Figure 2:**
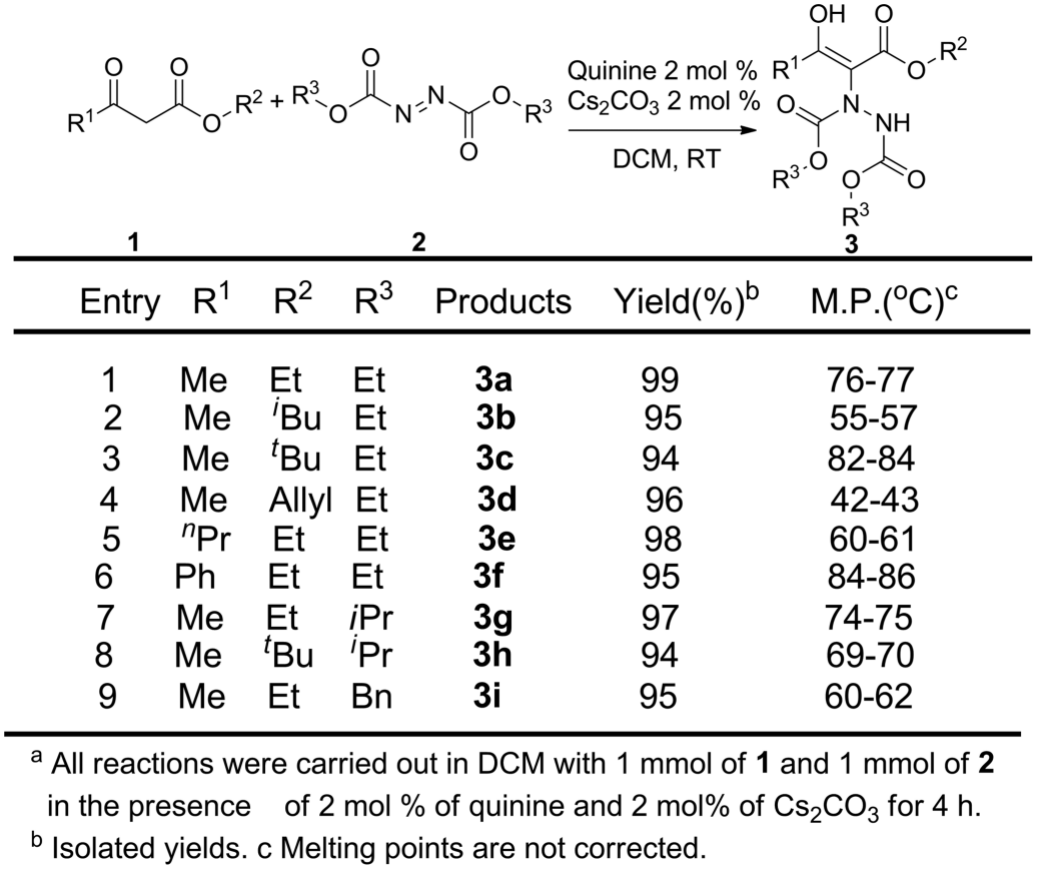
Expansion of *β*-carbonyl esters 1 with azodicarboxylates 2^a^.

With the optimized conditions in hand, we then investigated the scope of substitutents on this reaction. Various *β*-carbonyl esters and azodicarboxylates were examined, as summarized in Fig. 2. We found that these reactions can be carried out without limitation in the groups R^1^ or R^2^, because these carbonyl esters readily participated to react with DEAD. However, yields are a little bit lower when substitutents are bulky groups such as *^t^*Bu (94%) (entry 3). Even **3f** with Bn group in its structure, 95% yield was achieved (entry 6). All of the reactions performed smoothly when carbonyl esters reacted with several azodicarboxylates (entries 1, 7–9). Although **3g**, **3h**, **3i** were synthesized from substrates with bulky groups, corresponding products were obtained in excellent yields (94–97%)(entries 7–9). In all of cases, the products were obtained with excellent yields, we believe that the groups R^1^, R^2^, R^3^ have limited impact on the yields.

**Figure 3:**
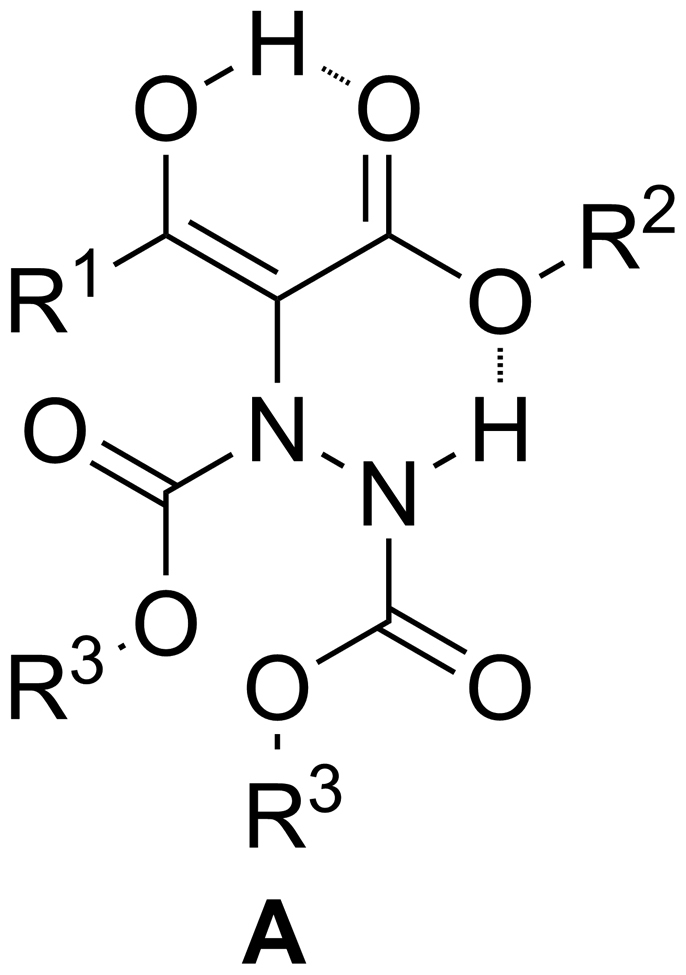
Possible intramolecular hydrogen bonds in A.

With our efforts to understand this reaction, we became interested to know why these enol form products are stable. We decided to study products **A** obtained from *β-*carbonyl esters. We found intramolecular hydrogen bonding is very important to the stability of enols. Initially, we thought **A** could be stabilized by one O–H–O intramolecular hydrogen bond and one N–H–O intramolecular hydrogen bond (Fig. 3).

**Figure 4:**
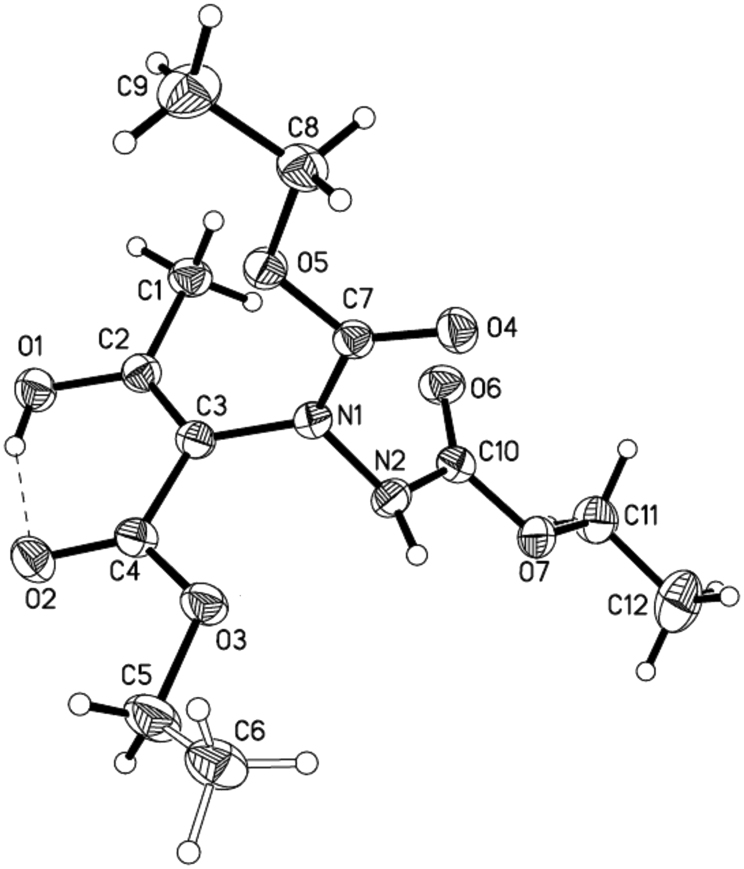
The X-ray crystal structure of compound 3a. Intramolecular hydrogen bond is displayed in dashed lines.

We speculated that both of these two intramolecular hydrogen bonds were working together to maintain the stability of these enols and disenable the isomerization to their keto isomers. We tried to prove our hypothesis by the single crystal X-ray structure analysis (Fig. 4). From the data of single crystal X-ray structure analysis of compound **3a** (CCDC 816568 contains the supplementary crystallographic data for this compound. These data can be obtained free of charge from The Cambridge Crystallographic Data Centre via www.ccdc.cam.ac.uk/data_request/cif), we could see O^1^H group and O^2^, with a distance of 1.789 Å are close enough to form intramolecular hydrogen bonds. However, the distance of N^2^H group with O^3^ is beyond of the range of intramolecular hydrogen bonds. Therefore, O–H–O intramolecular hydrogen bond plays very important role to the enol and it is strong enough to maintain the enol form. This speculation was subsequently proved by performing deuterated solvent study by ^1^H NMR. The examination of **3a** in CDCl_3_, CD_3_OD, C_6_D_6_, CD_3_COCD_3_, CD_3_CN and CD_3_SOCD_3_ showed that enol form products can be well maintained in aprotic solvent, such as CDCl_3_, C_6_D_6_ and CD_3_CN. However, some protic solvents and protophilic solvents affected the intramolecular hydrogen bonds in **3a**, because second small signals in the enol proton region at 12.0 ppm were found, such as in solvents CD_3_COCD_3 _and CD_3_SOCD_3. _These additional signals should belong to enol protons which are partly released from previous intramolecular hydrogen bond as a result of the disruption of carbonyl or sulfinyl. It is worth mentioning that when CD_3_COCD_3 _or CD_3_SOCD_3_ was moved and CDCl_3 _was added, its ^1^H NMR data is as same as the data of **3a** which was directly collected in CDCl_3_. Although protophilic solvent, such as CD_3_COCD_3 _and CD_3_SOCD_3_, can partly disrupt the intramolecular hydrogen bonds in these enols, enol forms remain predominant from their ^1^H NMR data. Moreover, we are pretty confident that the products are enols since enol signal at 12.11 ppm disappeared when this product was dissolved in CD_3_OD (proton exchanged).

**Figure 5:**
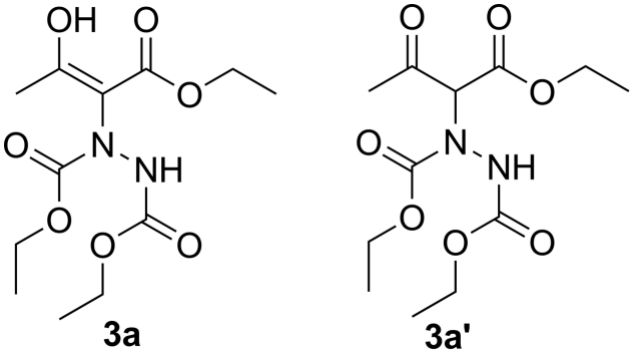
The model structures of *β*-carbonyl ester 3a and its keto form 3a′.

**Figure 6:**
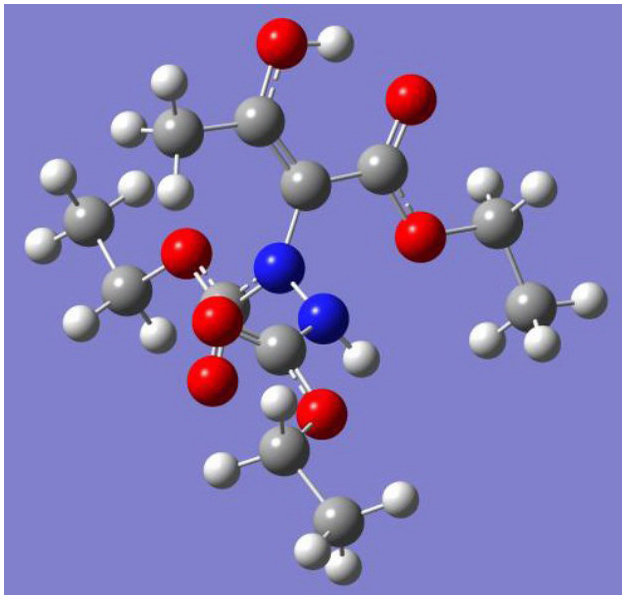
The optimized structure of 3a.

Computationally study about enol is one topic attracted many chemists^46^,^47^,^48^,^49^,^50^. Energy calculated by the computer is another important factor taken into consideration by our group. Take product **3a** for example, as a result of the effect of the intramolecular hydrogen bond, the carbon-carbon double bond is formed (Fig. 5, **3a**). With oxygen atom, nitrogen atom and carbonyl bonded to the carbon-carbon double bond in **3a**, p-π conjugation and π-π conjugation are possible, which strongly dispersed the electrons. When **3a** tautomerized to **3a′**, however, p-π conjugation and π-π conjugation are not possible because of the barrier of the tertiary carbon. As a result of this, we supposed **3a** should have lower energy in theory, which benefits the enol form and stabilizes the enol forms. This hypothesis was proved by the Gaussian 09 calculations in this paper (See supporting information). We chose **3a** and its keto form isomer **3a′** as the model structure to perform the Gaussian 09 calculations (Fig. 5). The optimized structure is quite in accord with the single crystal X-ray structure (Fig. 6). The results showed that energies of **3a** are lower than **3a′**(H**_3a_** − H**_3a′_** = −9.6 Kcal/mol, G**_3a_** −G**_3a′_** = −8.8 Kcal/mol in gas form, H**_3a_** −H**_3a′_** = −6.9 Kcal/mol, G**_3a_** −G**_3a′_** = −6.2 Kcal/mol in the solvent model of CH_2_Cl_2_ ) (See supporting information), supporting that the superiority of the enol form of **3a** is obvious when it is compared with its keto form isomer **3a′**.

**Figure 7:**
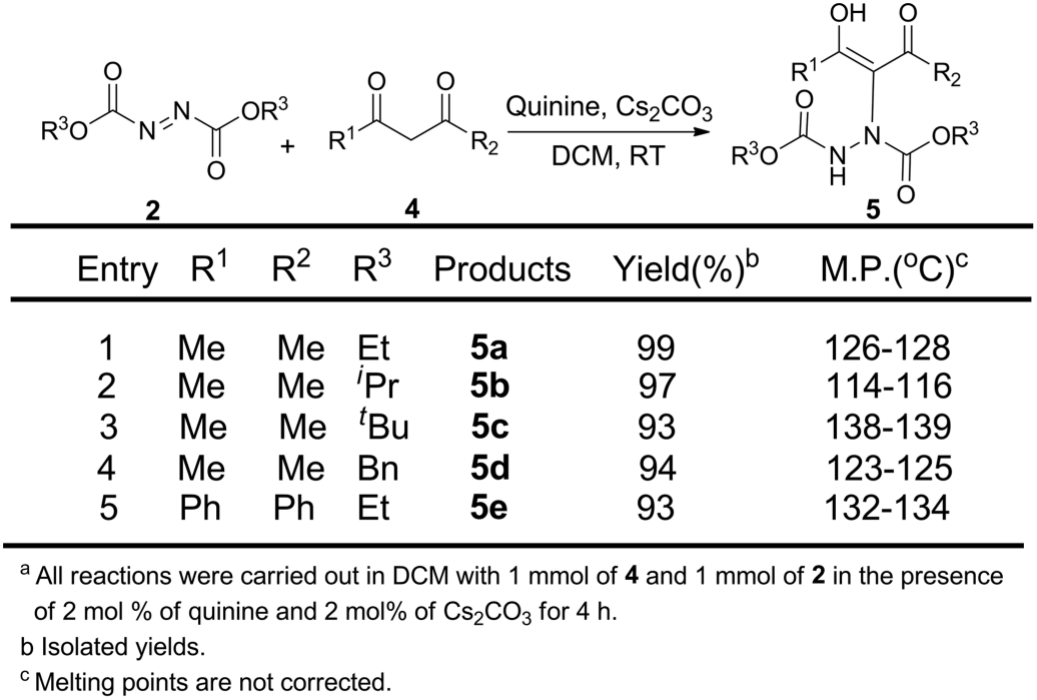
Expansion of *β*-carbonyls 4 with azodicarboxylates 2^a^.

To confirm that the intramolecular hydrogen bond is strong enough to maintain the enol form, we expanded the substrate from *β*-carbonyl esters to *β*-carbonyl ketone compounds, some enol form products were obtained (Fig. 7). It was pleasant that all the reactions performed very well by carrying out the foregoing condition and all substrates provided high yield products (entries 1–5). Such as, products **5a** and **5b** were obtained with 99% and 97% yields (entries 1, 2). However, substrates with bulky groups, such as, *^t^*Bu, Ph slightly lowered the yields (entries 3–5). It should be mentioned that the size of the central substituent has slightl effect on the formation of the intramolecular hydrogen bonds because additional signals in the enol proton region at 16.0 ppm were found in the spectra of **5b** and **5c** and the second signal in ^1^H NMR spectra of **5c** with groups *^t^*Bu is stronger than the second signal in ^1^H NMR spectra of **5b** with groups *^i^*Pr. Steric hindrance should lead to a distortion and disrupted intramolecular hydrogen bonds so that the enol proton can partly release from intramolecular hydrogen bond to give an additional signal. For the same reason, additional signals could be found in the ^13^C NMR spectrum of **5b** and **5c.** However, all the products were detected through the chiral column on HPLC, each of them gave one signal, which means the enol form was obtained with overwhelming superiority by the mobile phase of *n*-Hexane and 2-propanol.

Thus, product **5a** was easily obtained as the only product in enol form. Because only one signal was determined through the chiral column on HPLC and signal at 16.02 ppm disappeared while deuterated with D_2_O in CDCl_3_, it indicates that the product **5a** is also an enol form with overwhelming superiority.

**Figure 8:**
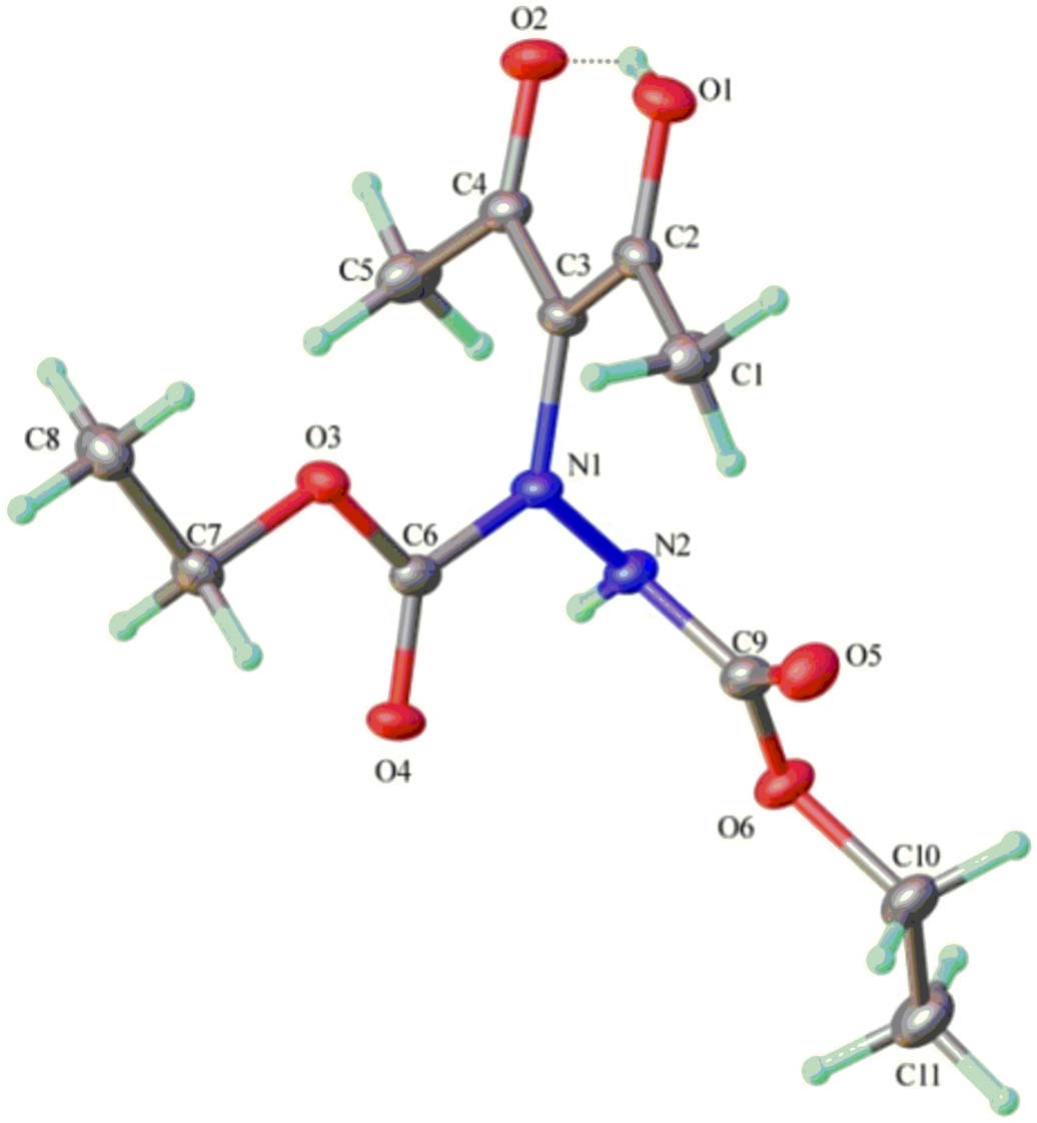
The X-ray crystal structure of compound 5a. Intramolecular hydrogen bond is displayed in dashed lines.

Similarly, **5a** also have lower energy which benefits the enol form and stabilizes its enol structure. Luckily, this was also proved by the Gaussian 09 calculations when we chose **5a** and its keto form isomer **5a′** as the model structure to perform the calculations. The optimized structure is quite similar with the single crystal X-ray structure (See supporting information). The results showed that the energy of **5a** is lower than **5a′** (H**_5a_** − H**_5a′_** = −9.4 Kcal/mol, G**_3a_** − G**_3a′_** = −9.5 Kcal/mol in gas form, H**_5a_** − H**_5a′_** = −6.5 Kcal/mol, G**_5a_** −G**_5a′_** = −6.7 Kcal/mol in the solvent model of CH_2_Cl_2_), indicating enol forms are more superior than their keto forms in energy. Enol form structure of product **5a** was unambiguously confirmed by the single crystal X-ray structure analysis at last (Fig. 8). (CCDC 849478 contains the supplementary crystallographic data for this compound. These data can be obtained free of charge from The Cambridge Crystallographic Data Centre via www.ccdc.cam.ac.uk/data_request/cif). From the data of single crystal X-ray of **5a**, we can find proton H1 is between O1 and O2, the distance of H1 with O1 and the distance of H1 with O2 are close enough to form intramolecular hydrogen bonds. However, the acetoacetate fragment is not completely symmetrical because the distance of H1- O1 and the distance of H1-O2 are not equivalent. And the distance of O1-C2 and O2-C4 are not equivalent either. With a 72.4 deg. angle between C4-C3 and N1-N2, it seems that the hydrazine moiety is not orthogonal with the acetoacetate fragment. But hydrazine with bulky groups should have impact on the structure because it can lead to a deformation. Indeed, large hydrazine substituents could also likely favor the formation of the enol relative to the keto isomer.

In conclusion, three main reasons should be responsible for the stability of enol form. 1) the intramolecular hydrogen bond of O–H–O, 2) the p-π conjugation and π-π conjugation in these compounds. 3) appropriate group is another factor to the enol.

**Figure 9:**
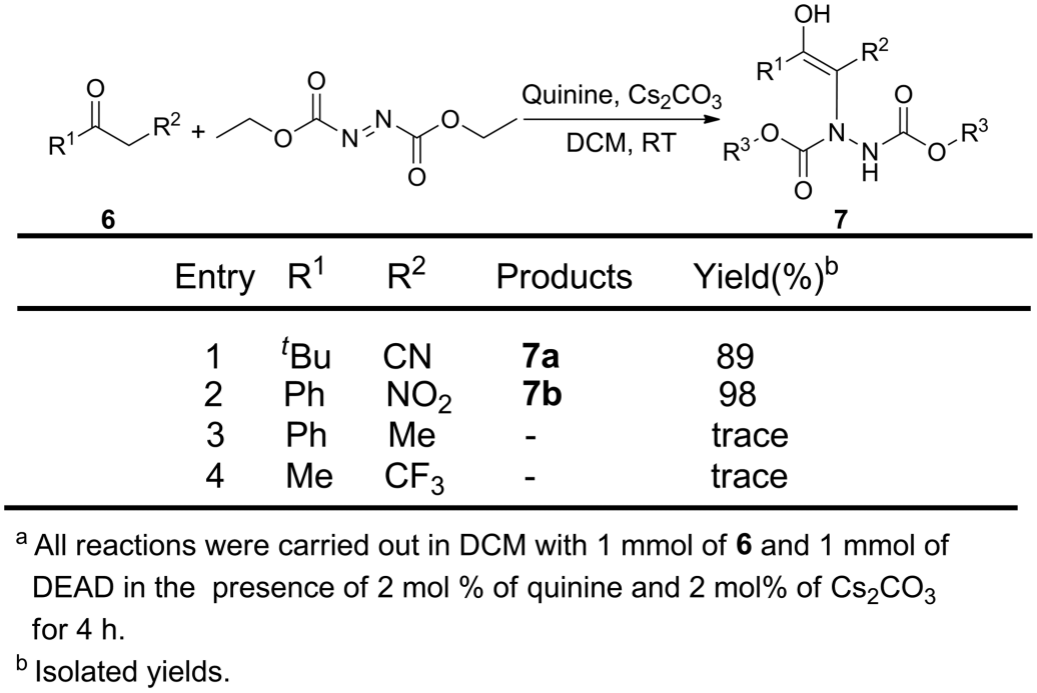
Expansion of carbonyls 6 with DEAD^a^.

With our efforts to understand the relation between the structure and the stability of enols, we expanded substrates with groups at the beta position to more common groups, such –NO_2_,–CN, Me and CF_3_ to react with DEAD (Fig. 9). Substrates with electron withdrawing group, such as, CN and NO_2_ readily performed with DEAD in the present of quinine, giving **7a** and **7b** with good yields (entries 1 and 2). Each one of **7a** and **7b** showed one signal through the chiral column on HPLC, which means two enol form compounds were yielded. However, when we used propiophenone as a substrate to react with DEAD in the presence of quinine and Cs_2_CO_3_, only trace amount of product was determined after 24 h (entry 3). This is probably because the reaction can't be carried out with this condition^51^. For the same reason entry 4 can't work either.

## Discussion

As a conclusion, an efficient method to prepare stable enols was introduced and series of acyclic aliphatic solid enols were synthesized. The relationship between structure and the stability of enols was discussed. We found that groups beta to the carbonyl in substrates are important to formation of enols, because these groups could either balance the electron by the p-π conjugation and π-π conjugation or could form intramolecular hydrogen bond with OH. With our method, stable acyclic aliphatic solid enols were easily obtained as only products. Notably, all of the enols synthesized can be stably maintained in normal condition. Gaussian 09 calculations had been carried out by using enols mentioned in this paper and their keto isomers as models structures, proving that enol forms are more predominant than their keto forms in energy. These enol structures are confirmed by ^1^H NMR, ^13^C NMR, MS, partly by single crystal X-ray structure analysis and the protons exchange experiments. We believe this is a complete study on an important species in organic synthesis. Further studies about other types of stable enols are currently under investigation and will be presented in a due time.

## Methods

All calculations were carried out with the Gaussian 09 programs. The geometrical optimizations of all the complexes were performed using M05-2X with the 6–31G** basis set for all atoms. Frequency calculations at the same level were performed to confirm each stationary point to be a minimum. The free energies of solvation in this study were calculated based on the gas phase optimized structures with the polarizable continuum model (PCM) using UA0 radii. The dielectric constant in the PCM calculations was set to 8.93 to simulate dichloromethane (CH_2_Cl_2_), the solvent medium in the experiments. The single point energies were also computed using the M05-2X method with the 6–311++G** basis set for all atoms. The report free energies and enthalpies include zero-point energies and thermal corrections calculated at 298.15K and 1 atm.

Unless otherwise noted, materials were used as commercial suppliers. All solvents were purified by standard method. Flash column chromatography was performed using 200–300 mesh silica gel.

Reaction progress was followed by TLC analysis at 254 nm. NMR spectroscopy was performed on 400 MHz spectrometer operating at 400 MHz (^1^H NMR) and 100 MHz (^13^C NMR). TMS was used as an internal standard and CDCl_3_ was used as the solvent ^1^H NMR data were reported as follows: chemical shifts in ppm downfield from tetramethylsilane, multiplicity (s = singlet, d = doublet, t = triplet, q = quartet, m = multiplet and br = broad), *J* = coupling constant. IR spectra were recorded by using KBr optics. All the reagents are used directly from commercial and without further purification.

### General procedure for the synthesis of all enols

To a solution of carbonyl compounds (1.0 mmol) in CH_2_Cl_2_, azodicarboxylates (1.0 mmol ) was added. And followed by the addition of quinine (0.02 mmol) and Cs_2_CO_3 _(0.02 mmol). The mixture was stirred for 4–8 h at room temperature. The reaction was monitored by TLC (ethyl acetate : petroleum ether = 1:5 V/V). After evaporation of the solvents, the residue was purified by silica gel column chromatography (ethyl acetate : petroleum ether = 1:5 V/V). Full experimental details and the characterization data for all the compounds are given in the Supplementary Information.

## Author Contributions

Yu-Qiang Zhou and Nai-Xing Wang wrote the main manuscript text and Yanfeng Dang and Zhi-Xiang Wang prepared figure 6. All authors reviewed the manuscript.

## Supplementary Material

Supplementary Information Stable acyclic aliphatic solid enols: synthesis, characterization, X-ray structure analysis and calculations
