# Supplementary material for: Stable acyclic aliphatic solid enols: synthesis, characterization, X-ray structure analysis and calculations
Source: Sci Rep. 2013 Jan 14;3:1058. doi: 10.1038/srep01058 (PMC3544012; doi:10.1038/srep01058)
Supplement: Supplementary Information — Stable acyclic aliphatic solid enols: synthesis, characterization, X-ray structure analysis and calculations [file srep01058-s1.doc]

**Supporting Information**

**Stable Acyclic Aliphatic Solid Enols: Synthesis, Characterization, X-Ray Structure Analysis and Calculations**

Yu-Qiang Zhou, Nai-Xing Wang,**^a^* Yalan Xing, Yan-Jing Wang, Xiao-Wei Hong, Jia-Xiang Zhang, Dong-Dong Chen, Jing-Bo Geng, Yanfeng Dang, Zhi-Xiang Wang

*^a^* *Technical Institute of Physics and Chemistry, Chinese Academy of Sciences, Beijing, 100190, China. Fax +86(10)62554670; Tel: +86(10)82543575;*

E-mail: nxwang@mail.ipc.ac.cn

Table of Contents:

**1**. General……………………………………………………………….…............................…................S3

**2**. General Procedure for **3a**……………………………..………..........................….……………............S3

**3**. Characterization Data for **3a-3i**, **5a-5e**, **7a**,**7b**…………...……………..…………….……...........S4-S10

**4**. ^1^H Spectra for **3a-3i, 5a-5e, 7a,7b**……………..……………………………………...................S11-S33

**5**. ^13^C NMR Spectra for **3a-3i, 5a-5e, 7a,7b**……………..…………………………………............S34-S49

**6**. IR Spectra for **3a, 3B, 5a,5b, 7a**…………......………………………………….….....................S50-S52

**7**. MS Spectra for **3a-3g, 5a-5e**…………………………………………………….….....................S53-S60

**8**. HPLC Spectra for **3a**-**3i, 5a-5e, 7a,7b**…………………………………......................….............S61-S81

**9**. Single crystal X-ray structure of **3a** …………………………….....................…….….…...........S82-S86

**10.** Single crystal X-ray structure of **5a** ………………………….....................…….….….............S87-S91

**11.** Caculation of **3a** and its keto form isomer **3a’**…………………....................……...……..........S92-S95

**12**. Caculation of **5a** and its keto form isomer **5a’**…………………........................…….……........S95-S98

1. General

Unless otherwise noted, materials were used as commercial suppliers. All solvents were purified by standard method. Flash column chromatography was performed using 200-300 mesh silica gel. Reaction progress was followed by TLC analysis at 254 nm. NMR spectroscopy was performed on 400 MHz spectrometer operating at 400 MHz (^1^H NMR) and 100 MHz (^13^C NMR). TMS was used as an internal standard and CDCl_3_ was used as the solvent. ^1^H NMR data were reported as follows: chemical shifts in ppm downfield from tetramethylsilane, multiplicity (s = singlet, d = doublet, t = triplet, q = quartet, m = multiplet and br = broad), coupling constant = *J*. IR spectra were recorded by using KBr optics. All the reagents are used directly from commercial and without further purification.

1. General Procedure for **3a**

To a solution of ethyl acetoacetate **1a** (1.0 mmol) in CH_2_Cl_2_, DEAD **2a** (1.0 mmol ) was added. And followed by the addition of quinine (0.02 mmol) and Cs_2_CO_3_ (0.02 mmol). The mixture was stirred for 4-8 h at room temperature. The reaction was monitored by TLC (ethyl acetate : petroleum ether = 1:5 V/V). After evaporation of the solvents, the residue was purified by silica gel column chromatography (ethyl acetate : petroleum ether = 1:5 V/V).

1. Characterization Data for **3a-3i, 5a-5e, 7a,7b**

(*E*)-diethyl 1-(1-ethoxy-3-hydroxy-1-oxobut-2-en-2-yl)hydrazine-1,2-dicarboxylate (**3a**):


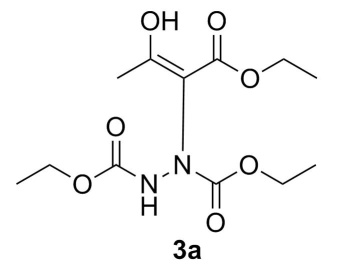


**(*E*)-diethyl 1-(1-ethoxy-3-hydroxy-1-oxobut-2-en-2-yl)hydrazine-1,2- dicarboxylate (3a)**: White powder, 99 % yield; M.p. 76-77 ^o^C;. **^1^H NMR** (400 MHz, CDCl_3_, 25°C, TMS) *δ*=1.33-1.21 (m, 9H; 3CH_2_C*H*_3_), 2.27 (s, 3H; C*H*_3_), 4.28-4.16 (m, 6H; 3C*H*_2_CH_3_ ), 6.80 (br, 1H; N*H*), 12.11 (br , 1H; O*H*). **^13^C NMR** (100 MHz, CDCl_3_, 25°C, TMS) *δ*=14.29 (CH_2_*C*H_3_), 14.40 (2C, 2CH_2_*C*H_3_ ), 18.09 (*C*H_3_), 61.22 (*C*H_2_CH_3_), 61.92 (*C*H_2_CH_3_), 63.10 (*C*H_2_CH_3_), 107.38 (*C*=COH), 156.10 (*C*ONH), 156.44(*C*ON), 169.71 (*C*OOCH_2_CH_3_), 177.23 (*C*OH). **IR** (KBr) 3295, 2987, 1752, 1712 1655,1627, 1243, 1071 cm^-1^.**HRMS** (EI+): *m/z* calcd for C_12_H_20_N_2_O_7_, 304.1271. Found 304.1264.

(*E*)-diethyl1-(3-hydroxy-1-isobutoxy-1-oxobut-2-en-2-yl)hydrazine-1,2-dicarboxylate(**3b**):


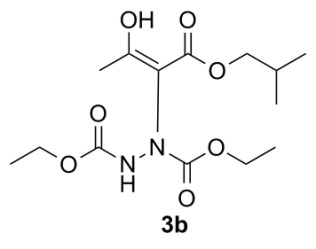


**(*E*)-diethyl 1-(3-hydroxy-1-isobutoxy-1-oxobut-2-en-2-yl)hydrazine-1,2-dicarboxylate(3b):** White powder, 95 % yield; M.p. 55-57 ^o^C; **^1^H NMR** (400 MHz, CDCl_3_, 25°C, TMS) *δ*=0.91 (m, 6H; CH(C*H*_3_)_2_), 1.27-1.25 (m, 6H), 1.97-1.95 (m, 1H; C*H*), 2.27 (s, 3H; C*H*_3_), 4.04-3.91 (d, *J* = 2.8Hz, 2H; C*H*_2_), 4.19 (m, 4H; 2C*H*_2_CH_3_ ), 6.73 (br, 1H; N*H*), 12.09 (br, 1H; O*H*). **^13^C NMR** (100 MHz, CDCl_3_, 25°C, TMS) *δ*=15.19 (CH_2_*C*H_3_), 15.25 (CH_2_*C*H_3_), 18.86 (*C*H_3_), 19.68 (2C, CH(*C*H_3_)_2_), 28.50 (*C*H(CH_3_)_2_), 62.74 (*C*H_2_CH_3_), 63.93 (*C*H_2_CH_3_), 71.77 (*C*H_2_CH(CH_3_)_2_), 108.10 (*C*=COH), 156.84 (*C*ONH), 157.24 (*C*ON), 170.48 (*C*OOCH_2_CH_3_), 178.29 (*C*OH). **IR** (KBr) 3306, 2989, 2916, 1749, 1708, 1651, 1249, 1066, 857. cm^-1^ .**HRMS** (EI+): *m/z* calcd for C_14_H_24_N_2_O_7_, 332.1584. Found 332.1585.

(*E*)-diethyl 1-(1-(tert-butoxy)-3-hydroxy-1-oxobut-2-en-2-yl)hydrazine-1,2-dicarboxylate(**3c**):


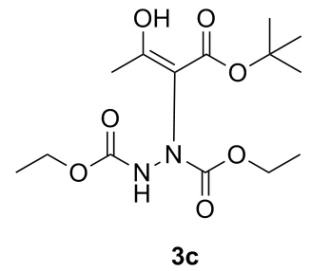


**(*E*)-diethyl 1-(1-(tert-butoxy)-3-hydroxy-1-oxobut-2-en-2-yl)hydrazine-1,2-dicarboxylate(3c):** White powder, 94 % yield; M.p. 82-84 ^o^C. **^1^H NMR** (400 MHz, CDCl_3_, 25°C, TMS) *δ*=1.30-1.27 (m, 6H; 2CH_2_C*H*_3_), 1.48 (m, 9H; C(C*H*_3_)_3_), 2.26 (s, 3H; C*H*_3_), 4.21-4.18 ( m, 4H; 2C*H*_2_CH_3_ ), 6.64 (br, 1H; ; N*H*), 12.25 (br , 1H; O*H*). **^13^C NMR** (100 MHz, CDCl_3_, 25°C, TMS) *δ*=14.54 (CH_2_*C*H_3_), 14.71 (CH_2_*C*H_3_), 18.20 (*C*H_3_), 28.44 (3C, C(*C*H_3_)_3_), 62.09 (*C*H_2_CH_3_), 63.15 (*C*H_2_CH_3_), 83.00 (*C*(CH_3_)_3_), 108.42 (*C*=COH), 156.23 (*C*ONH), 156.87 (*C*ON), 169.59 (*C*OOCH_2_CH_3_), 177.24 (*C*OH). **IR** (KBr) 3284, 2986, 1750, 1707, 1641, 1514, 1241, 1162 1064 cm^-1^. **HRMS** (EI+): *m/z* calcd for C_14_H_24_N_2_O_7_, 332.1584. Found 332.1585.

(*E*)-diethyl 1-(1-(allyloxy)-3-hydroxy-1-oxobut-2-en-2-yl)hydrazine-1,2-dicarboxylat(**3d**):


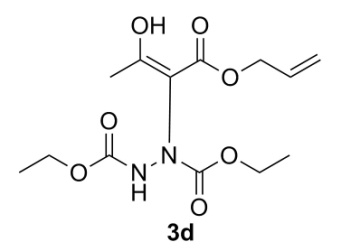


**(*E*)-diethyl 1-(1-(allyloxy)-3-hydroxy-1-oxobut-2-en-2-yl)hydrazine-1,2-dicarboxylat (3d):** White powder, 96 % yield; M.p. 42-43 ^o^C. **^1^H NMR** (400 MHz, CDCl_3_, 25°C, TMS) *δ*=1.29-1.22 (m, 6H; 2CH_2_C*H*_3_), 2.39-2.28 (m, 3H; C*H*_3_), 4.20-4.16 (m, 4H; 2C*H*_2_CH_3_), 4.73-4.66 (m, 2H; C*H*_2_CH=CH_2_), 5.31-5.22 (m, 2H; CH_2_CH=C*H*_2_), 5.90-5.85 (m, 1H; CH_2_C*H*=CH_2_ ), 6.82 (br, 1H; N*H*), 12.02 (br , 1H ; O*H*). **^13^C NMR** (100 MHz, CDCl_3_, 25°C, TMS) *δ*=14.49 (2C, CH_2_*C*H_3_), 18.24 (*C*H_3_), 62.06 (*C*H_2_CH_3_), 63.25(*C*H_2_CH_3_), 65.59 (*C*H_2_CH=CH_2_), 107.30 (*C*=COH), 118.83 (CH_2_CH=*C*H_2_), 131.51 (CH_2_*C*H=CH_2_), 156.16 (*C*ONH), 156.46 (*C*ON), 169.40 (*C*OOCH_2_CH_3_), 177.93 (*C*OH). **IR** (KBr) 3283, 2993, 1749, 1709, 1658, 1514, 1319, 1239, 1064. **HRMS** (EI+): *m/z* calcd for C_13_H_20_N_2_O_7_, 316.1271. Found 316.1282.

(*E*)-diethyl 1-(1-ethoxy-3-hydroxy-1-oxohex-2-en-2-yl)hydrazine-1,2-dicarboxylate(**3e**):


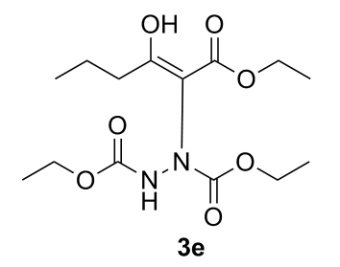


**(*E*)-diethyl 1-(1-ethoxy-3-hydroxy-1-oxohex-2-en-2-yl)hydrazine-1,2-dicarboxylate (3e):** White powder, 98 % yield; M.p. 60-61 ^o^C. **^1^H NMR** (400 MHz, CDCl_3_, 25°C, TMS) *δ*=0.98-0.92 (m, 3H; ; CH_2_CH_2_C*H*_3_), 1.25-1.21 (m, 9H; 3CH_2_C*H*_3_), 1.64-1.59 (m, 2H; CH_2_C*H*_2_CH_3_), 2.66 (br, 2H; C*H*_2_CH_2_CH_3_), 4.23-4.17 (m, 6H; 3C*H*_2_CH_3_ ), 6.76 (br, 1H; N*H*), 12.21 (br , 1H; O*H*). **^13^C NMR** (100 MHz, CDCl_3_, 25°C, TMS) *δ*=13.88 (CH_2_*C*H_3_), 14.42 (CH_2_*C*H_3_), 14.51 (2C, CH_2_CH_2_*C*H_3_, CH_2_*C*H_3_), 19.35 (CH_2_*C*H_2_CH_3_), 32.74 (*C*H_2_CH_2_CH_3_), 61.28 (*C*H_2_CH_3_), 62.00 (*C*H_2_CH_3_), 63.19 (*C*H_2_CH_3_), 107.30 (*C*=COH), 156.10 (*C*ONH), 156.91 (*C*ON), 169.99 (*C*OOCH_2_CH_3_), 180.38 (*C*OH). **IR** (KBr) 3287, 2984, 1750, 1714, 1656, 1513, 1323, 1229, 1063. **HRMS** (EI+): *m/z* calcd for C_14_H_24_N_2_O_7_, 332.1584. Found 332.1587.

(*E*)-diethyl 1-(3-ethoxy-1-hydroxy-3-oxo-1-phenylprop-1-en-2-yl)hydrazine-1,2-dicarboxylate(**3f**):


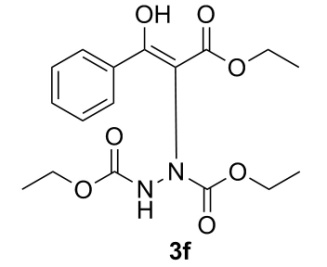


**(*E*)-diethyl 1-(3-ethoxy-1-hydroxy-3-oxo-1-phenylprop-1-en-2-yl)hydr-azine-1,2-dicarboxylate (3f):**  White powder, 95 % yield; M.p. 84-86 ^o^C. **^1^H NMR** (400 MHz, CDCl_3_, 25°C, TMS) *δ*=1.25-1.16 (m, 9H; ; 3), 4.02 (m, 2H), 4.24 (m, 4H; 2C*H*_2_CH_3_), 6.47 (br, 1H; N*H*), 7.14 (br, 1H; O*H*), 7.48-7.47 (d, *J* = 6.80, 2H; Ar-*H*), 7.583 (m, 1H; Ar-*H*), 8.03-8.01 (d, *J* = 7.20, 2H; Ar-*H*). **^13^C NMR** (100 MHz, CDCl_3_, 25°C, TMS) *δ*=14.77 (2C, CH_2_*C*H_3_), 15.10 (CH_2_*C*H_3_),, 62.69 (*C*H_2_CH_3_), 63.09 (*C*H_2_CH_3_), 64.47 (*C*H_2_CH_3_), 129.51 (2C, *C*_6_H_5_), 129.75 (2C, *C*_6_H_5_), 134.88 (*C*_6_H_5_), 135.89 (*C*=COH), 155.90 (*C*ON), 156.79 (*C*ONH), 168.68 (*C*OOCH_2_CH_3_), 191.76 (*C*OH). **IR** (KBr) 3302, 2986, 1756, 1696, 1504,1315, 1209, 1031. **HRMS** (ESI): *m/z* calcd for C_17_H_22_N_2_O_7_ + H, 367.1505[M+H]^+^. Found 367.1502.

(*E*)-diisopropyl 1-(1-ethoxy-3-hydroxy-1-oxobut-2-en-2-yl)hydrazine-1,2-dicarboxylate(**3g**):


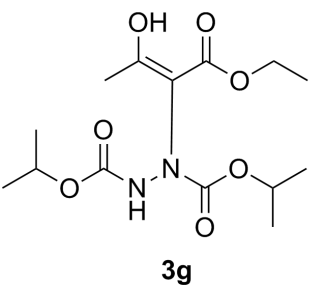


**(*E*)-diisopropyl1-(1-ethoxy-3-hydroxy-1-oxobut-2-en-2-yl)hydrazine-,2-dicarboxy-late (3g):** White powder, 97 % yield; M.p. 74-75 ^o^C. **^1^H NMR** (400 MHz, CDCl_3_, 25°C, TMS) *δ*=1.26-1.16 (m, 15H; 5C*H*_3_), 2.34-2.27 (m, 3H; C*H*_3_), 4.25-4.21 (m, 2H; CH_2_C*H*_3_), 4.93-4.92 ( m, 2H; 2C*H*(CH_3_)_2_), 6.71 (br, 1H; N*H*), 12.08 (br , 1H; O*H*). **^13^C NMR** (100 MHz, CDCl_3_, 25°C, TMS) *δ*=14.48 (CH_2_*C*H_3_), 18.21 (*C*H_3_), 22.00 (2C, CH(*C*H_3_)_2_), 22.12 (2C, CH(*C*H_3_)_2_), 61.28 (*C*H_2_CH_3_), 69.90 (*C*H(CH_3_)_2_), 71.02 (*C*H(CH_3_)_2_), 107.56 (*C*=COH), 155.94 (*C*ONH), 156.18 (*C*ON), 169.91 (*C*OOCH_2_CH_3_), 177.50 (*C*OH). **IR** (KBr) 3279, 2985, 2940, 1749, 1708, 1652, 1506, 1240, 1109 cm^-1^. **HRMS** (EI+): *m/z* calcd for C_14_H_24_N_2_O_7_, 332.1584. Found 332.1575.

(*E*)-diisopropyl 1-(1-(tert-butoxy)-3-hydroxy-1-oxobut-2-en-2-yl)hydrazine-1,2-dicarboxylate(**3h**):


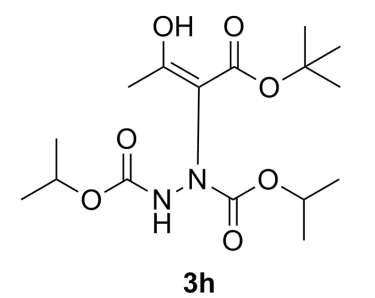


**(*E*)-diisopropyl 1-(1-(tert-butoxy)-3-hydroxy-1-oxobut-2-en-2-yl)hydra-zine-1,2-dicarboxylate (3h):** White powder, 94 % yield; M.p. 69-70 ^o^C. **^1^H NMR** (400 MHz, CDCl_3_, 25°C, TMS) *δ*=1.26-1.20 (m, 12H; 2CH(C*H*_3_)_2_), 1.47-1.44 (m, 9H; C(C*H*_3_)_3_), 2.35-2.24 (m, 3H; C*H*_3_), 4.95-4.91 ( m, 2H; 2C*H*(CH_3_)_2_ ), 6.55 (br, 1H; N*H*), 12.22 (br , 1H; O*H*). **^13^C NMR** (100 MHz, CDCl_3_, 25°C, TMS) *δ*=18.17 (*C*H_3_), 21.99 (2C, CH(*C*H_3_)_2_), 22.12 (2C, CH(*C*H_3_)_2_), 28.48 (3C, C(*C*H_3_)_3_), 69.90 (*C*H(CH_3_)_2_), 70.86 (*C*H(CH_3_)_2_), 82.90 (*C*(CH_3_)_3_), 108.40 (*C*=COH), 156.01 (*C*ONH), 156.40 (*C*ON), 169.70 (*C*OOCH_2_CH_3_), 177.13 (*C*OH). **IR** (KBr) 3283, 2984, 1748, 1707, 1642, 1504, 1369, 1252, 1162, 1111, 1045. **HRMS** (ESI): *m/z* calcd for C_16_H_28_N_2_O_7_ + H, 361.1974. Found 361.1969 [M+H]^+^

(*E*)-dibenzyl 1-(1-ethoxy-3-hydroxy-1-oxobut-2-en-2-yl)hydrazine-1,2-dicarboxylate(**3i**):


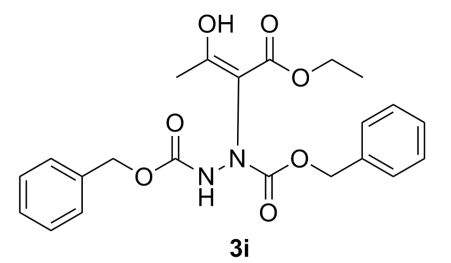


**(*E*)-dibenzyl 1-(1-ethoxy-3-hydroxy-1-oxobut-2-en-2-yl)hydrazine-1,2-dicarboxy-late (3i):** White powder, 95 % yield; M.p. 60-62 ^o^C. **^1^H NMR** (400 MHz, CDCl_3_, 25°C, TMS) *δ*=1.23-1.11 (m, 3H; CH_2_C*H*_3_), 2.43-1.95 (m, 3H; C*H*_3_), 4.20-4.16 (m, 2H; C*H*_2_CH_3_), 5.27-5.19 (m, 4H; C*H*_2_), 7.14 (br, 1H; N*H*), 7.35-7.28 ( m, 10H ), 12.19 (br, 1H; O*H*). **^13^C NMR** (100 MHz, CDCl_3_, 25°C, TMS) *δ*=14.14 (CH_2_*C*H_3_), 18.16 (*C*H_3_), 61.30 (*C*H_2_CH_3_), 67.71 (*C*H_2_Ph), 68.59 (*C*H_2_Ph), 107.29 (*C*=COH), 127.93, 128.29, 128.51(4C), 128.57 (*C*_6_H_5_), (4C, *C*_6_H_5_), 135.54 (*C*_6_H_5_), 135.74 (*C*_6_H_5_), 155.87 (*C*_6_H_5_), (*C*ONH), 156.44 (*C*ON), 169.59 (*C*OH) (*C*OOCH_2_CH_3_), 177.68. **IR** (KBr) 3282, 3002, 1746, 1698, 1607, 1514, 1413, 1332, 1231, 1179, 1057. **HRMS** (ESI): *m/z* calcd for C_22_H_24_N_2_O_7_ + H, 429.1659[M+H]^+^. Found 429.1652.

(*E*)-diethyl 1-(2-hydroxy-4-oxopent-2-en-3-yl)hydrazine-1,2-dicarboxylate (**5a**):

**(*E*)-diethyl 1-(2-hydroxy-4-oxopent-2-en-3-yl)hydrazine-1,2-dicarboxy late (5a):** White powder, 99 % yield; M.p. 126-128 ^o^C; **^1^H NMR** (400 MHz, CDCl_3_, 25°C, TMS) *δ*=1.27-1.22(m, 6H; 2CH_2_C*H*_3_), 2.40-2.05 (m, 6H; 2C*H*_3_), 4.24-4.14 (m, 4H; 2C*H*_2_CH_3_), 7.38 (br, 1H; N*H* ), 16.02 (s, 1H; O*H*). **^13^C NMR** (100 MHz, CDCl_3_, 25°C, TMS) *δ*=14.51 (CH_2_*C*H_3_), 14.64 (CH_2_*C*H_3_), 22.24(2C, *C*H_3_), 62.36 (*C*H_2_CH_3_), 63.68 (*C*H_2_CH_3_), 118.04 (*C*=COH), 155.09 (*C*ONH), 156.52 (*C*ON), 192.12 (2C, *C*OH, CH_3_*C*O). **IR** (KBr) 3263, 2984, 1759, 1697,1615, 1528, 1334, 1244, 1064. **HRMS** (EI+): *m/z* calcd for C_11_H_18_N_2_O_6_, 274.1165. Found 274.1164

*(E*)-diisopropyl 1-(2-hydroxy-4-oxopent-2-en-3-yl)hydrazine-1,2-dicarboxylate (**5b**):

***(E*)-diisopropyl 1-(2-hydroxy-4-oxopent-2-en-3-yl)hydrazine-1,2-dicar- boxylate (5b):** White powder, 97 % yield; M.p. 114-116 ^o^C. **^1^H NMR** (400 MHz, CDCl_3_, 25°C, TMS) *δ*=1.26-1.22 (m, 12H; 2CH(C*H*_3_)_2_), 2.37-2.21 (m, 6H; 2C*H*_3_), 4.99-4.96 (m, 2H; C*H*(CH_3_)_2_), 7.10 (br, 1H; N*H*), 15.98 (s, 1H; O*H*). **^13^C NMR** (100 MHz, CDCl_3_, 25°C, TMS) *δ*=21.97(4C, 2CH(*C*H_3_)_2_), 22.15(2C, 2*C*H_3_), 70.11 *C*H(CH_3_)_2_, 71.35 *C*H(CH_3_)_2_, 118.09 (*C*=COH), 155.39 (*C*ONH), 156.21(*C*ON), 192.00 (2C, *C*OH, CH_3_*C*O). **IR** (KBr) 3276, 2987, 1749, 1703, 1617, 1524, 1378, 1252, 1104. **HRMS** (EI+): *m/z* calcd for C_13_H_22_N_2_O_6_, 302.1478. Found 274. 302.1477

(*E*)-di-tert-butyl 1-(2-hydroxy-4-oxopent-2-en-3-yl)hydrazine-1,2-dicarboxylate (**5c**):

**(*E*)-di-tert-butyl 1-(2-hydroxy-4-oxopent-2-en-3-yl)hydrazine-1,2-dica-rboxylate (5c):** White powder, 92 % yield; M.p. 138-139 ^o^C. **^1^H NMR** (400 MHz, CDCl_3_, 25 °C, TMS) *δ*=1.49-1.39 (m, 18H; 2C(C*H*_3_)_3_), 2.40-2.05 (m, 6H; 2C*H*_3_), 6.81 (br, 1H; N*H*), 15.91 (s, 1H; O*H*). **^13^C NMR** (100 MHz, CDCl_3_, 25 ^o^C, TMS) *δ*=22.17 (2C, *C*H_3_), 28.13 (6C, 2C(*C*H_3_)_3_), 81.61 (*C*(CH_3_)_3_), 82.38 (*C*(CH_3_)_3_), 118.56 (*C*=COH), 154.47 (*C*ONH), 155.59 (*C*ON), 191.77 (2C, *C*OH, CH_3_*C*O). **IR** (KBr) 3304, 2983, 1746, 1686, 1616, 1517, 1388, 1265, 1159. **HRMS** (EI+): *m/z* calcd for C_15_H_26_N_2_O_6_, 302. 330.1791. Found 274. 330.1864.

(*E*)-dibenzyl 1-(2-hydroxy-4-oxopent-2-en-3-yl)hydrazine-1,2-dicarboxylate (**5d**):

**(*E*)-dibenzyl 1-(2-hydroxy-4-oxopent-2-en-3-yl)hydrazine-1,2-dicarbo-xylate (5d):** White powder, 94 % yield; M.p. 123-125 ^o^C. **^1^H NMR** (400 MHz, CDCl_3_, 25°C, TMS) *δ*=3.20-3.11 (m, 6H; 2C*H*_3_), 5.20-5.12 (m, 4H; C*H*_2_), 7.34-7.27 (m, 10H; Ar-*H*), 7.46 (br, 1H; N*H*), 16.04 (s, 1H; O*H*). **^13^C NMR** (100 MHz, CDCl_3_, 25°C, TMS) *δ*=23.41 (2C, *C*H_3_), 69.34 (*C*H_2_Ph), 70.45 (*C*H_2_Ph), 119.51 (*C*=COH), 129.55(2C, *C*_6_H_5_), 129.70(4C, *C*_6_H_5_), 130.00(4C, *C*_6_H_5_), 136.62(2C, *C*_6_H_5_), 157.27 (*C*ONH), 157.54 (*C*ON), 193.47 (2C, *C*OH, CH_3_*C*O). **IR** (KBr) 3268, 3023, 1755, 1703, 1605, 1525, 1401, 1337, 1245, 1056. **HRMS** (EI+): *m/z* calcd for C_21_H_22_N_2_O_6_, 302. 398.1478. Found 274. 398.1533.

(*E*)-diethyl 1-(1-hydroxy-3-oxo-1,3-diphenylprop-1-en-2-yl)hydrazine-1,2-dicarboxylate

**(*E*)-diethyl 1-(1-hydroxy-3-oxo-1,3-diphenylprop-1-en-2-yl)hydrazine-1,2-dicarbo- xylate (5e):** White powder, 93 % yield; M.p. 132-134 ^o^C. **^1^H NMR** (400 MHz, CDCl_3_, 25°C, TMS) *δ*=1.17-1.14 (m, 3H; CH_2_C*H*_3_), 1.31-1.25 (m, 3H; CH_2_C*H*_3_), 3.80 (br, 1H; N*H*),4.02-3.99 (m, 2H; C*H*_2_CH_3_), 4.28-4.25(m, 2H; C*H*_2_CH_3_), 7.10 (br, 1H; O*H*), 7.48-7.31 (m, 4H; Ar-*H*), 7.61-7.58 (m, 2H; Ar-*H*), 8.12-7.81 (m, 4H). **^13^C NMR** (100 MHz, CDCl_3_, 25°C, TMS) *δ*=14.41 (2C, CH_2_*C*H_3_), 61.96 (*C*H_2_CH_3_), 63.83 (*C*H_2_CH_3_), 66.47 (*C*=COH), 128.86(4C, *C*_6_H_5_), 129.00(4C, *C*_6_H_5_), 134.27(2C), 135.01(2C, *C*_6_H_5_), 155.30 (*C*ONH), 156.41 (*C*ON), 193.68 (2C, *C*OH, CH_3_*C*O). **IR** (KBr) 3285, 2359, 1759, 1689, 1596, 1497, 1413, 1320, 1217, 1138, 1057. **HRMS** (ESI): *m/z* calcd for C_21_H_22_N_2_O_6_ + H, 399.1556[M+H]^+^. Found 399.1548.

(*E*)-diethyl 1-(1-cyano-2-hydroxy-3,3-dimethylbut-1-en-1-yl)hydrazine-1,2-dicarboxylate (**7a**):

**(*E*)-diethyl1-(1-cyano-2-hydroxy-3,3-dimethylbut-1-en-1-yl)hydrazine-1,2-dicarbo- xylate (7a):** White powder, 89 % yield; M.p. 65-68 ^o^C. **^1^H NMR** (400 MHz, CDCl_3_, 25°C, TMS) *δ*=1.32-1.17 (m, 15H; ; 5C*H*_3_), 4.23-4.18 (m, 4H; 2C*H*_2_CH_3_), 7.62 (br, 1H; N*H*), 10.62 (br, 1H; O*H*). **^13^C NMR** (100 MHz, CDCl_3_, 25°C, TMS) *δ*=13.96 (2C, CH_2_*C*H_3_), 26.09 (C(*C*H_3_)_3_), 27.87 (2C, C(*C*H_3_)_3_), 36.40 (*C*(CH_3_)_3_), 63.40 (*C*H_2_CH_3_), 63.74 (*C*H_2_CH_3_), 90.85 (*C*=COH), 117.70 (*C*N), 154.60 (*C*ONH), 160.87 (*C*ON), 180.77 (*C*OH). **IR** (KBr) 3325, 2986, 2207, 1736, 1583, 1518, 1402, 1267, 1207, 1087, 761, 525. **HRMS** (ESI): *m/z* calcd for C_13_H_21_N_3_O_5_ + H, 300.1559 [M+H]^+^. Found 300.1559.

(*Z*)-diethyl 1-(2-hydroxy-1-nitro-2-phenylvinyl)hydrazine-1,2-dicarboxylate (**7b**):

**(*Z*)-diethyl 1-(2-hydroxy-1-nitro-2-phenylvinyl)hydrazine-1,2-dicarbo-xylate (7b):** White powder, 89 % yield; M.p. 87-89 ^o^C. **^1^H NMR** (400 MHz, CDCl_3_, 25°C, TMS) *δ*=1.44-1.26 (m, 6H; 2CH_2_C*H*_3_), 4.45-4.09 (m, 4H; 2C*H*_2_CH_3_), 7.12 (br, 1H; N*H*), 7.80-7.62 (m, 4H; Ar-*H*), 8.15 (m, 2H; Ar-*H,* O*H*). **^13^C NMR** (100 MHz, CDCl_3_, 25°C, TMS) *δ*=14.14 (2C, 2CH_2_*C*H_3_), 62.54 (*C*H_2_CH_3_), 65.07 (*C*H_2_CH_3_), 93.97 (*C*=COH), 128.97 (*C*_6_H_5_), 129.39 (*C*_6_H_5_), 133.24 (*C*_6_H_5_), 135.18 (*C*_6_H_5_), 154.93 (*C*ONH), 155.48 (*C*ON), 184.21 (*C*OH). **IR** (KBr) 3283, 2990, 2359, 1740, 1708, 1581, 1509, 1285, 1228, 1060. **HRMS** (ESI): *m/z* calcd for C_14_H_17_N_3_O_7_ + H, 340.1144[M+H]^+^. Found 340.1140.

1. ^1^H NMR Spectra for **3a-3i, 5a-5e, 7a,7b**


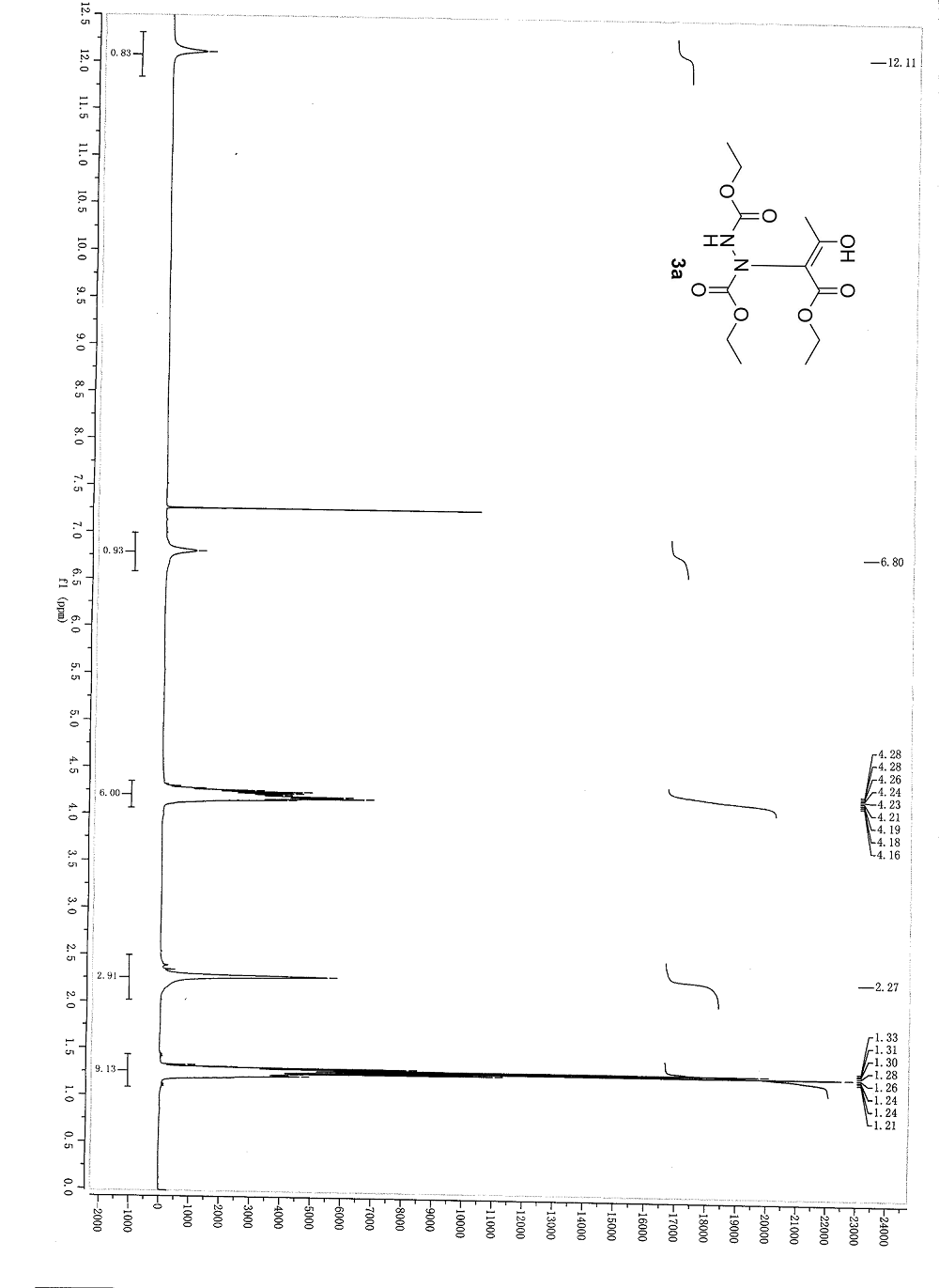


Fig. 1 ^1^H NMR spectrum of product **3a**


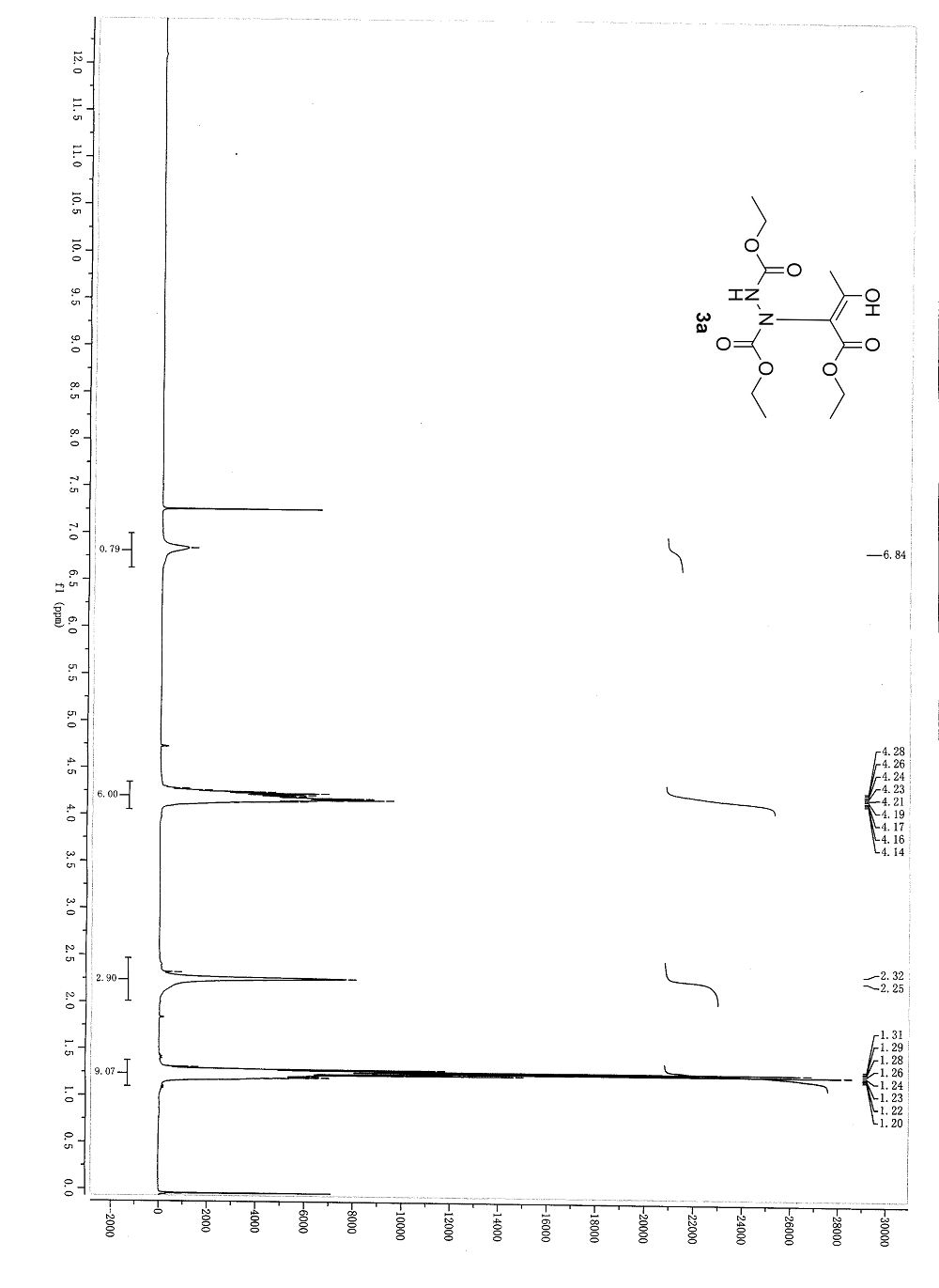


Fig. 2 ^1^H NMR spectrum of product **3a** in CDCl_3_ and one drop D_2_O


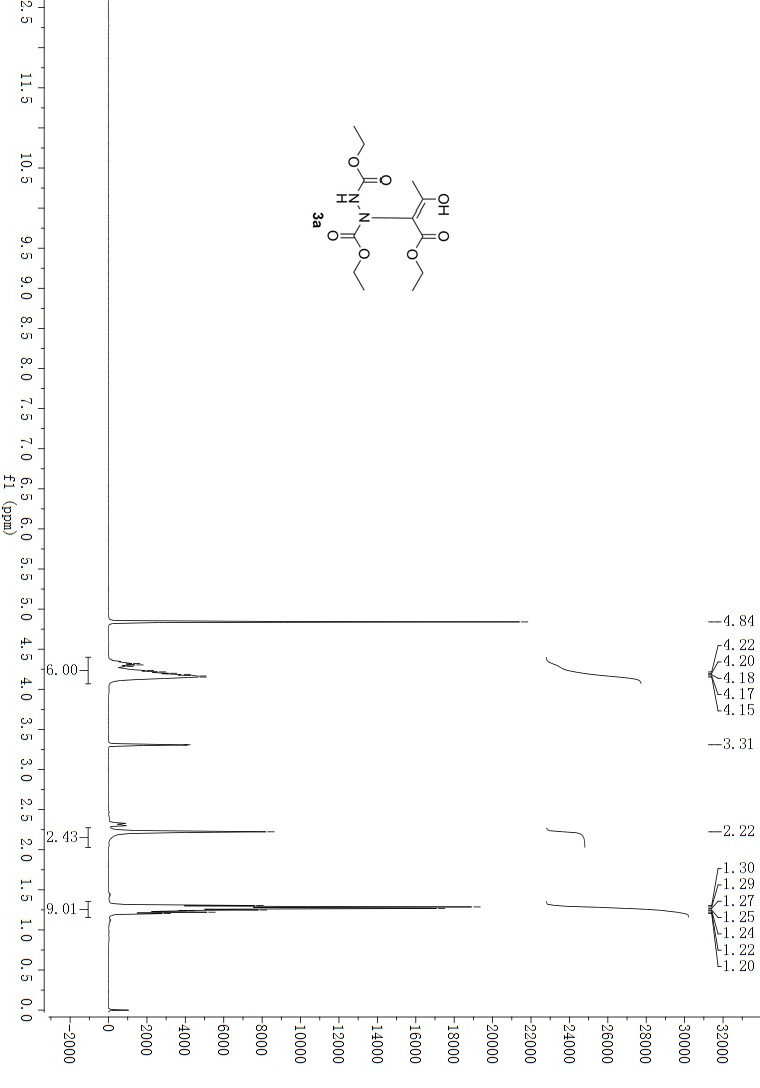


Fig. 3 ^1^H NMR spectrum of product **3a** in CD_3_OD


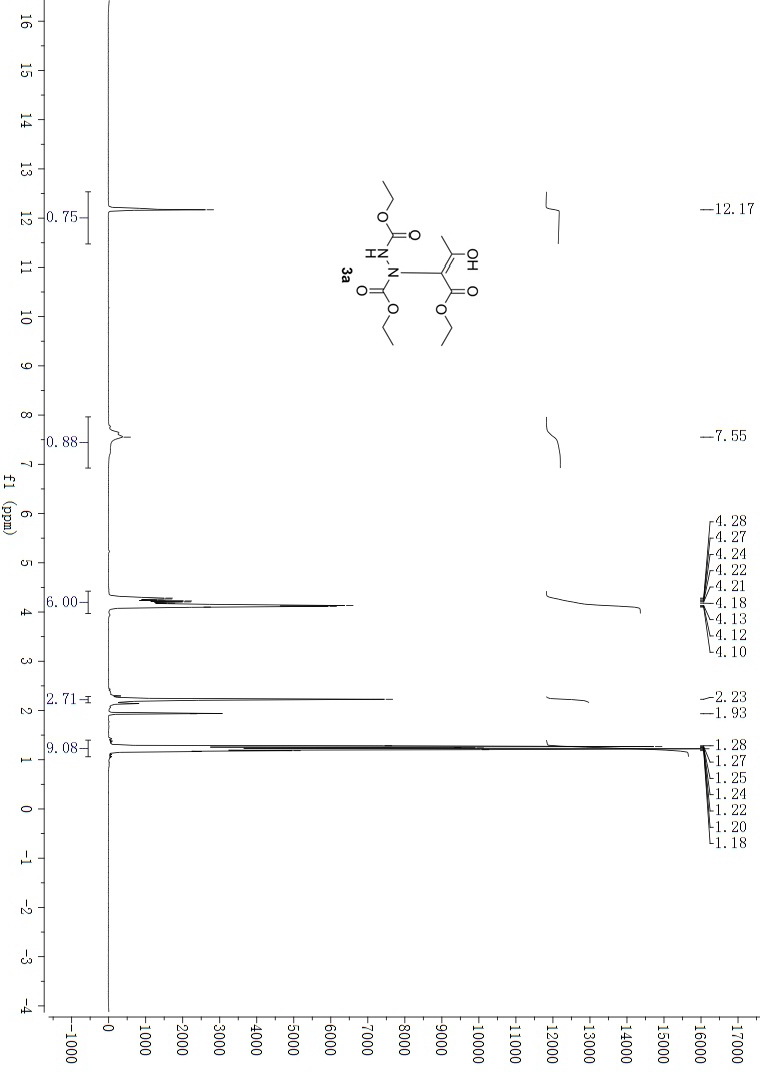


Fig. 4 ^1^H NMR spectrum of product **3a** in CD_3_CN


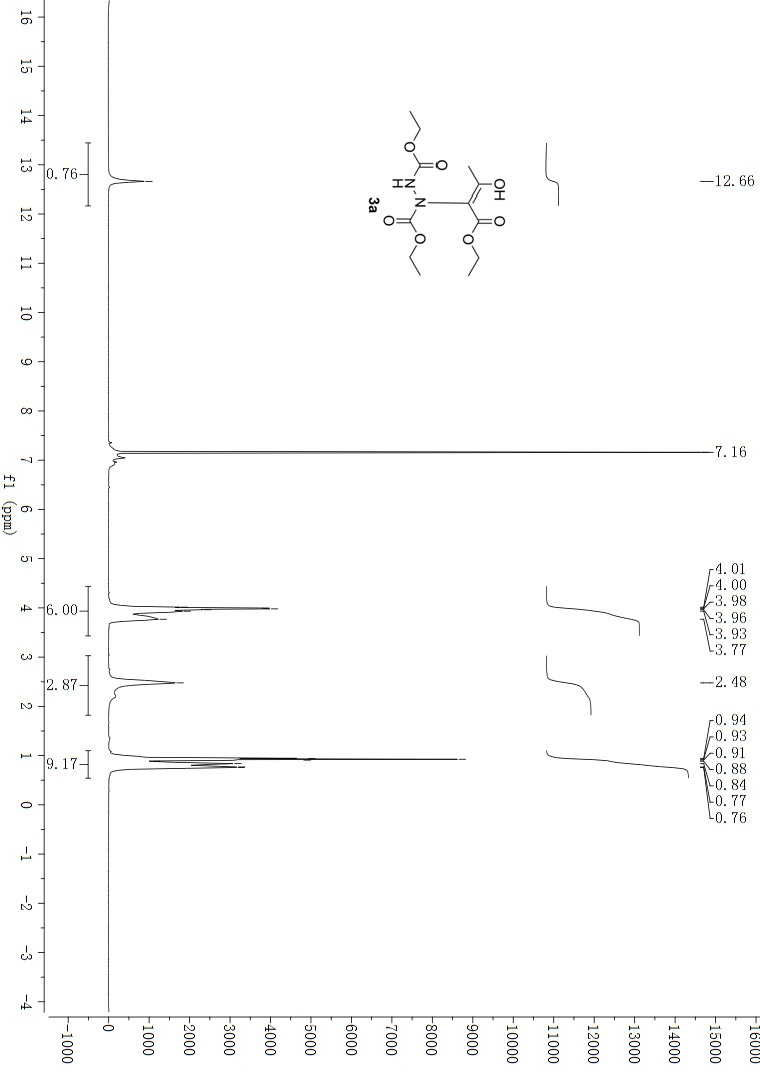


Fig. 5 ^1^H NMR spectrum of product **3a** in C_6_D_6_


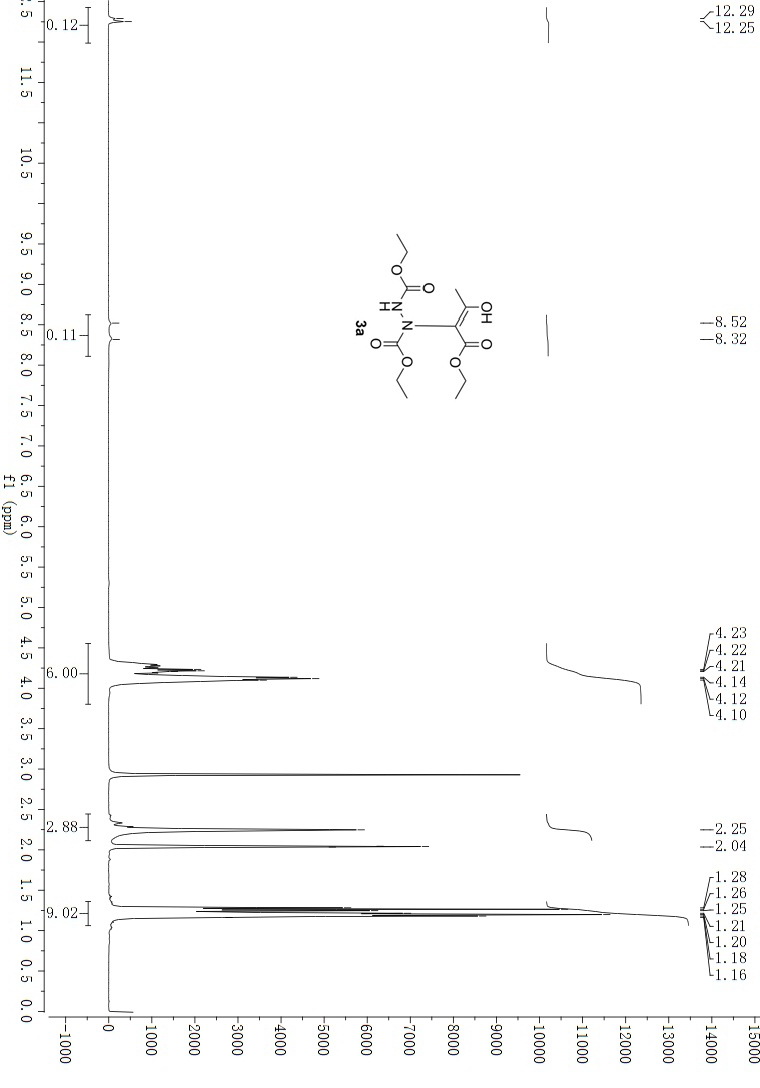


Fig. 6 ^1^H NMR spectrum of product **3a** in CD_3_COCD_3_


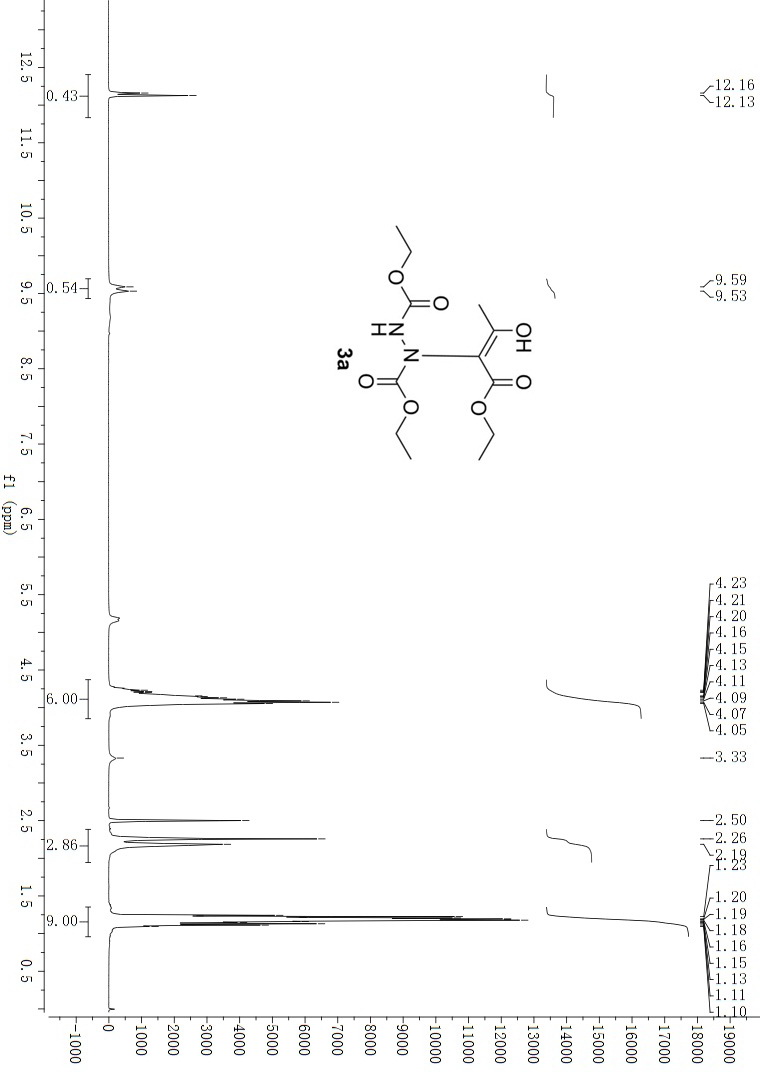


Fig. 7 ^1^H NMR spectrum of product **3a** in CD_3_SOCD_3_


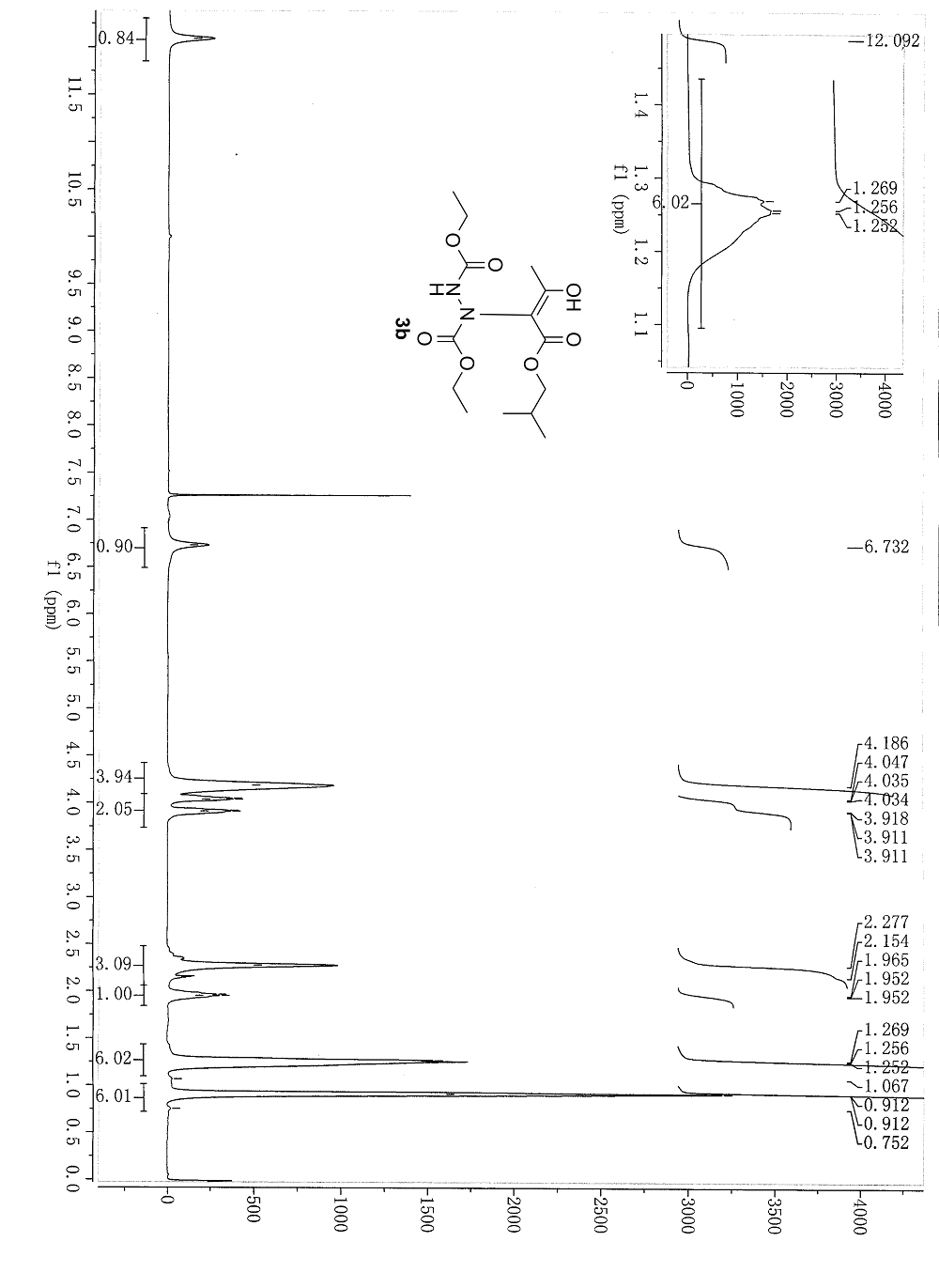


Fig. 8 ^1^H NMR spectrum of product **3b**


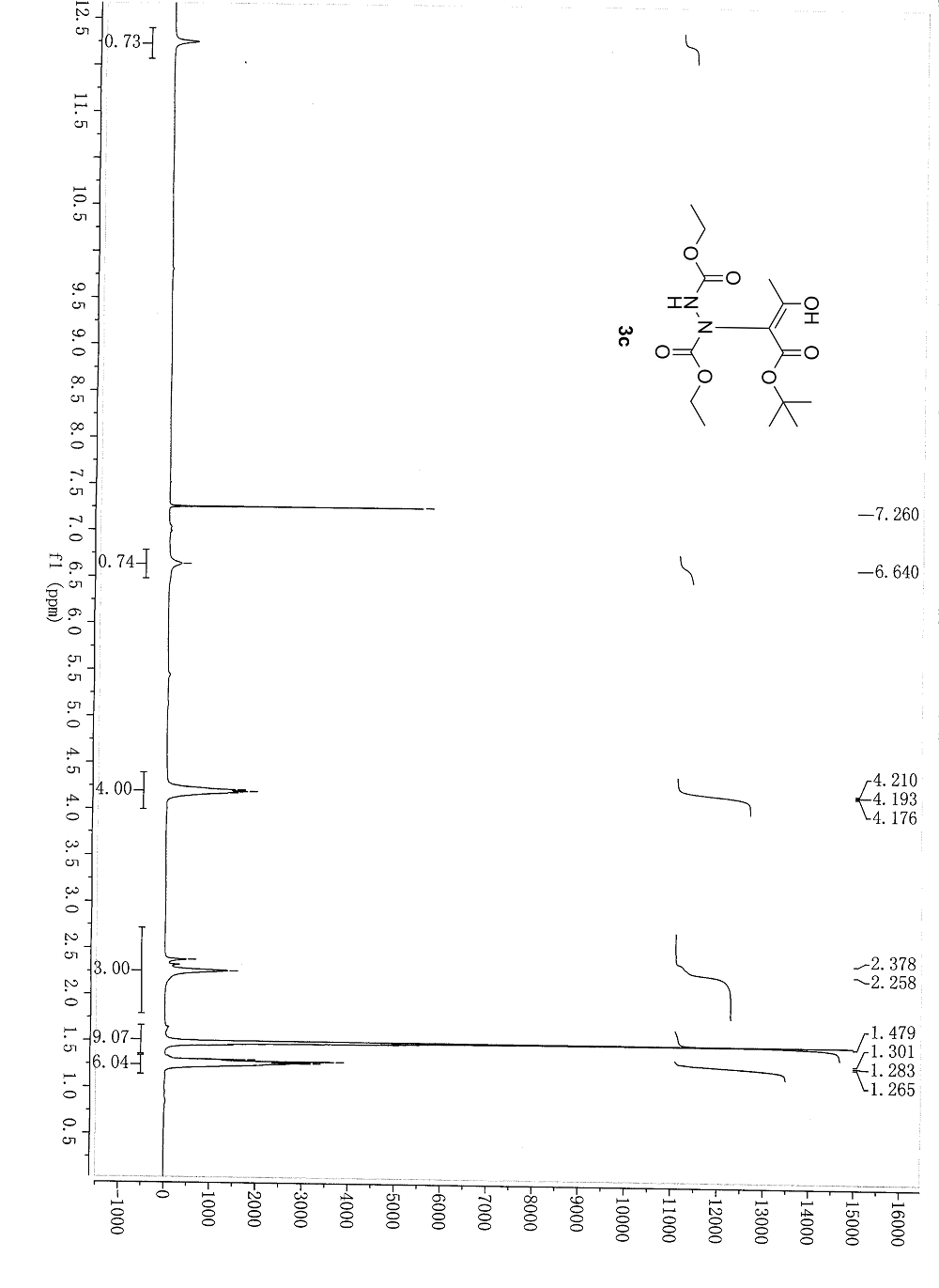


Fig. 9 ^1^H NMR spectrum of product **3c**


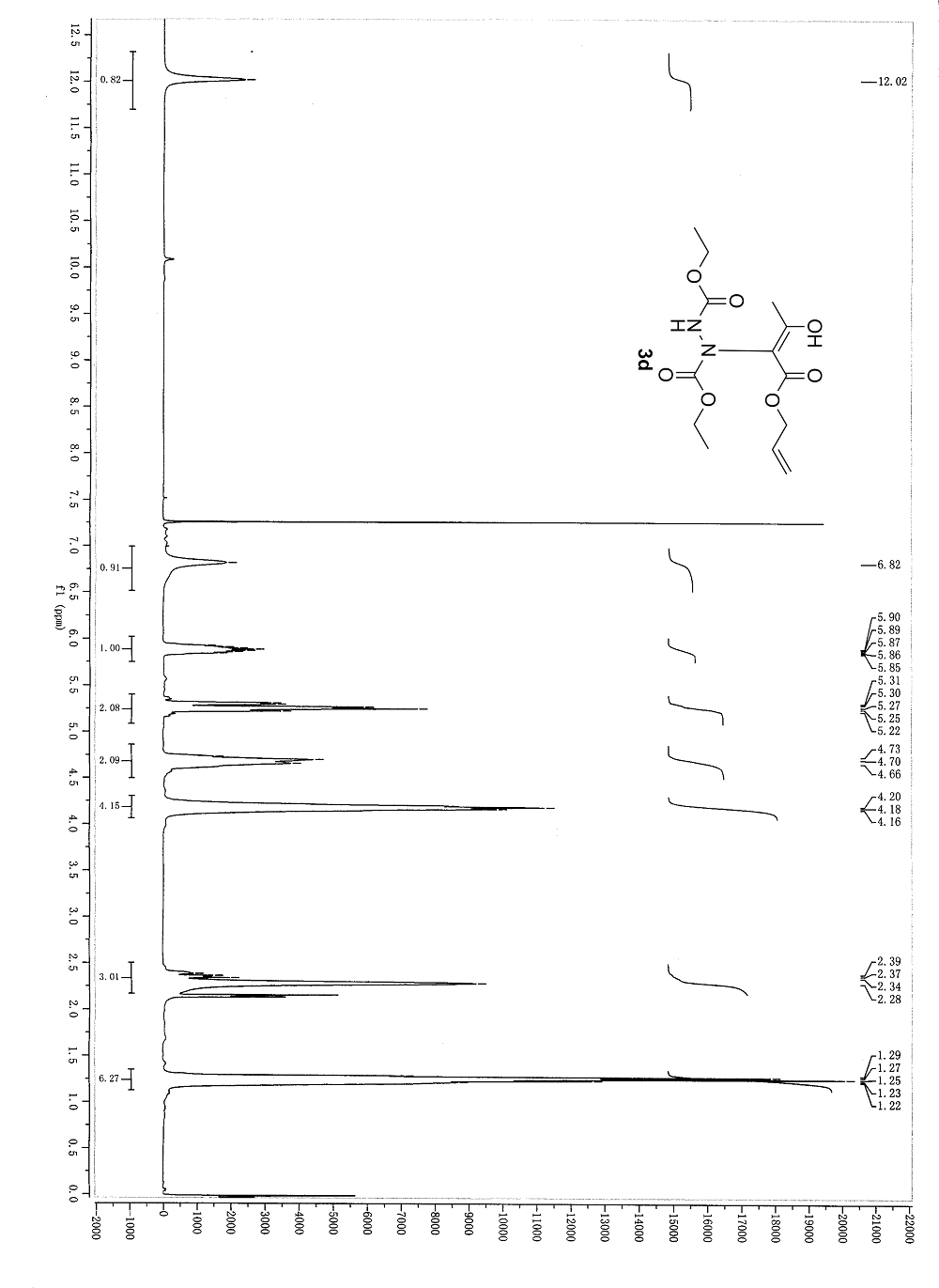


Fig. 10 ^1^H NMR spectrum of product **3d**


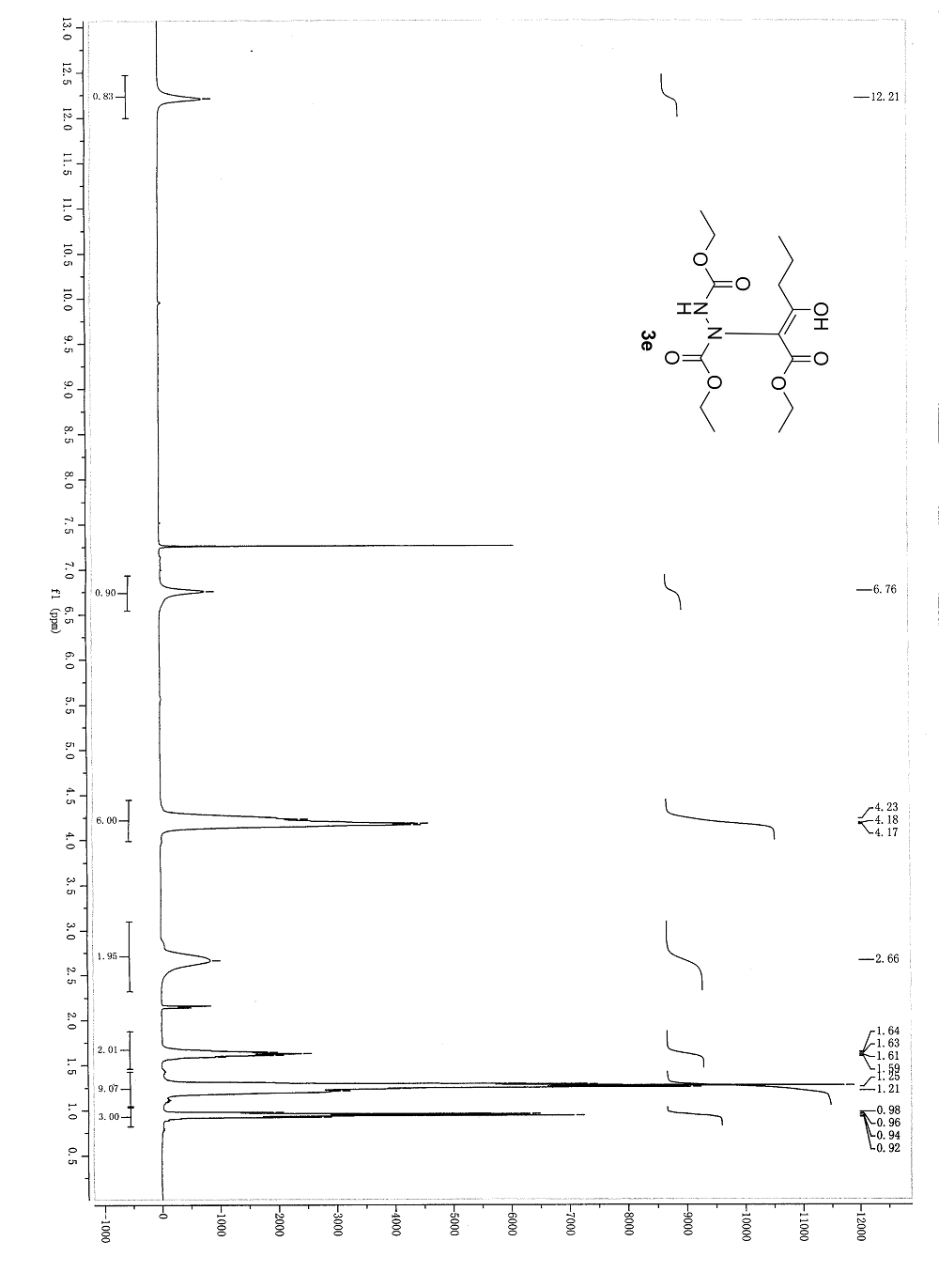


Fig. 11 ^1^H NMR spectrum of product **3e**


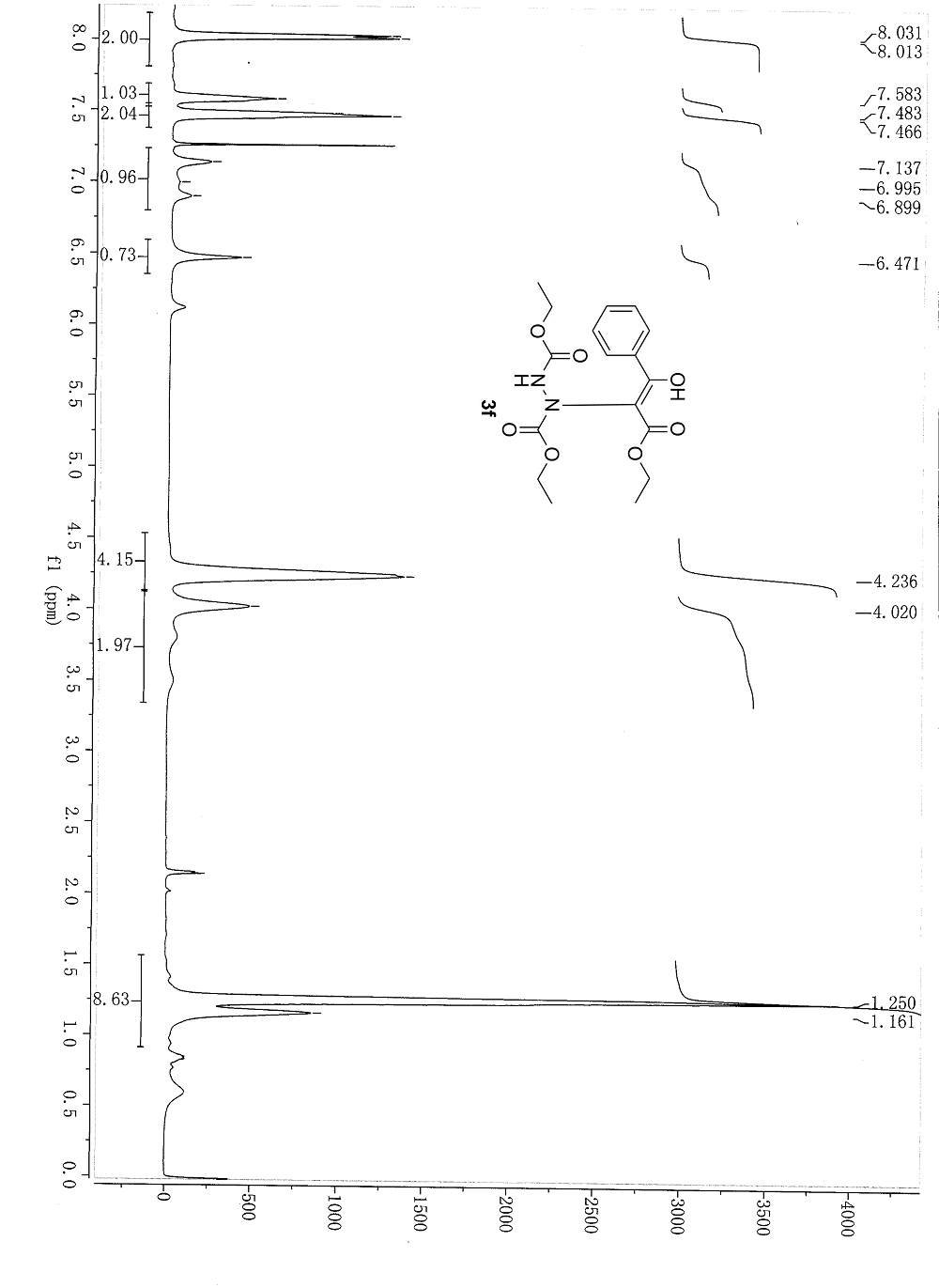


Fig. 12 ^1^H NMR spectrum of product **3f**


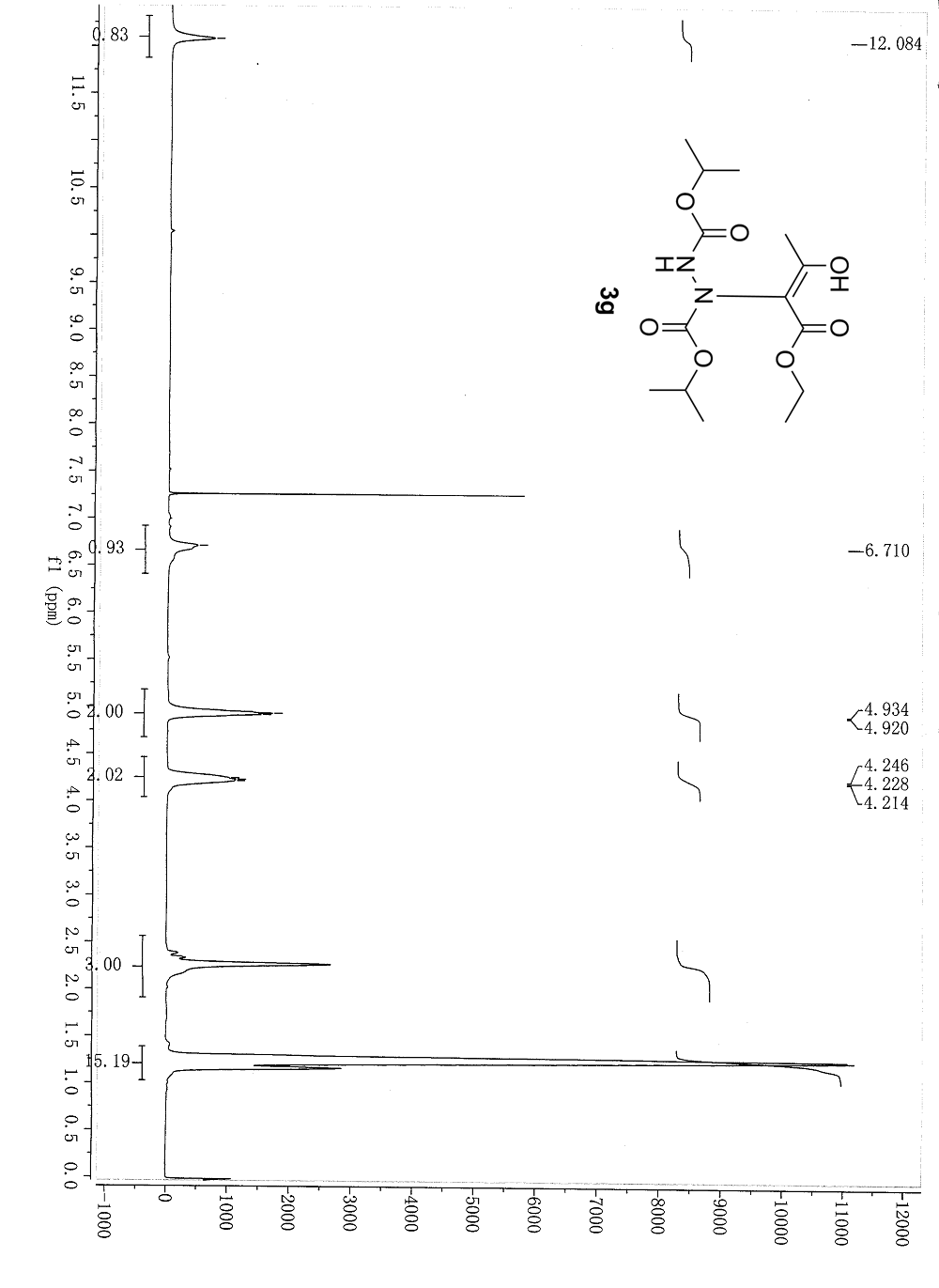


Fig. 13 ^1^H NMR spectrum of product **3g**


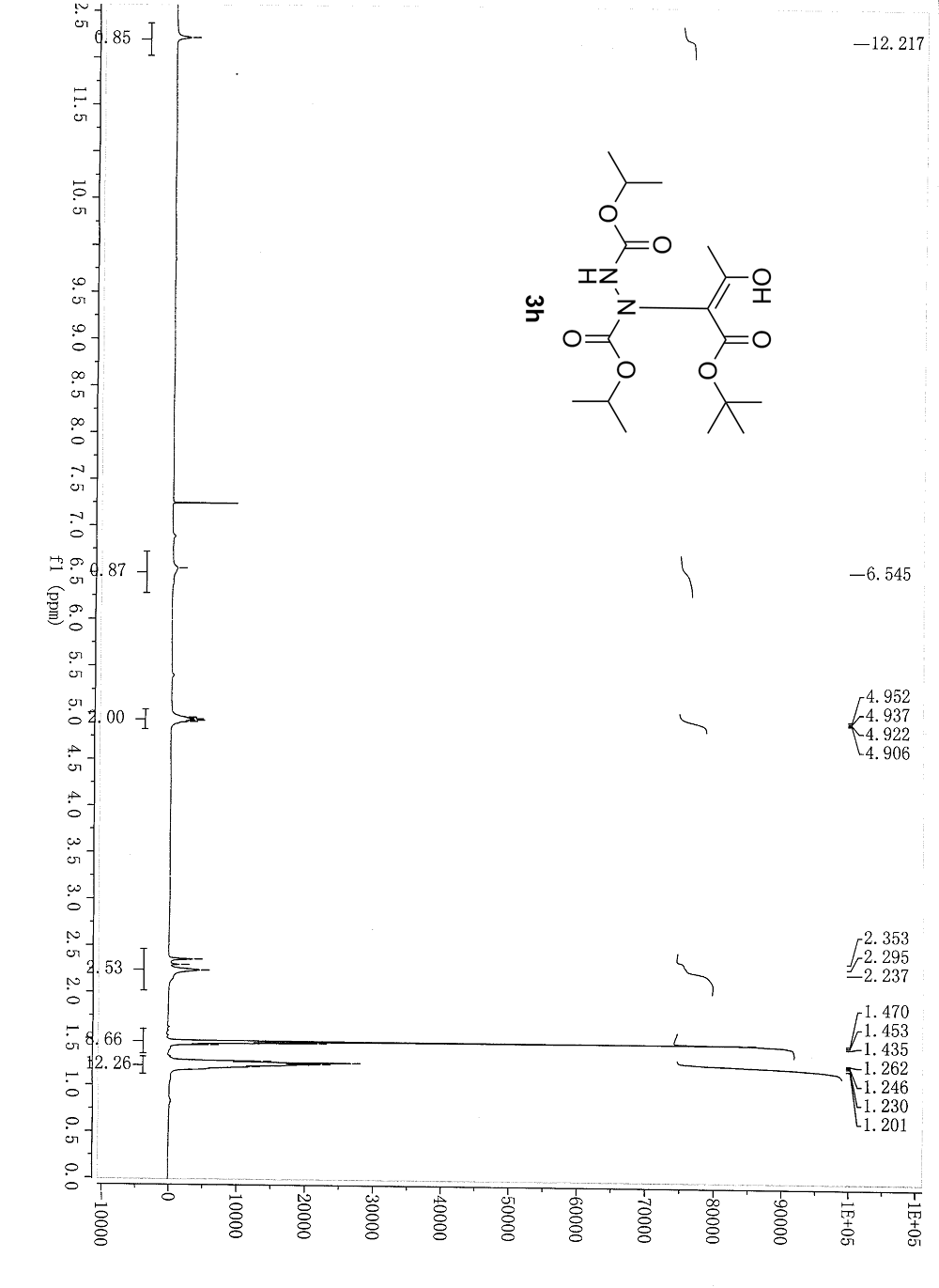


Fig. 14 ^1^H NMR spectrum of product **3h**


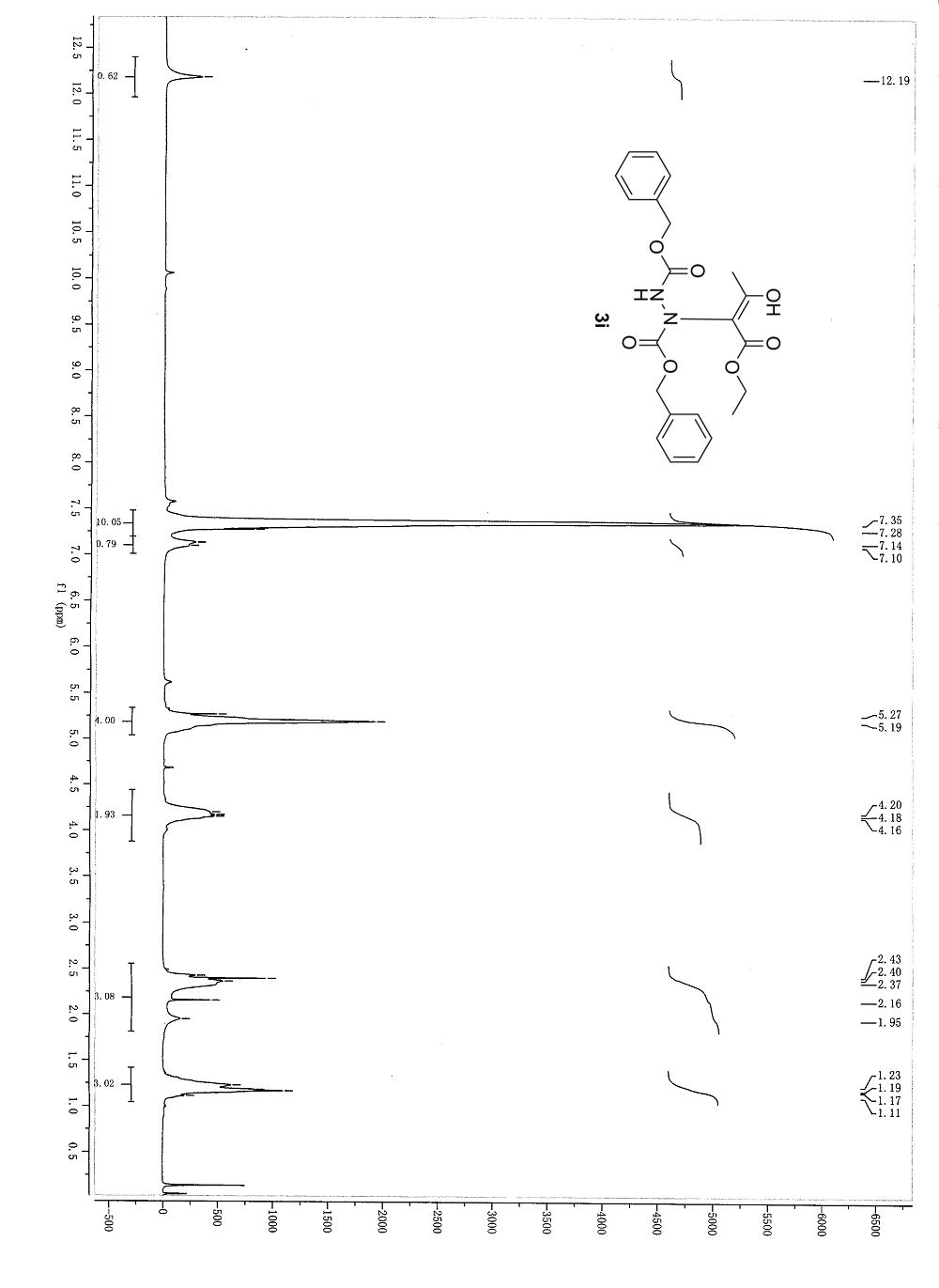


Fig. 15 ^1^H NMR spectrum of product **3i**


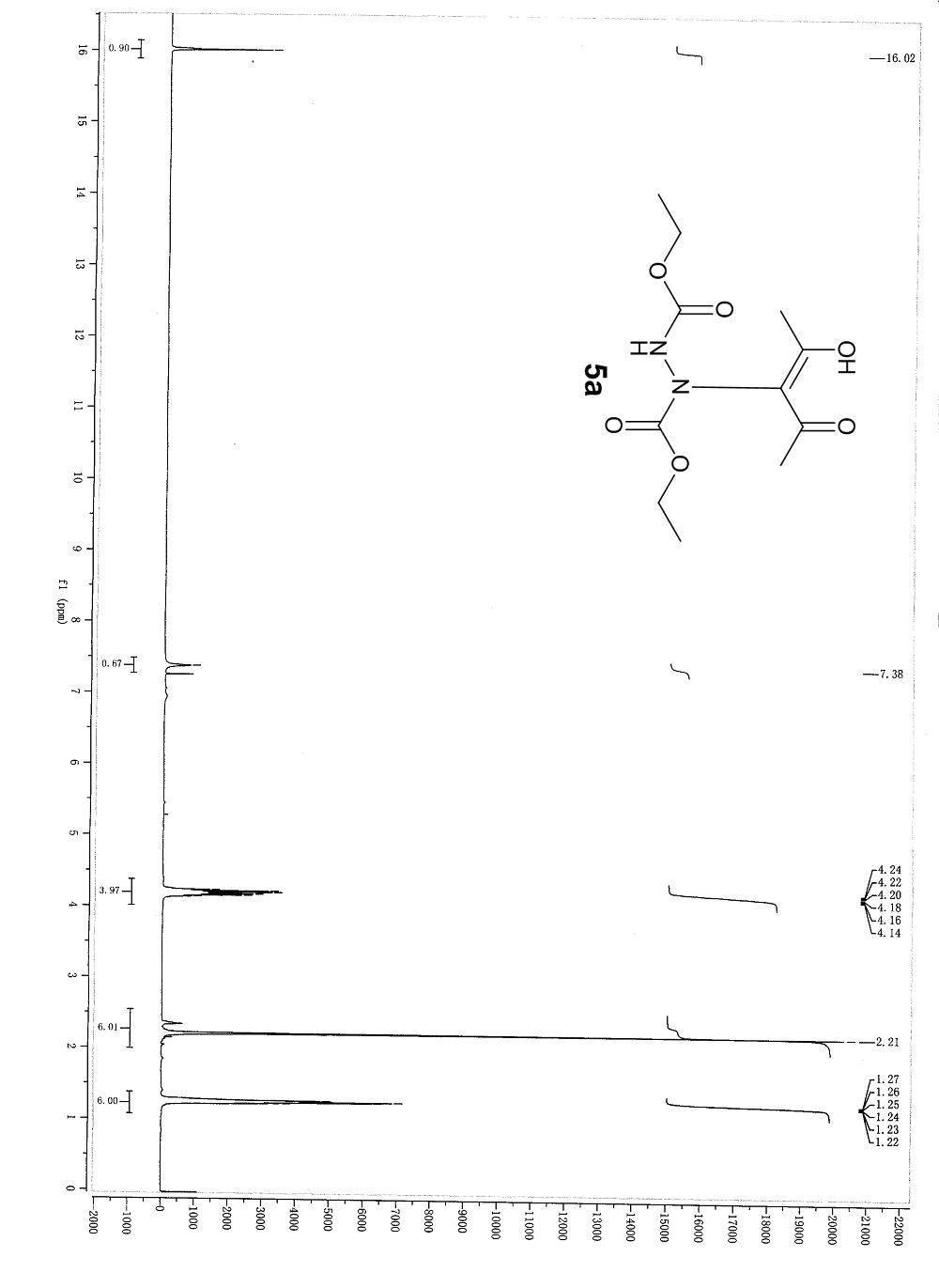


Fig. 16 ^1^H NMR spectrum of product **5a**

**
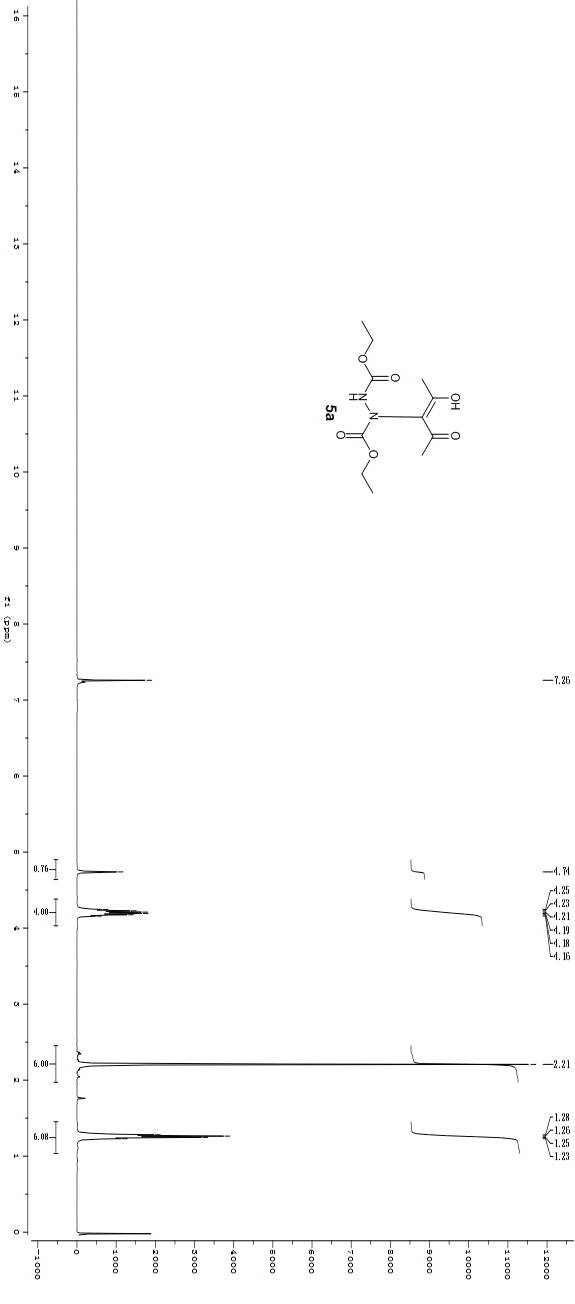
**

Fig. 17 ^1^H NMR spectrum of product **5a** in CDCl_3_ and one drop D_2_O


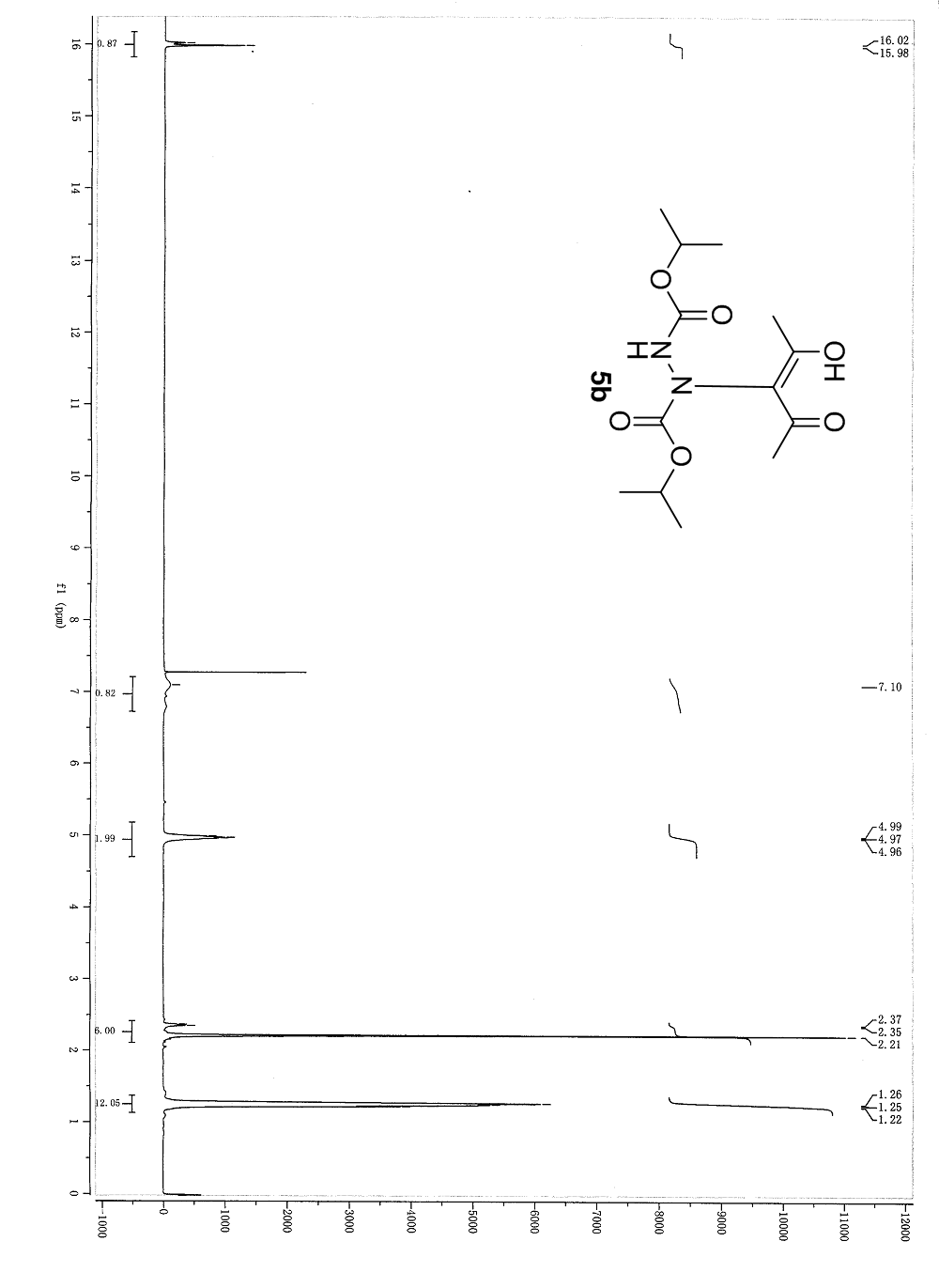


Fig. 18 ^1^H NMR spectrum of product **5b**


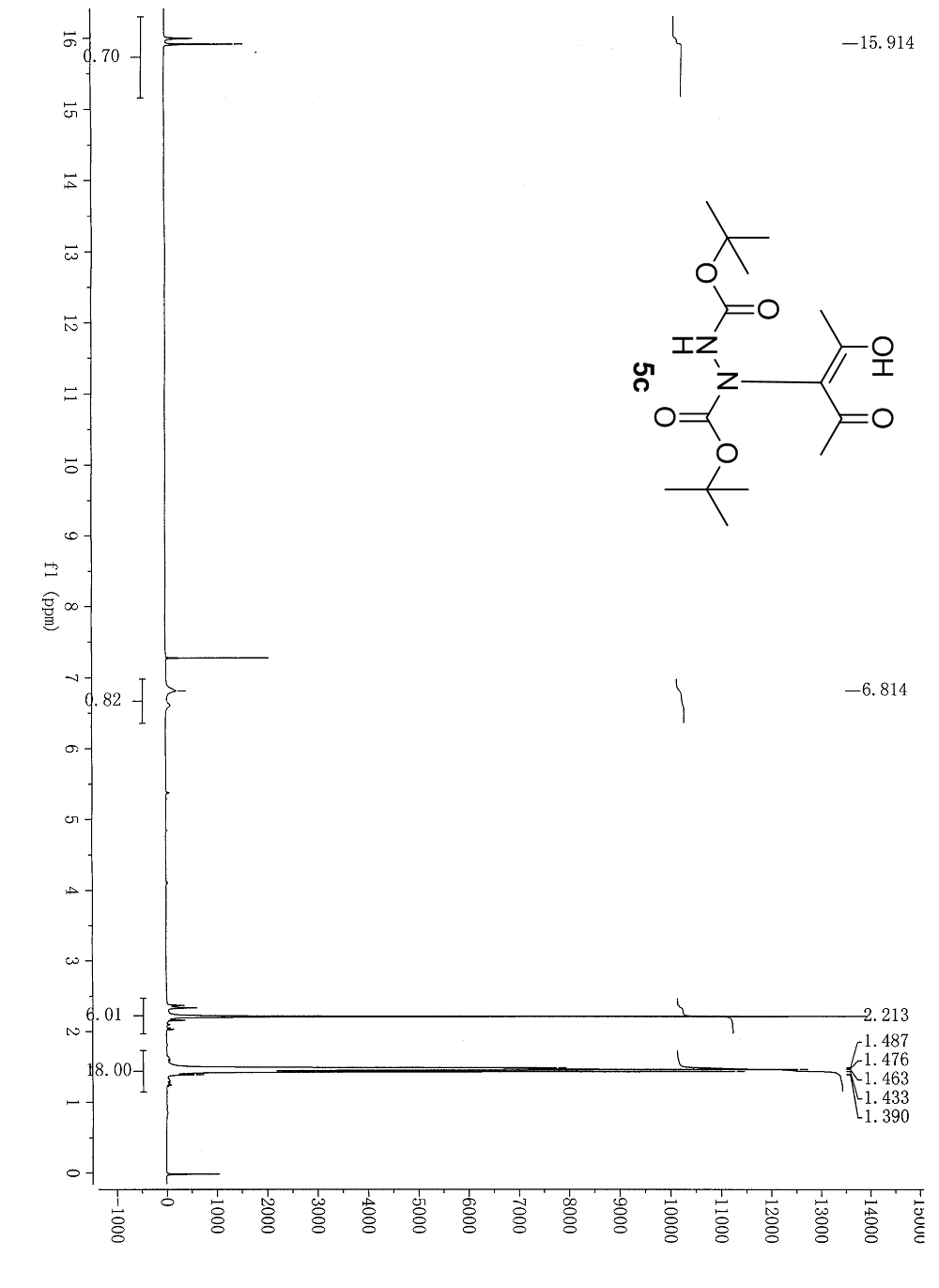


Fig. 19 ^1^H NMR spectrum of product **5c**


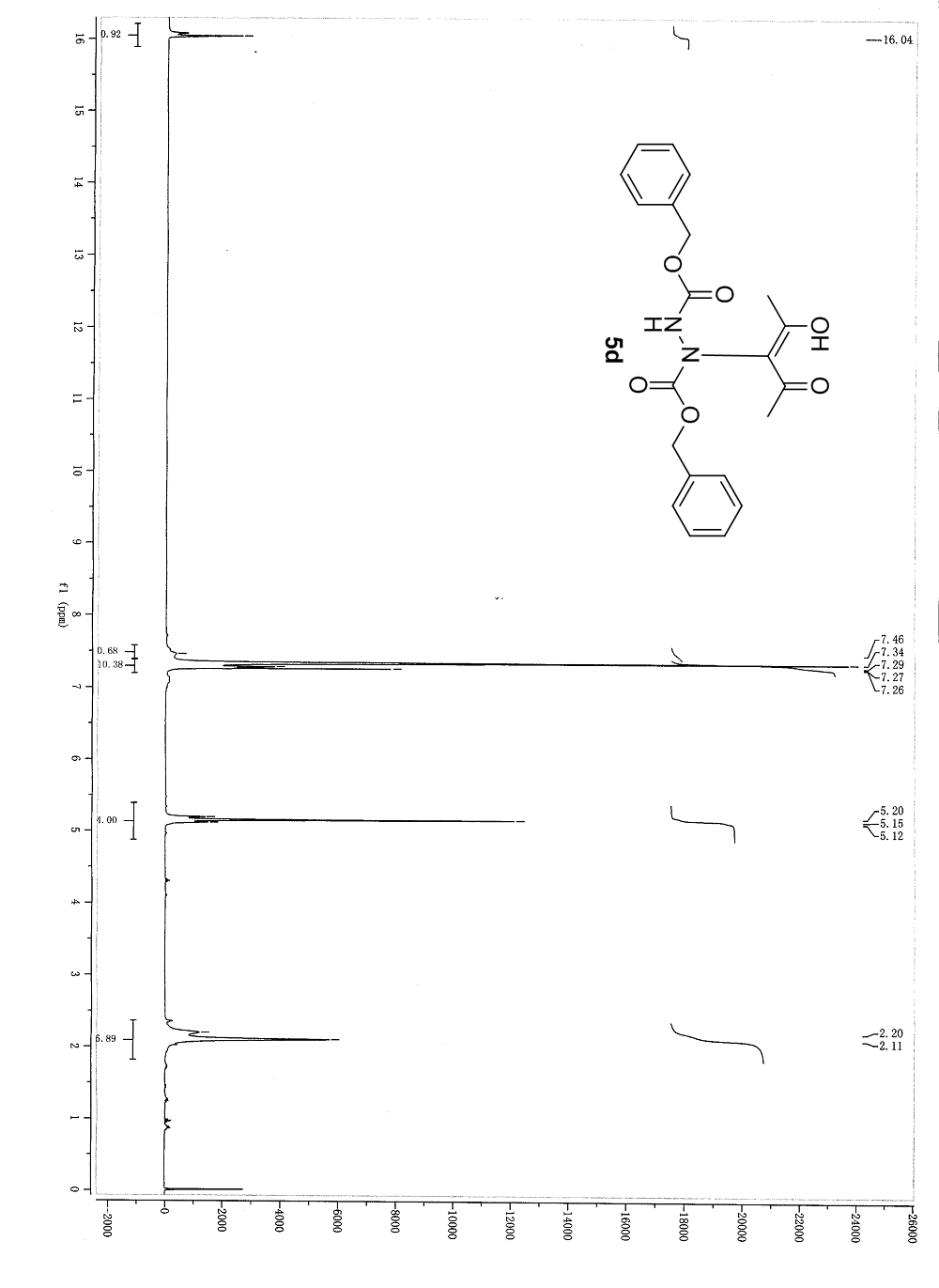


Fig. 20 ^1^H NMR spectrum of product **5d**


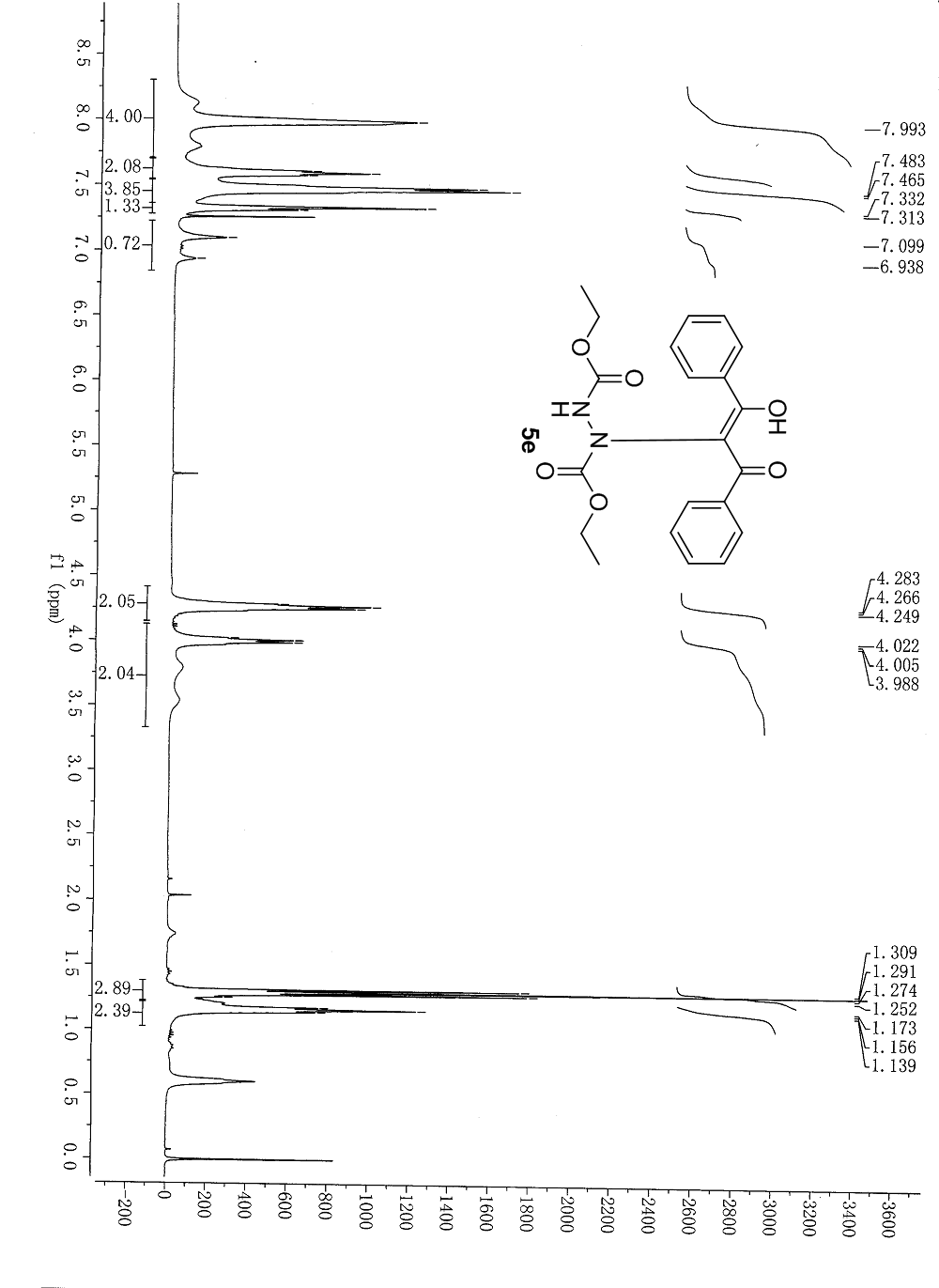


Fig. 21 ^1^H NMR spectrum of product **5e**


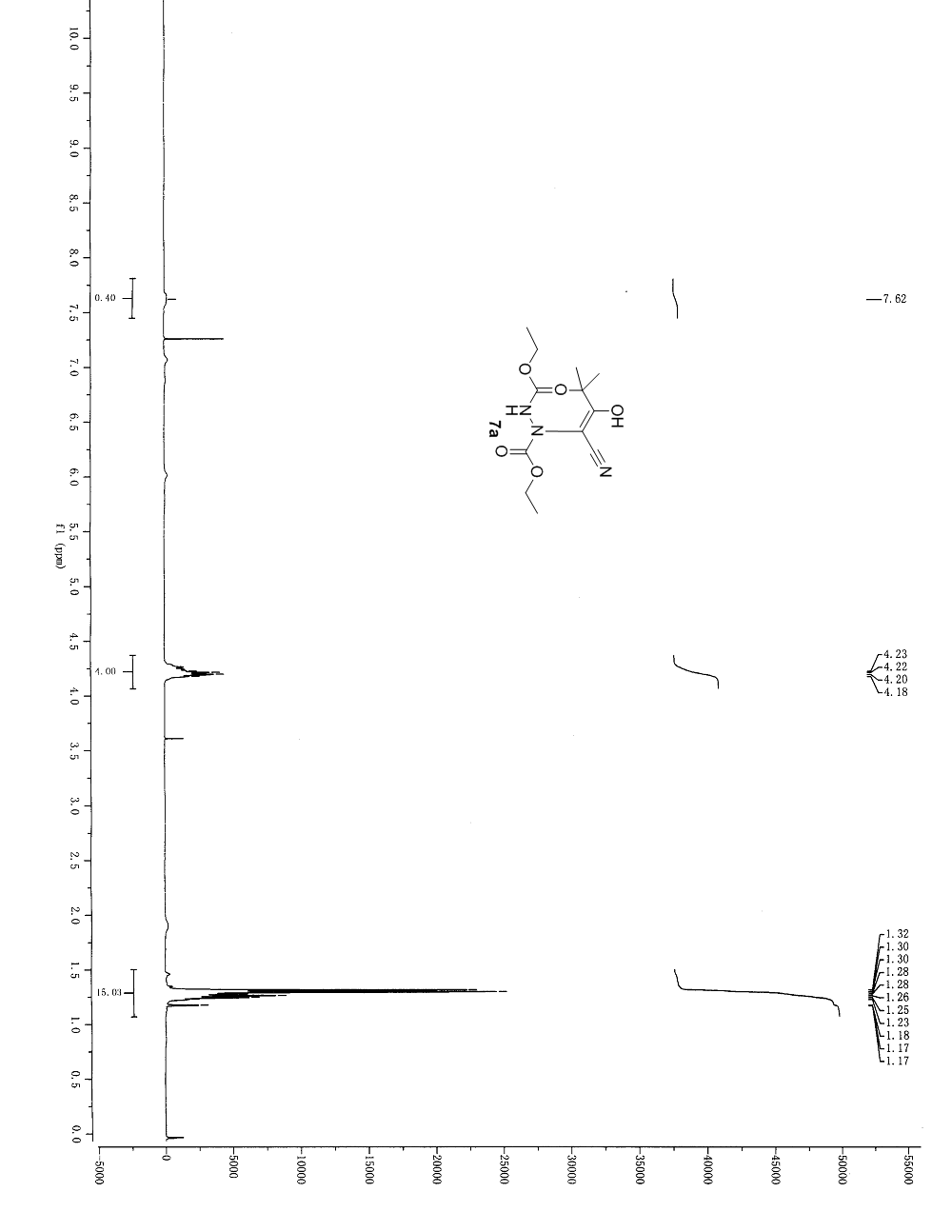


Fig. 22 ^1^H NMR spectrum of product **7a**


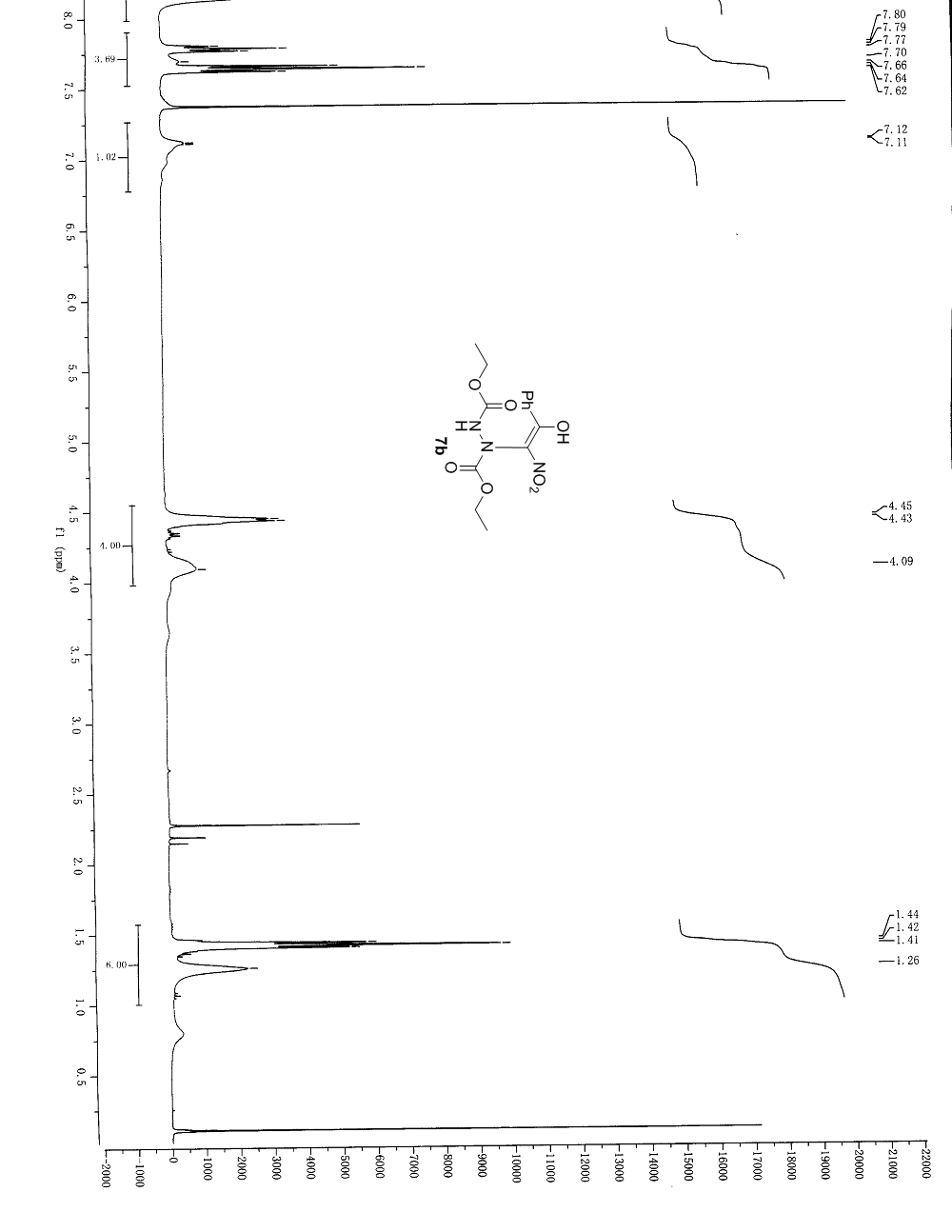


Fig. 23 ^1^H NMR spectrum of product **7b**

1. ^13^C NMR Spectra for **3a-3i, 5a-5e, 7a,7b**


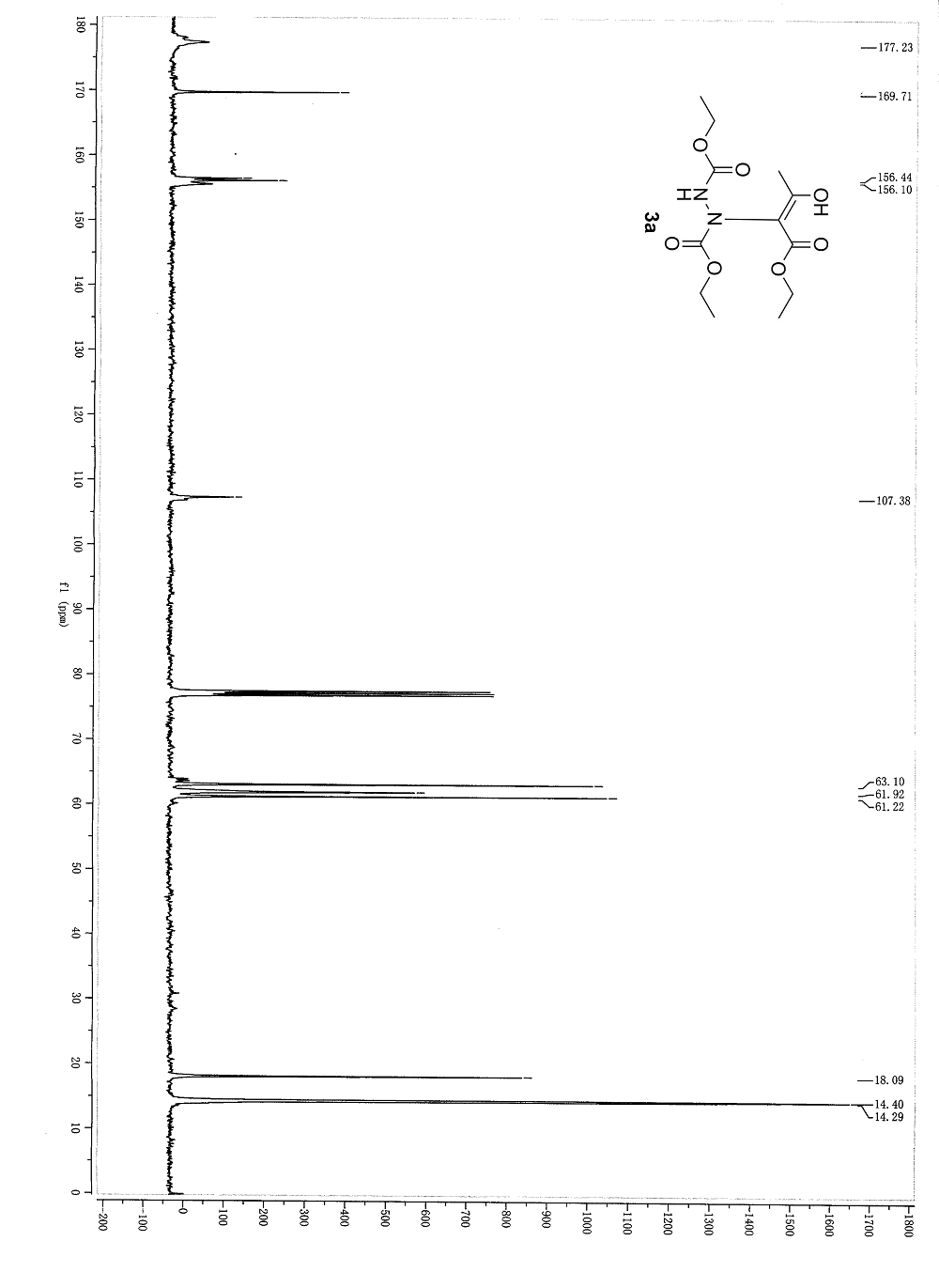


Fig. 24 ^13^C NMR spectrum of product **3a**


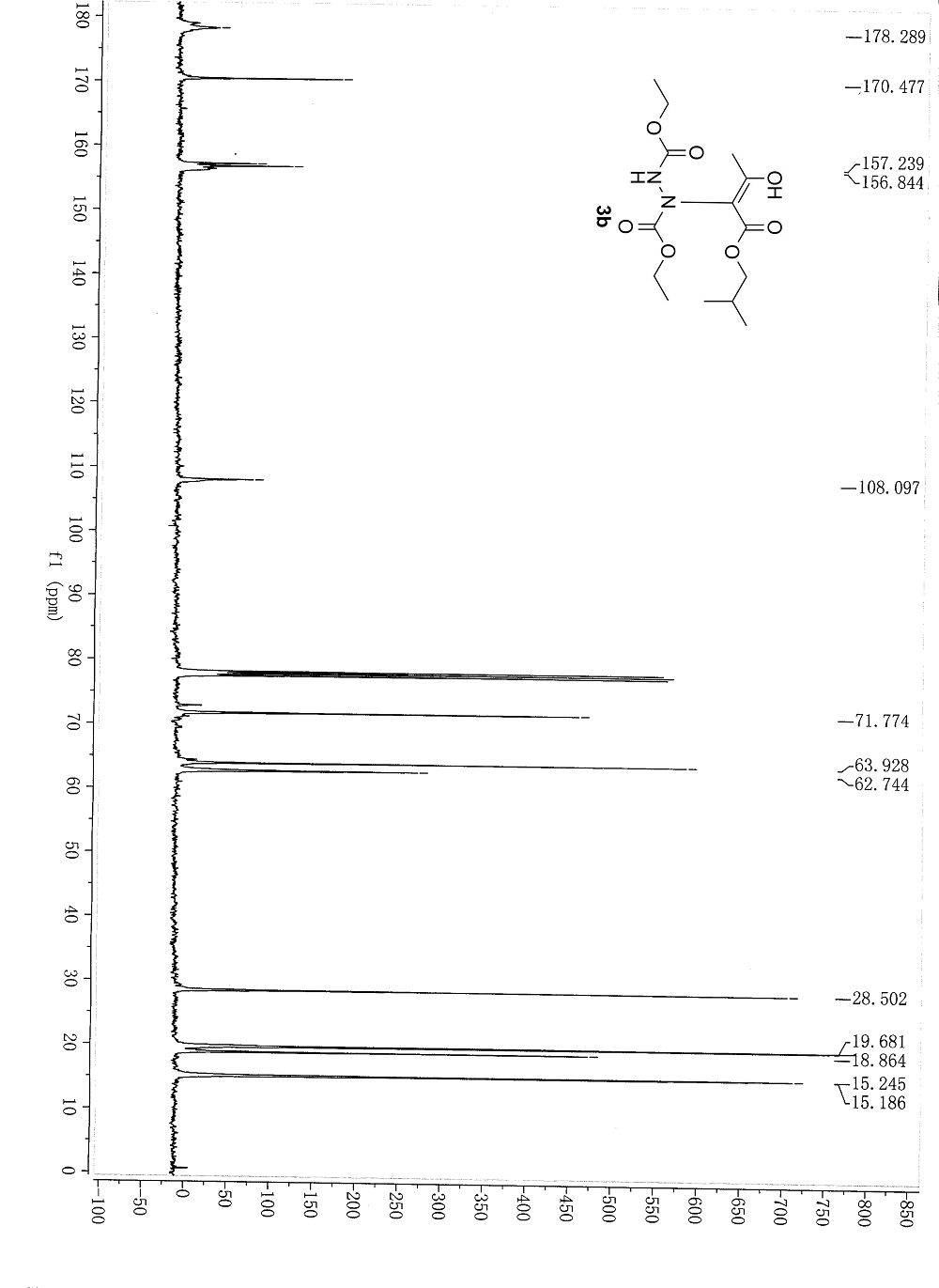


Fig. 25 ^13^C NMR spectrum of product **3b**


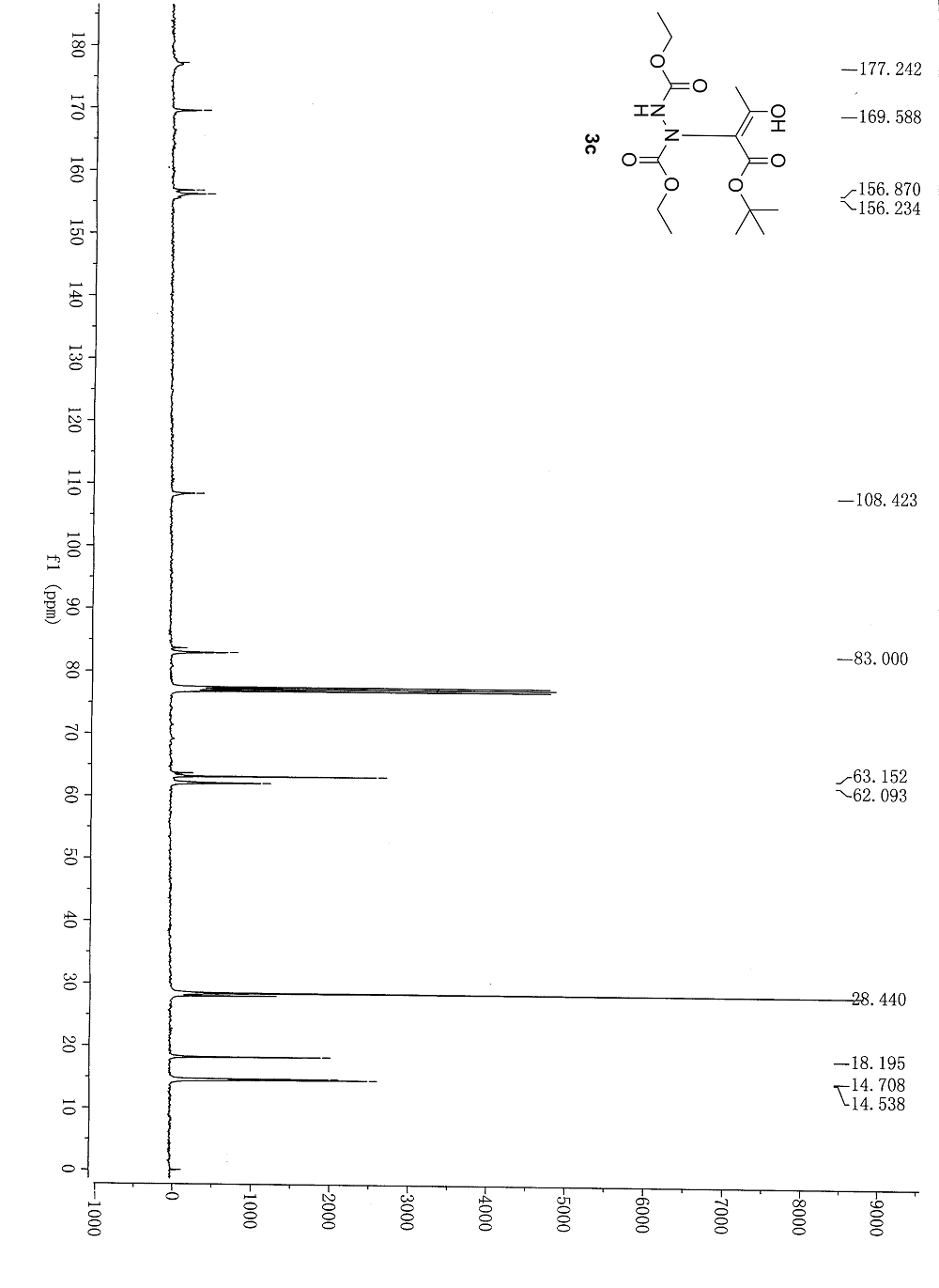


Fig. 26 ^13^C NMR spectrum of product **3c**


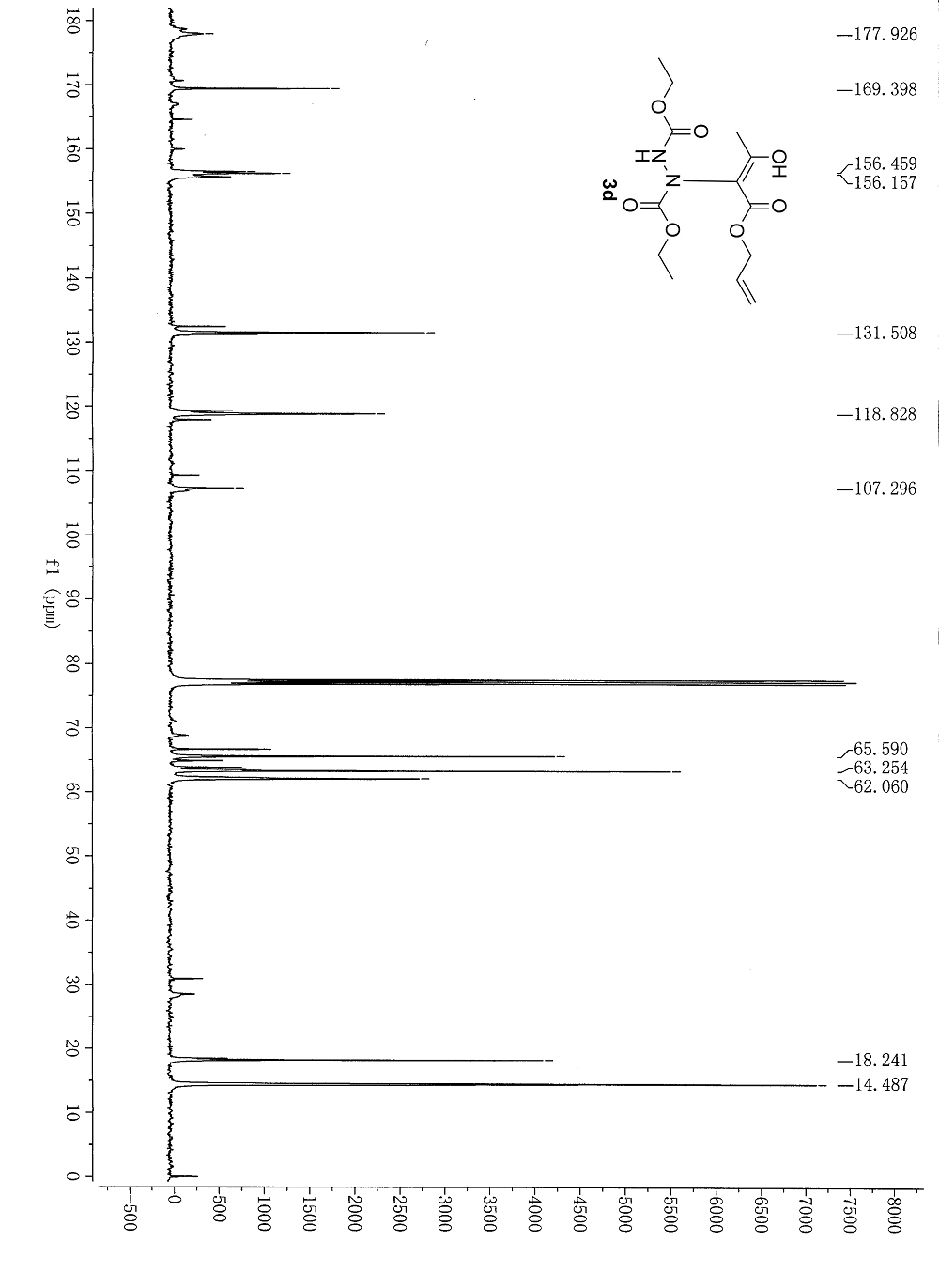


Fig. 27 ^13^C NMR spectrum of product **3d**


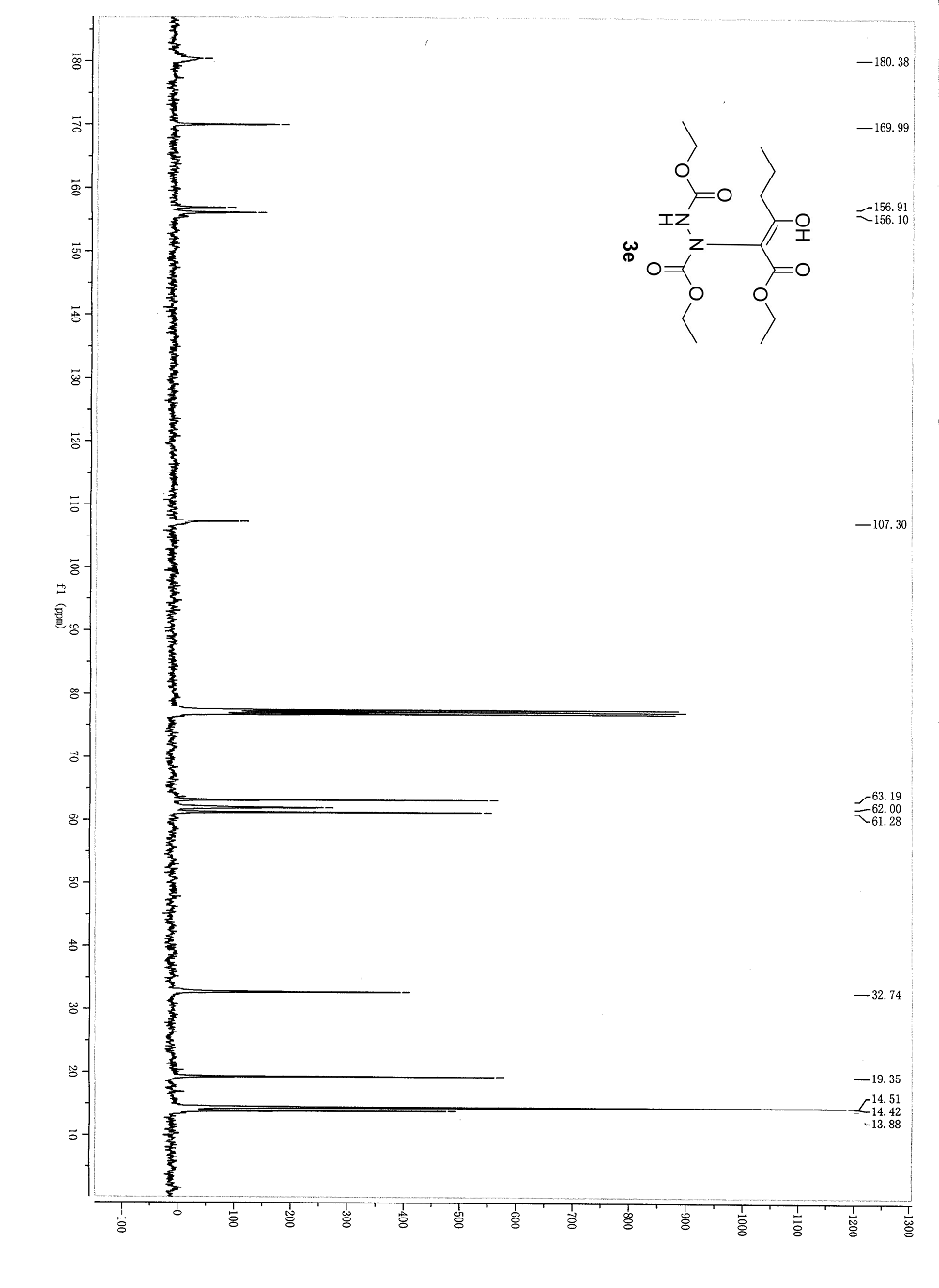


Fig. 28 ^13^C NMR spectrum of product **3e**


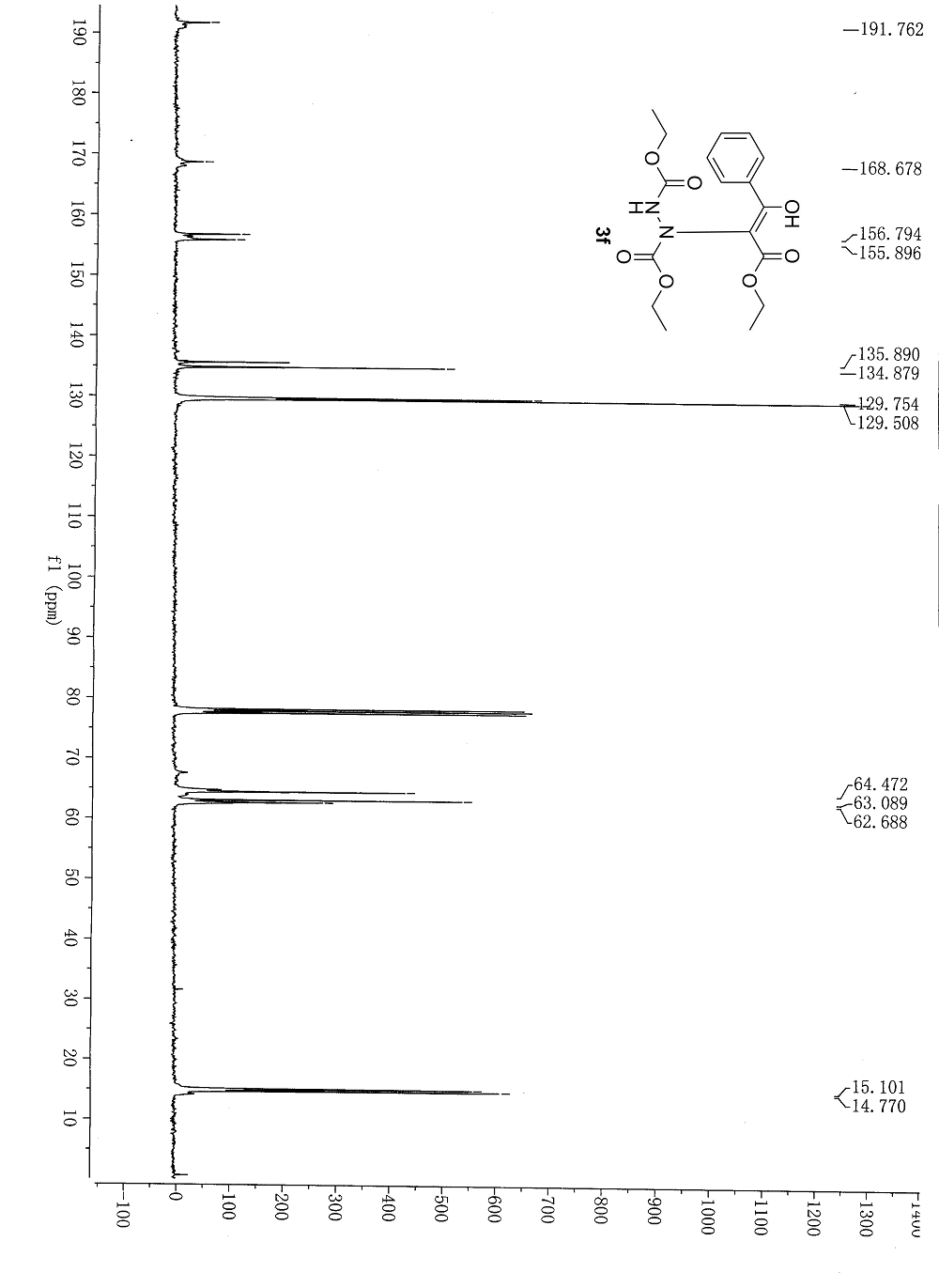


Fig. 29 ^13^C NMR spectrum of product **3f**


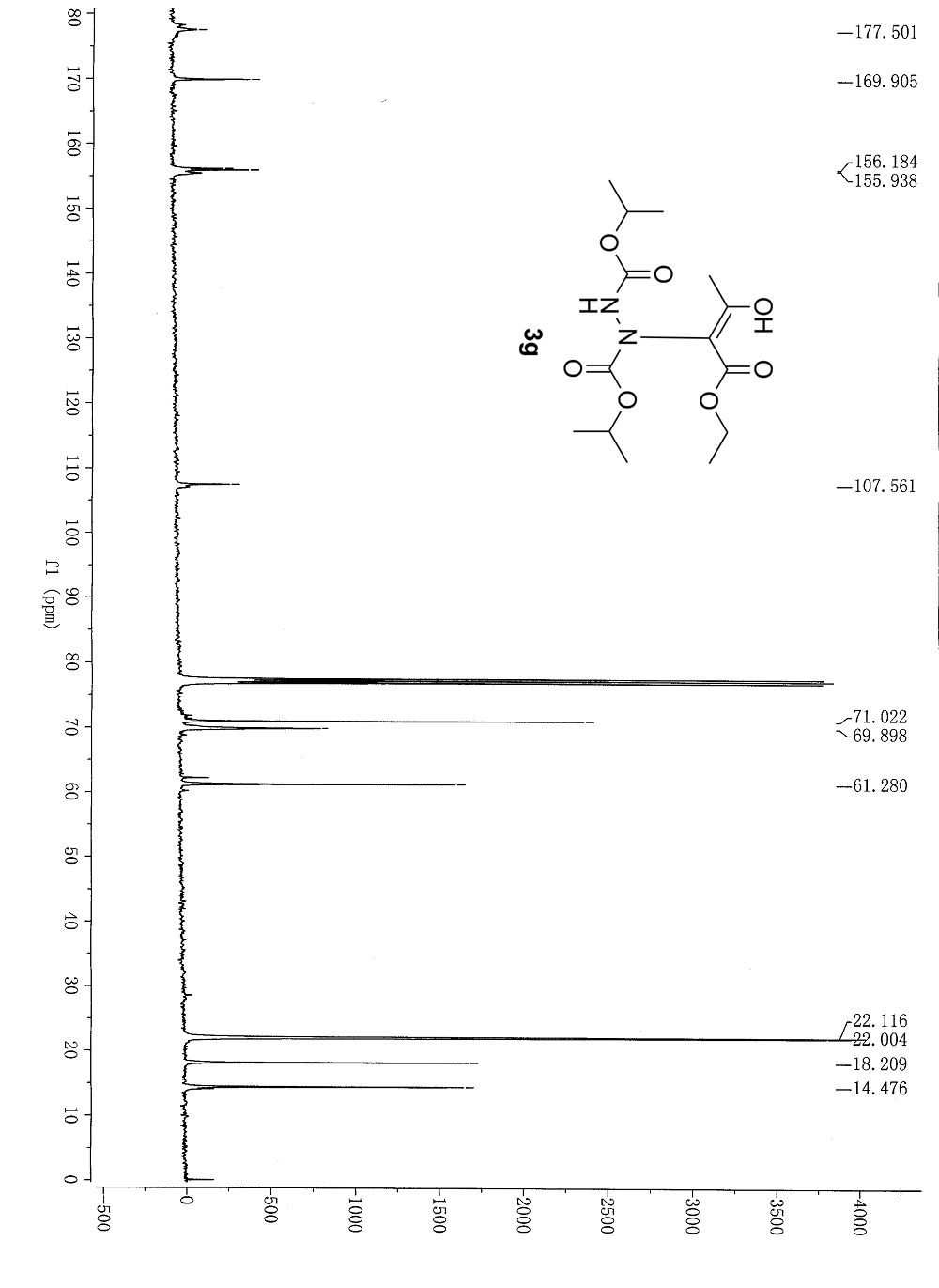


Fig. 30 ^13^C NMR spectrum of product **3g**


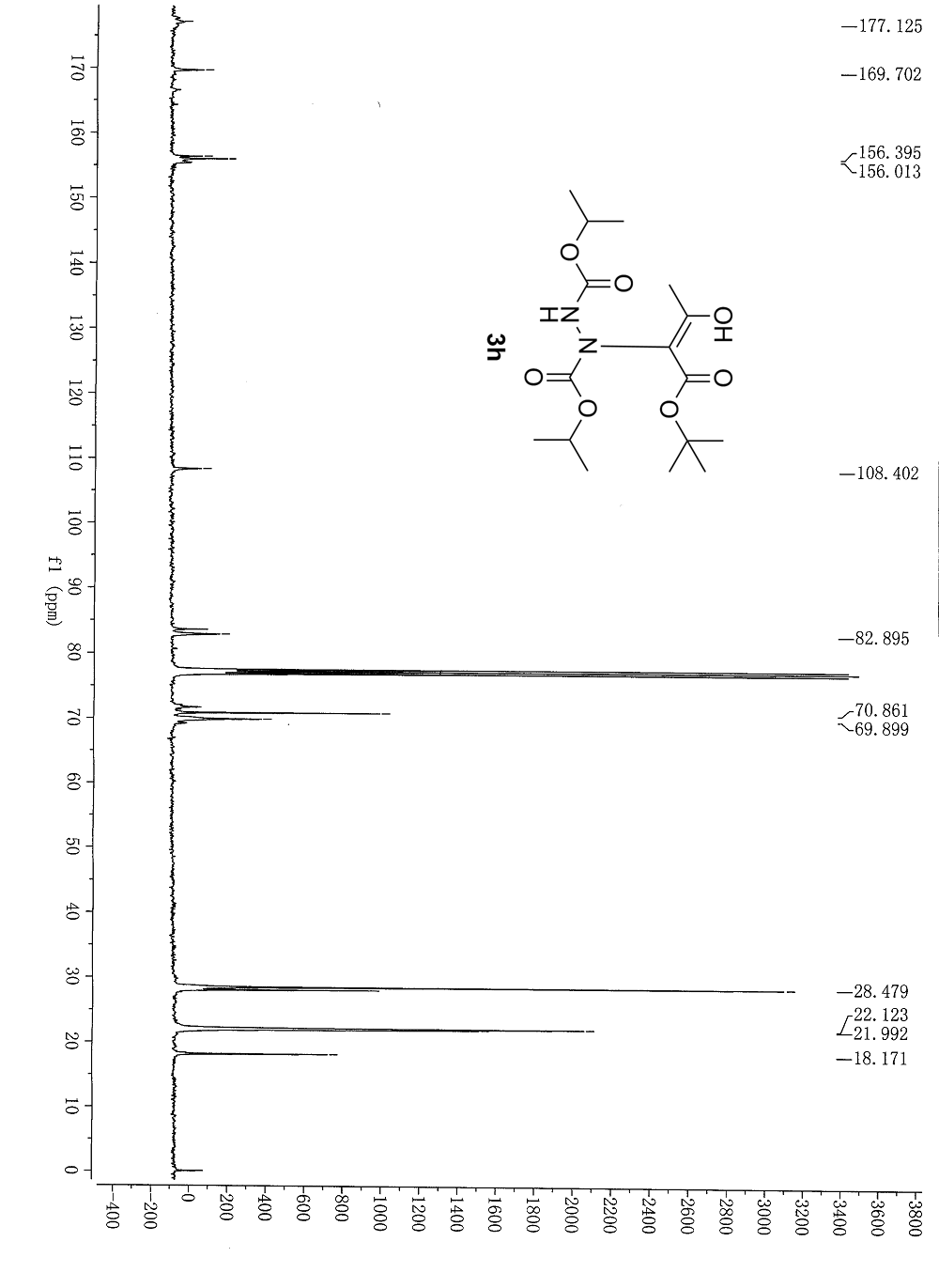


Fig. 31 ^13^C NMR spectrum of product **3h**


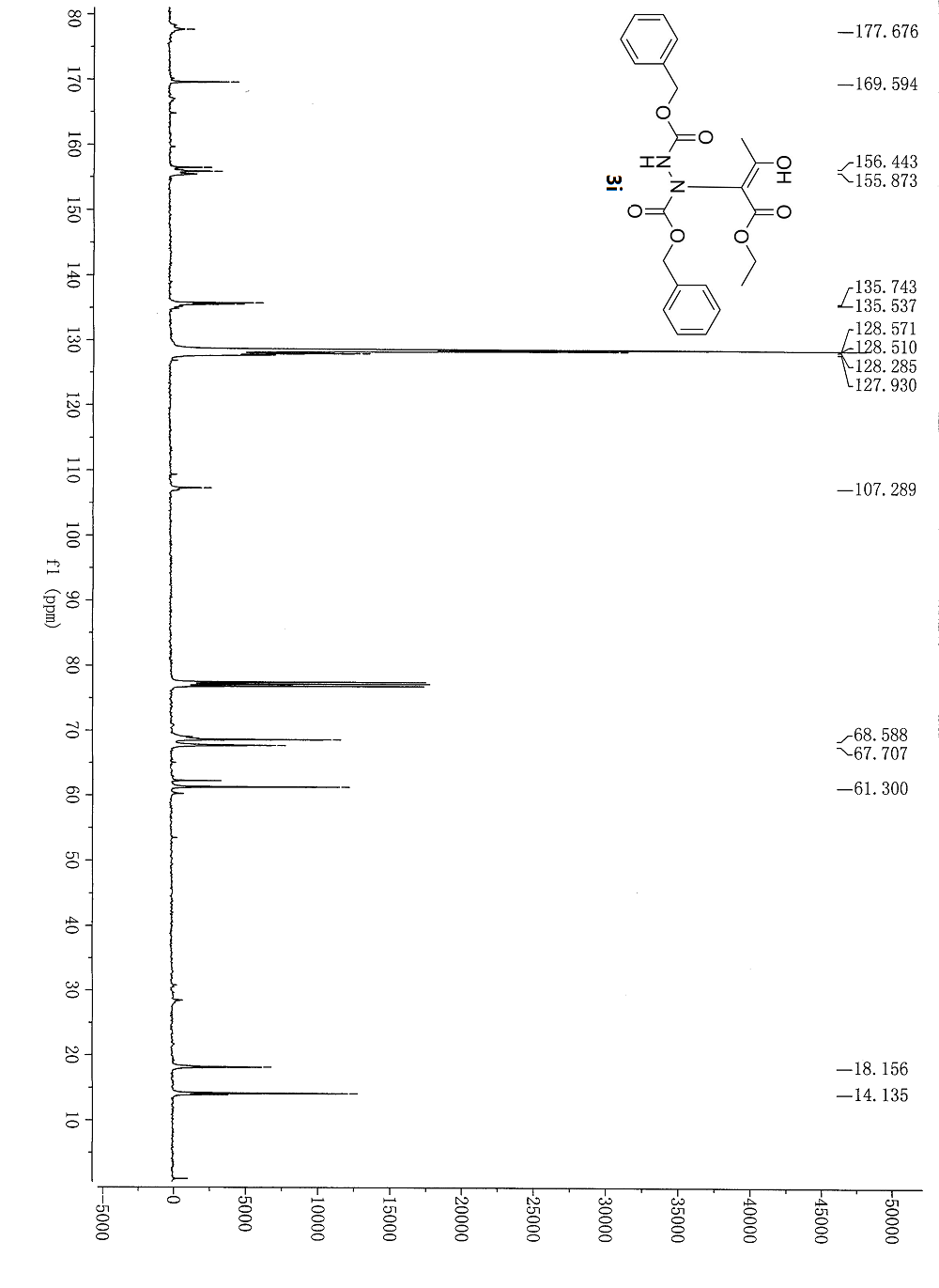


Fig. 32 ^13^C NMR spectrum of product **3i**


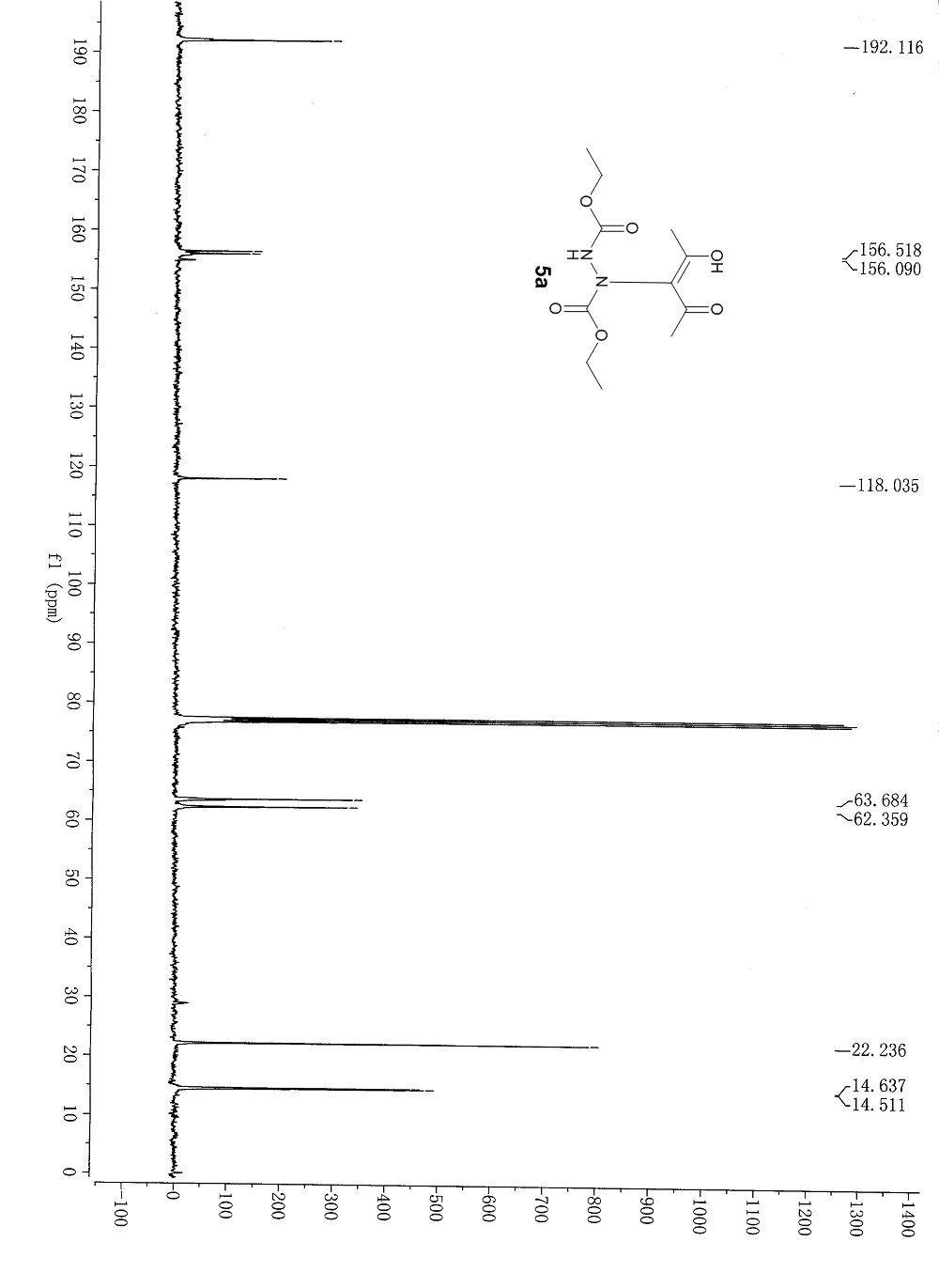


Fig. 33 ^13^C NMR spectrum of product **5a**


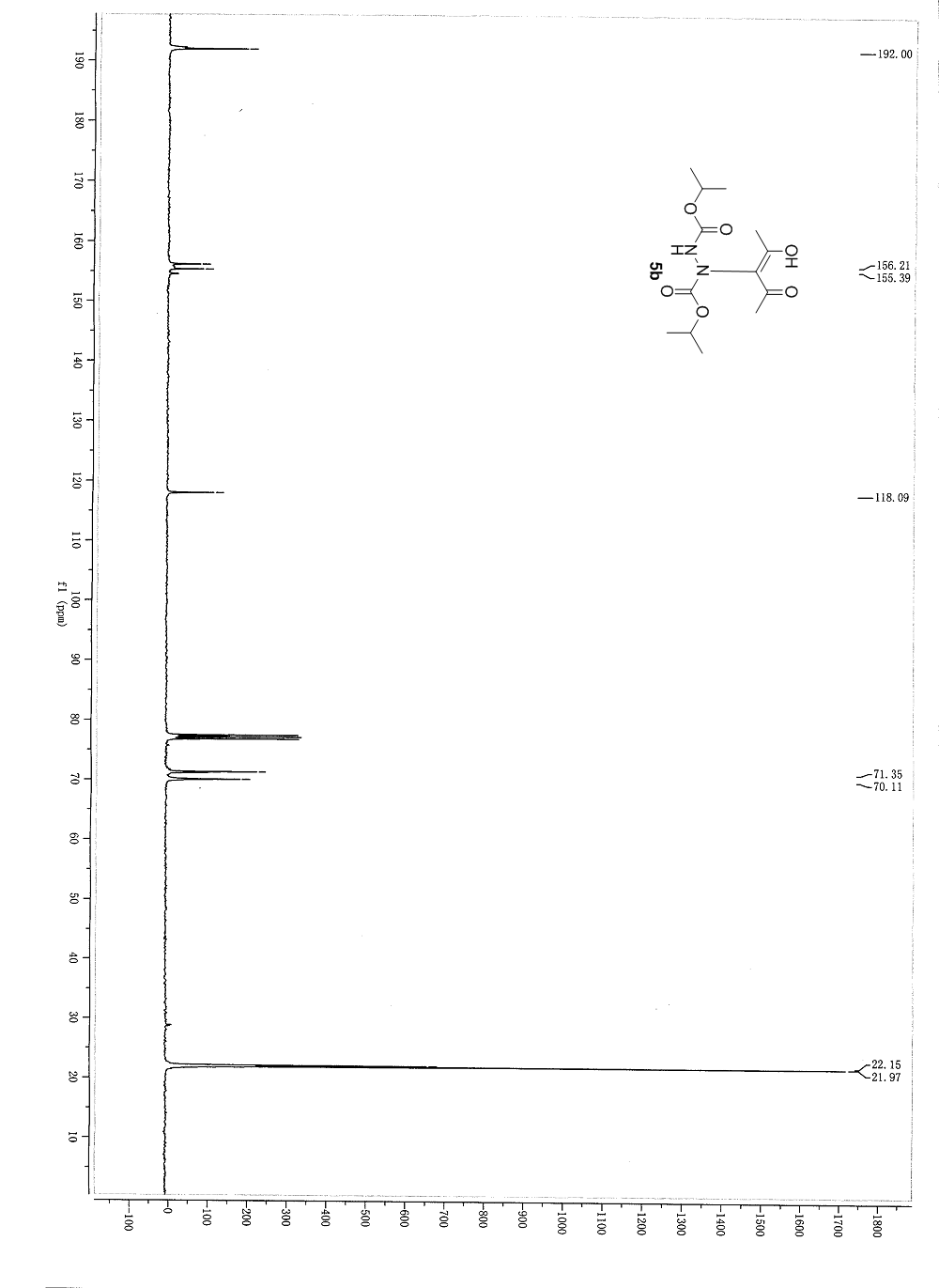


Fig. 34 ^13^C NMR spectrum of product **5b**


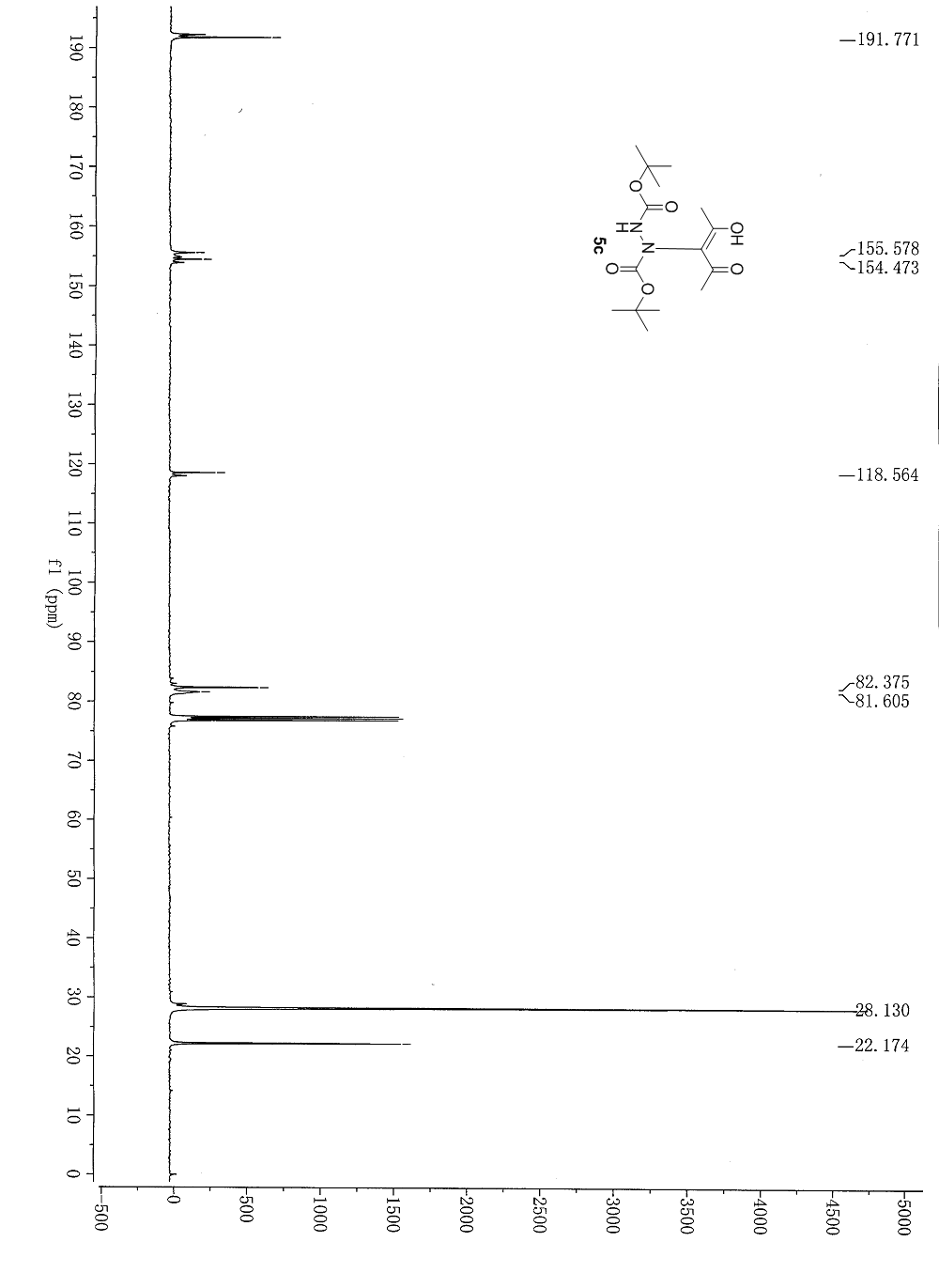


Fig. 35 ^13^C NMR spectrum of product **5c**


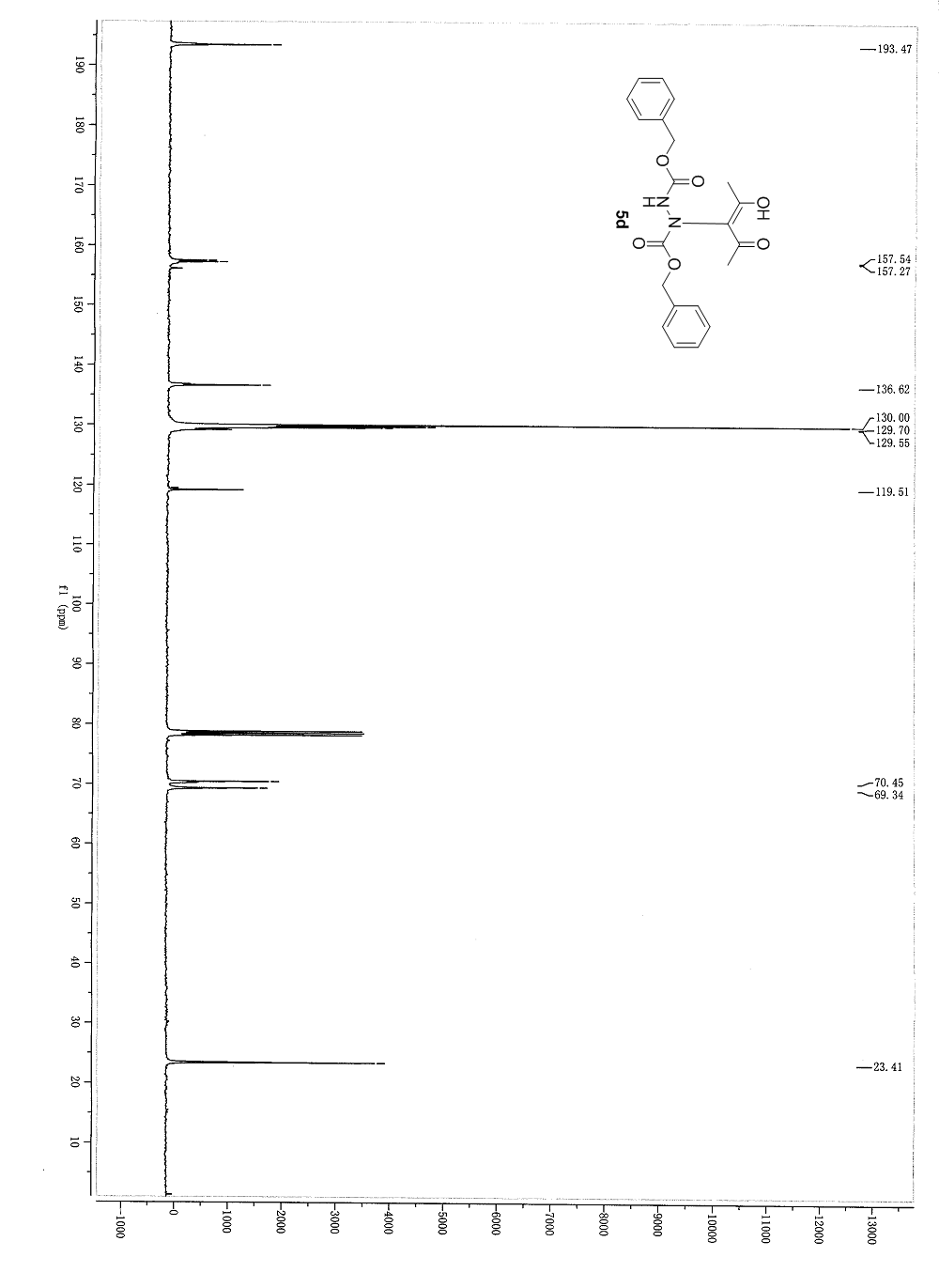


Fig. 36 ^13^C NMR spectrum of product **5d**


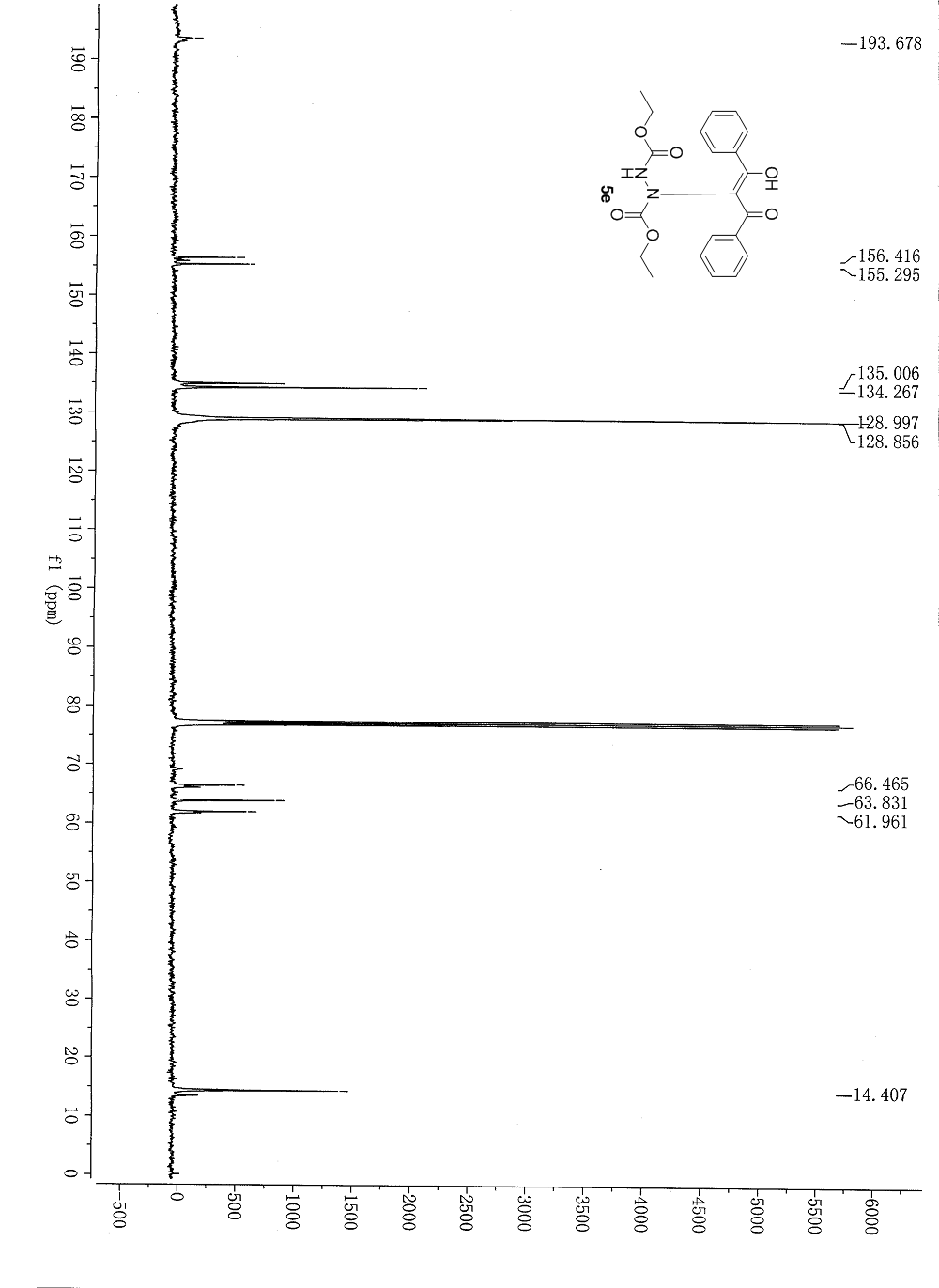


Fig. 37 ^13^C NMR spectrum of product **5e**


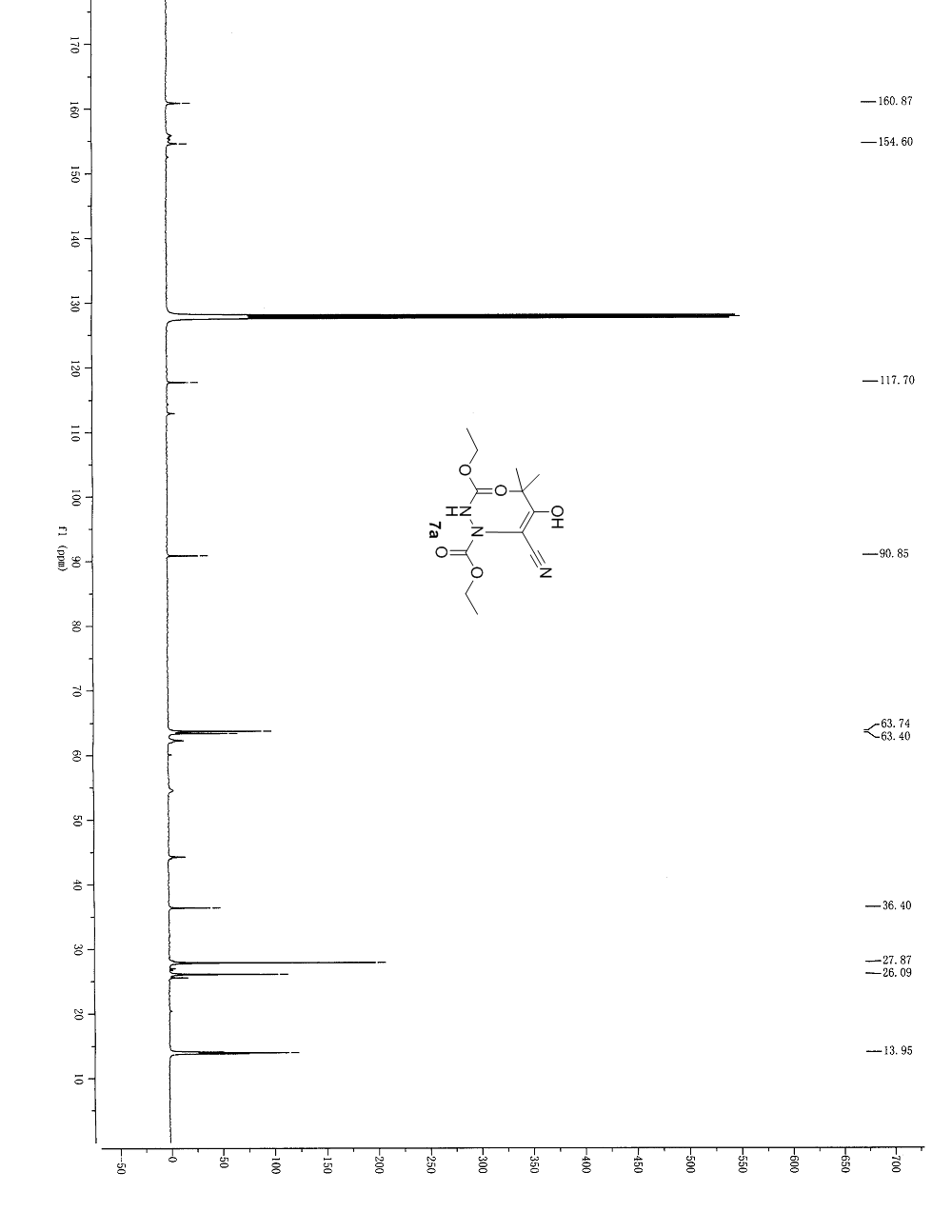


Fig. 38 ^13^C NMR spectrum of product **7a**


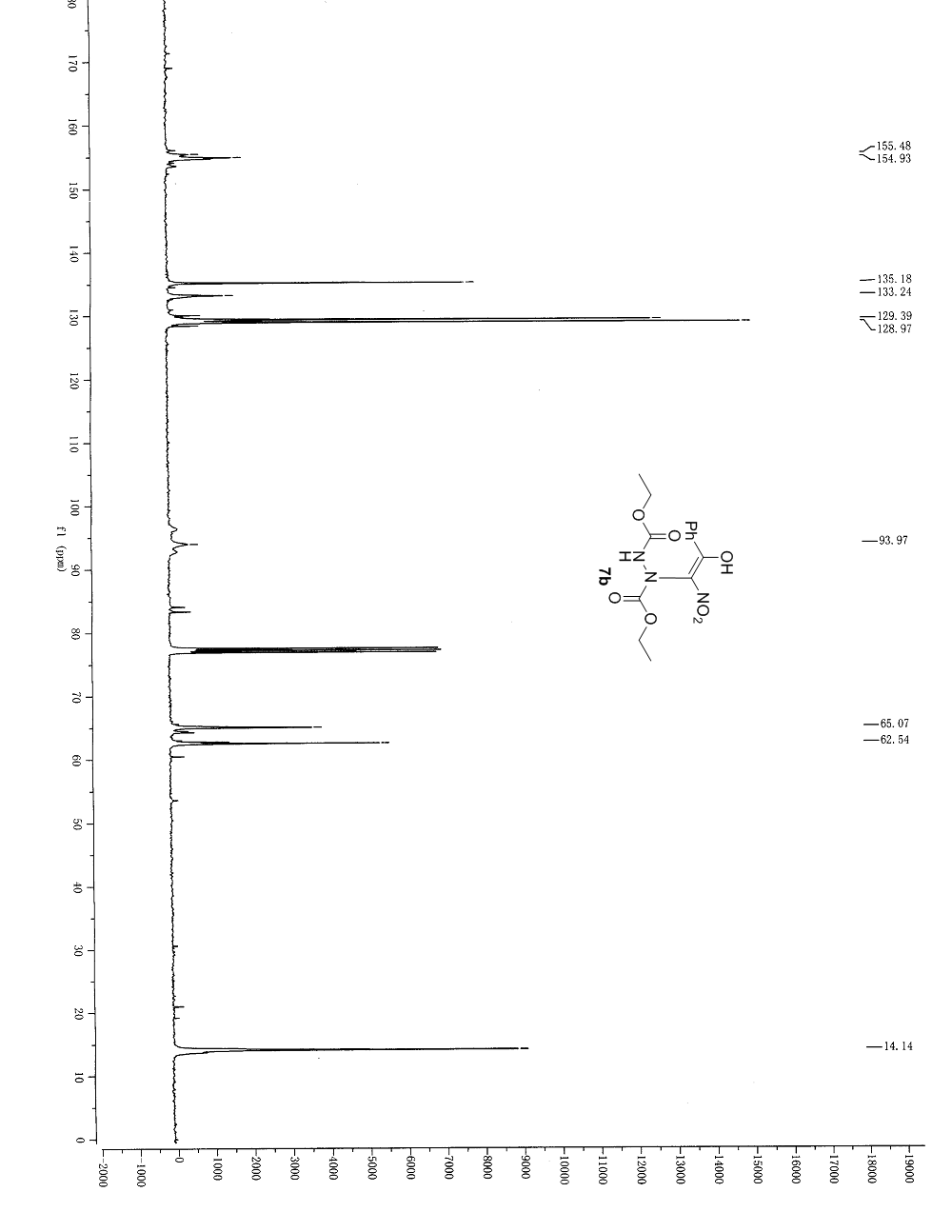


Fig. 39 ^13^C NMR spectrum of product **7b**

**6**. IR Spectra for **3a, 3B, 5a,5b, 7a**

**
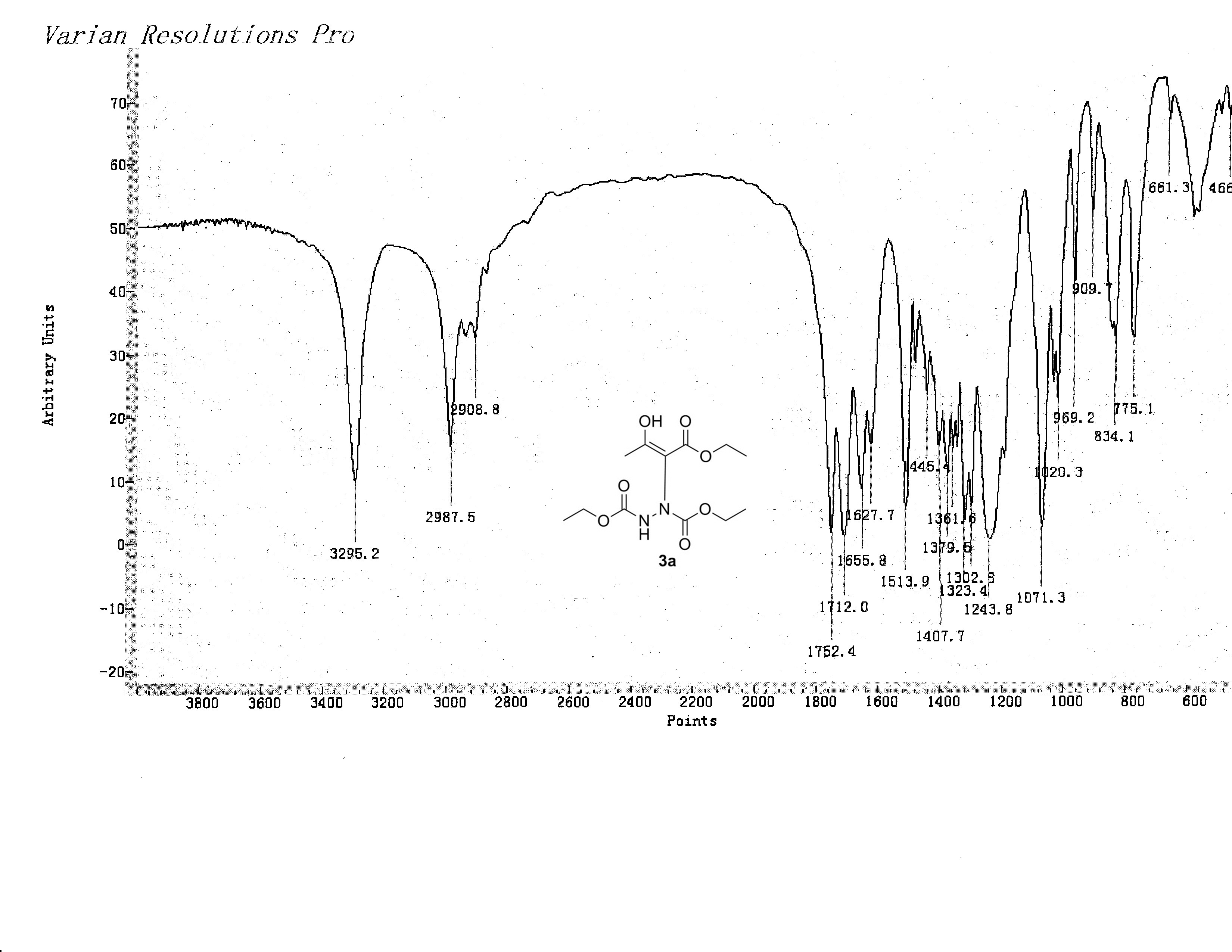
**

Fig. 1 IR Spectra for **3a
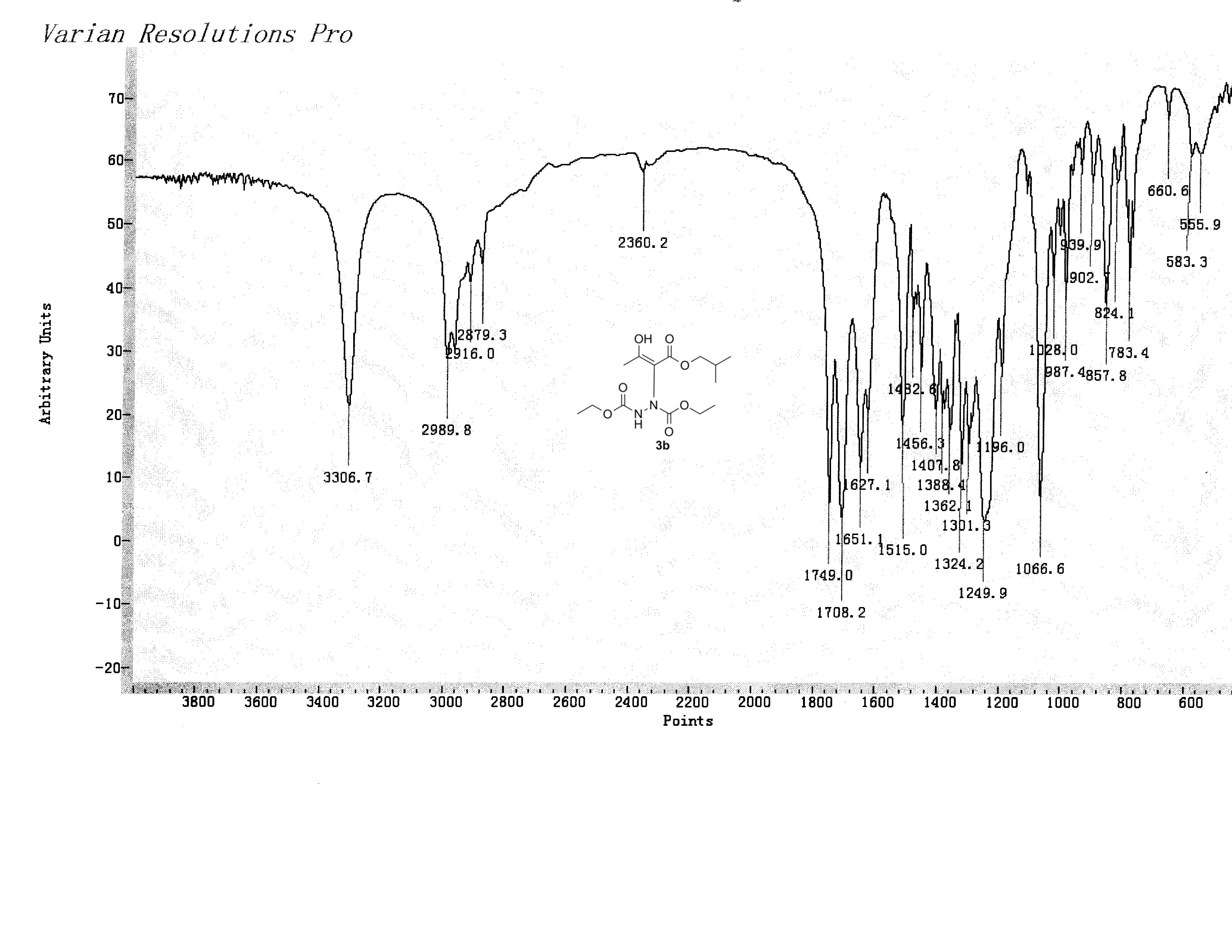
**

Fig. 2 IR Spectra for **3b**


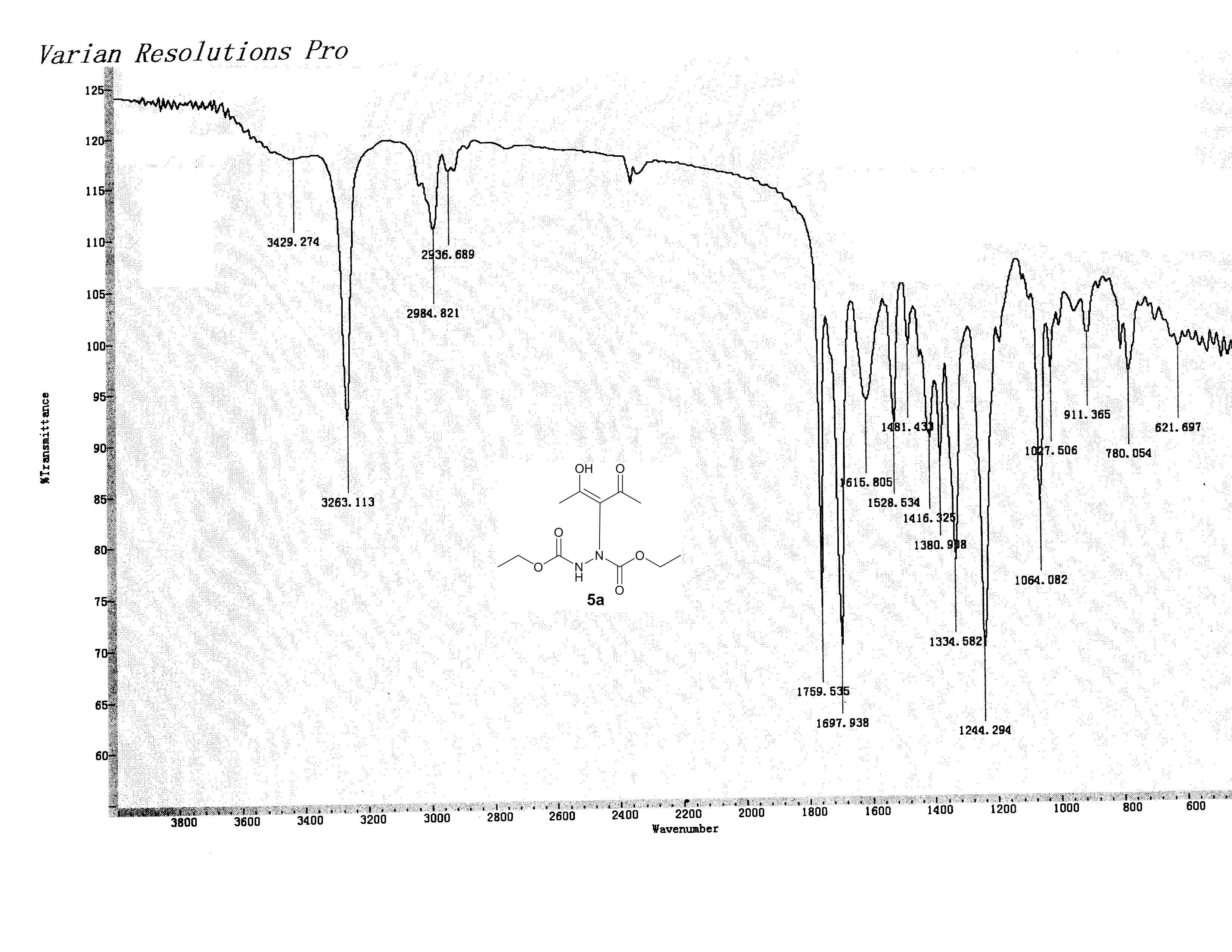


Fig. 3 IR Spectra for **5a**

**
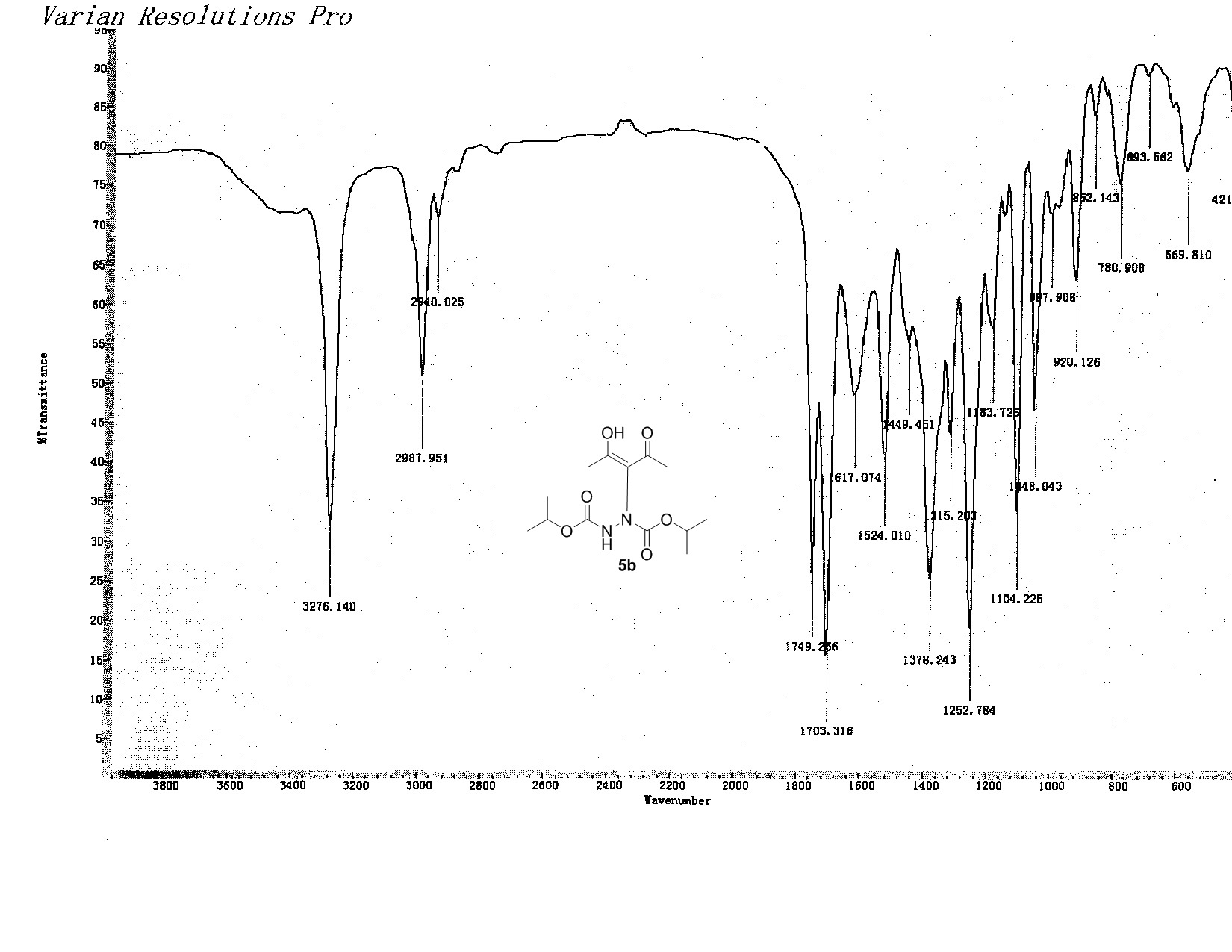
**

Fig. 4 IR Spectra for **5b**

**
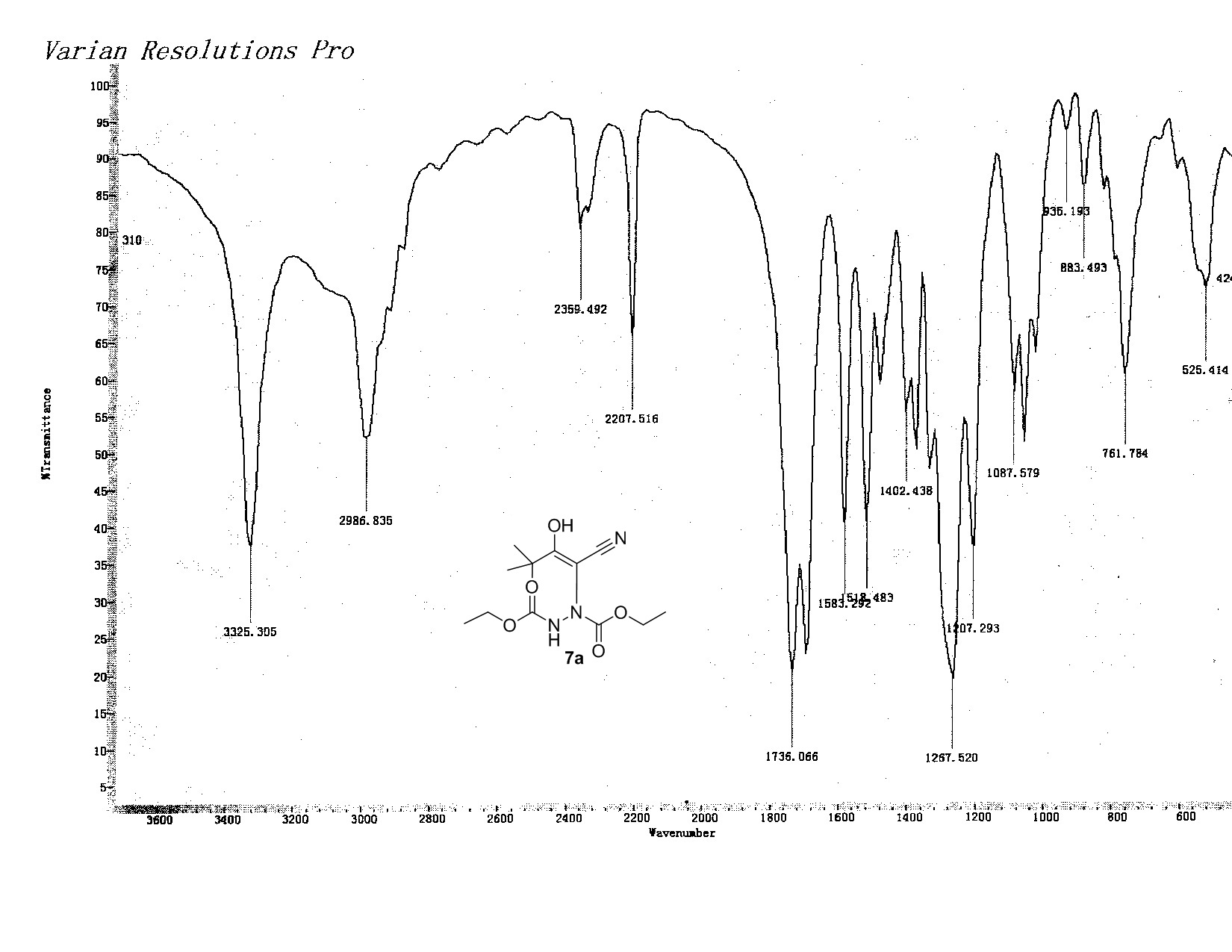
**

Fig. 5 IR Spectra for **7a**

**7**. MS Spectra for **3a-3i, 5a-5e**


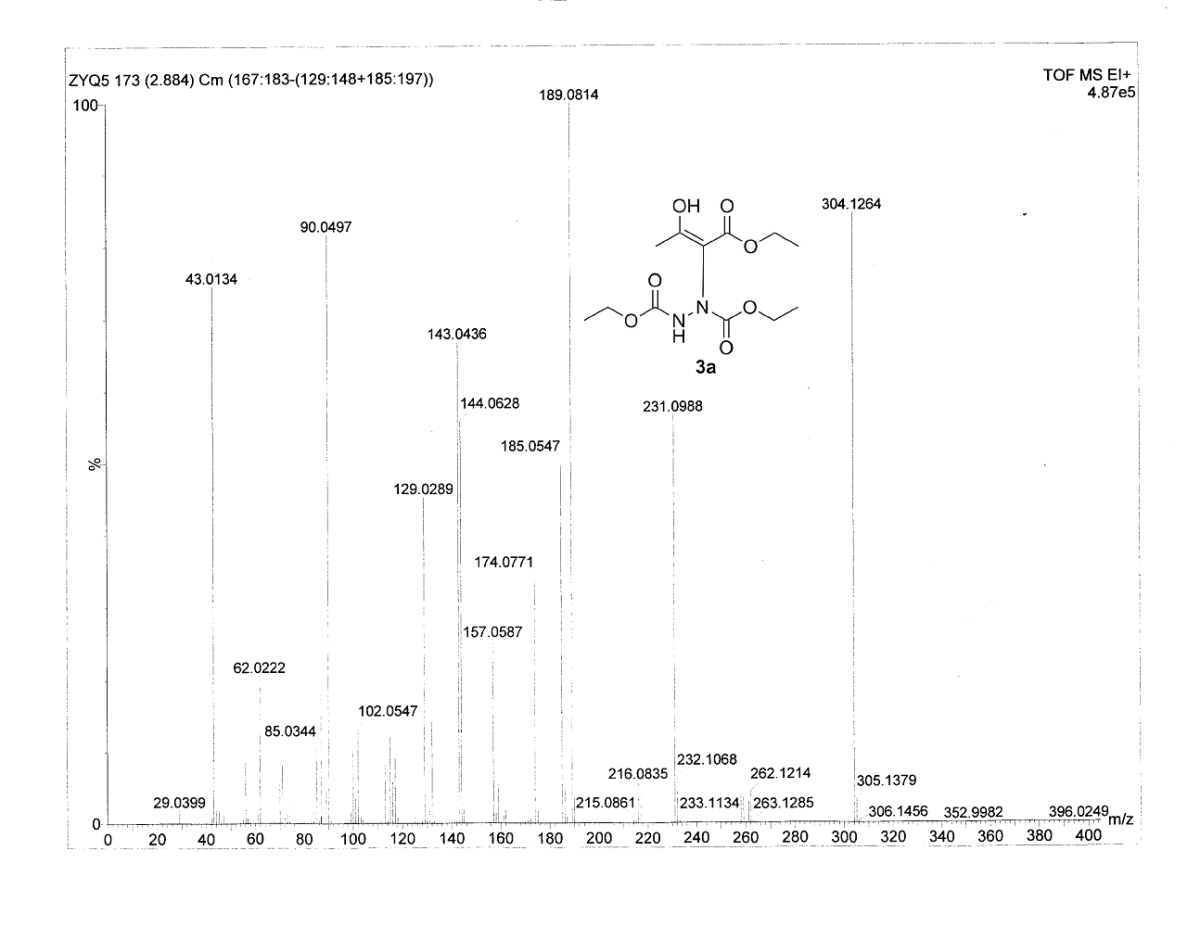


Fig. 40 MS spectrum of product **3a**


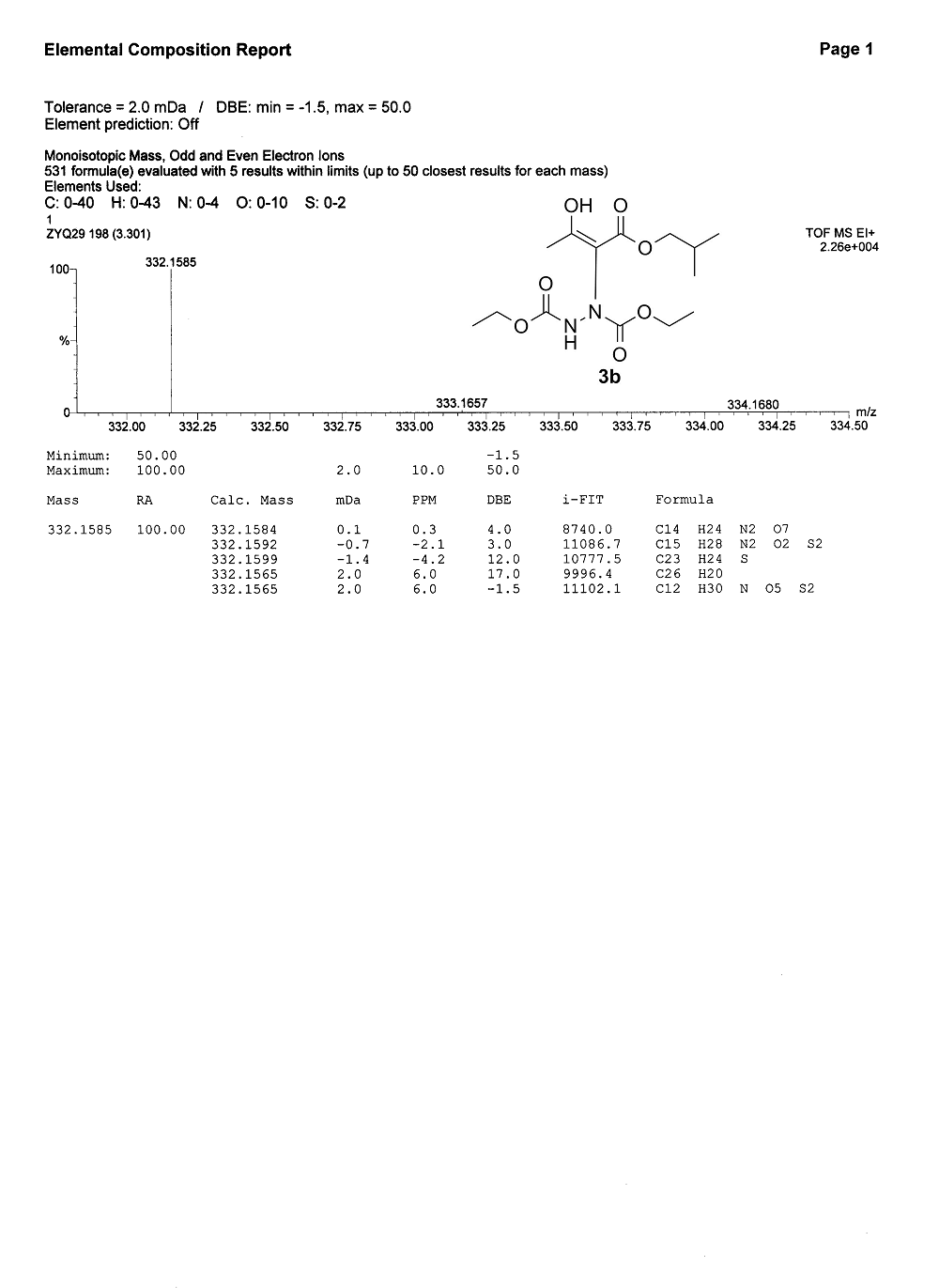


Fig. 41 MS spectrum of product **3b**


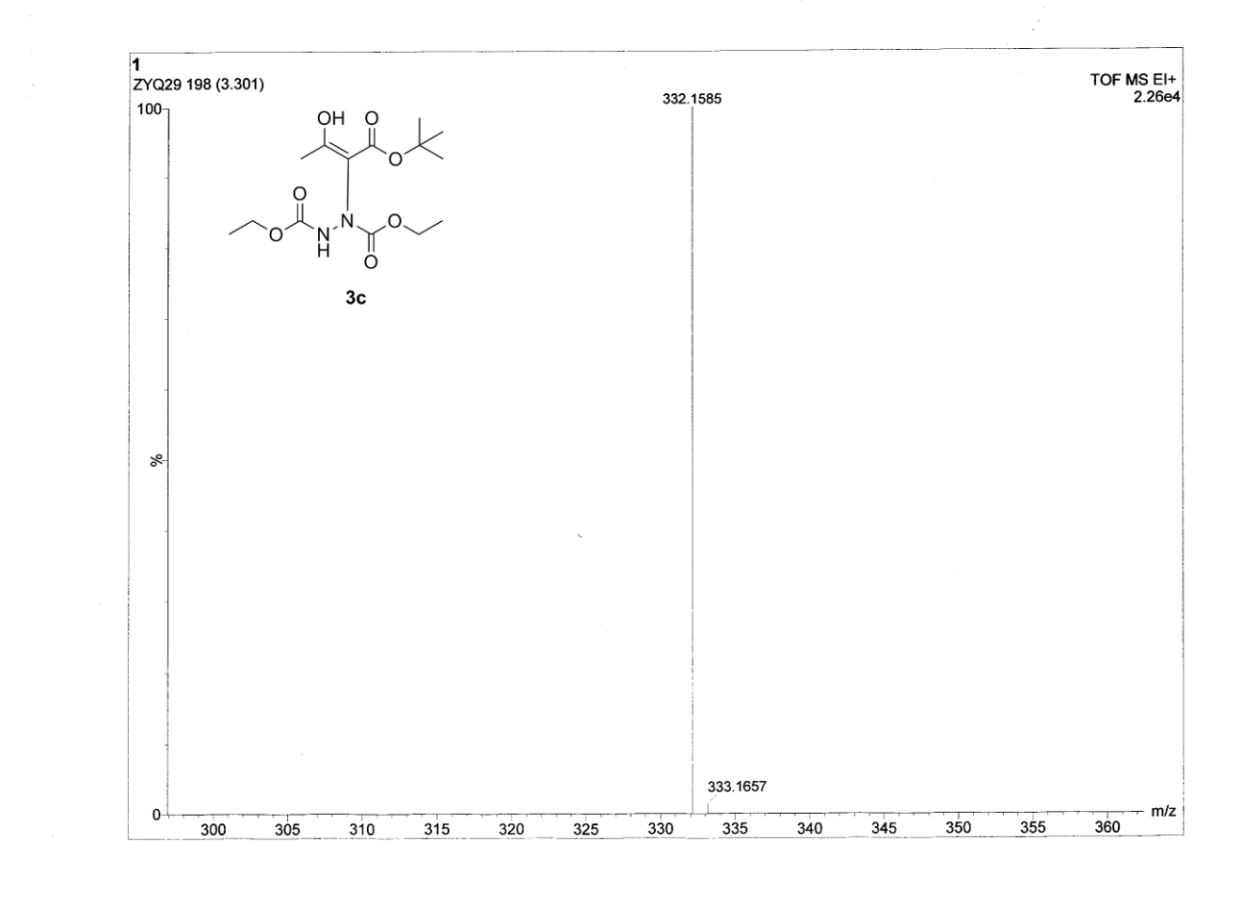


Fig. 42 MS spectrum of product **3c**


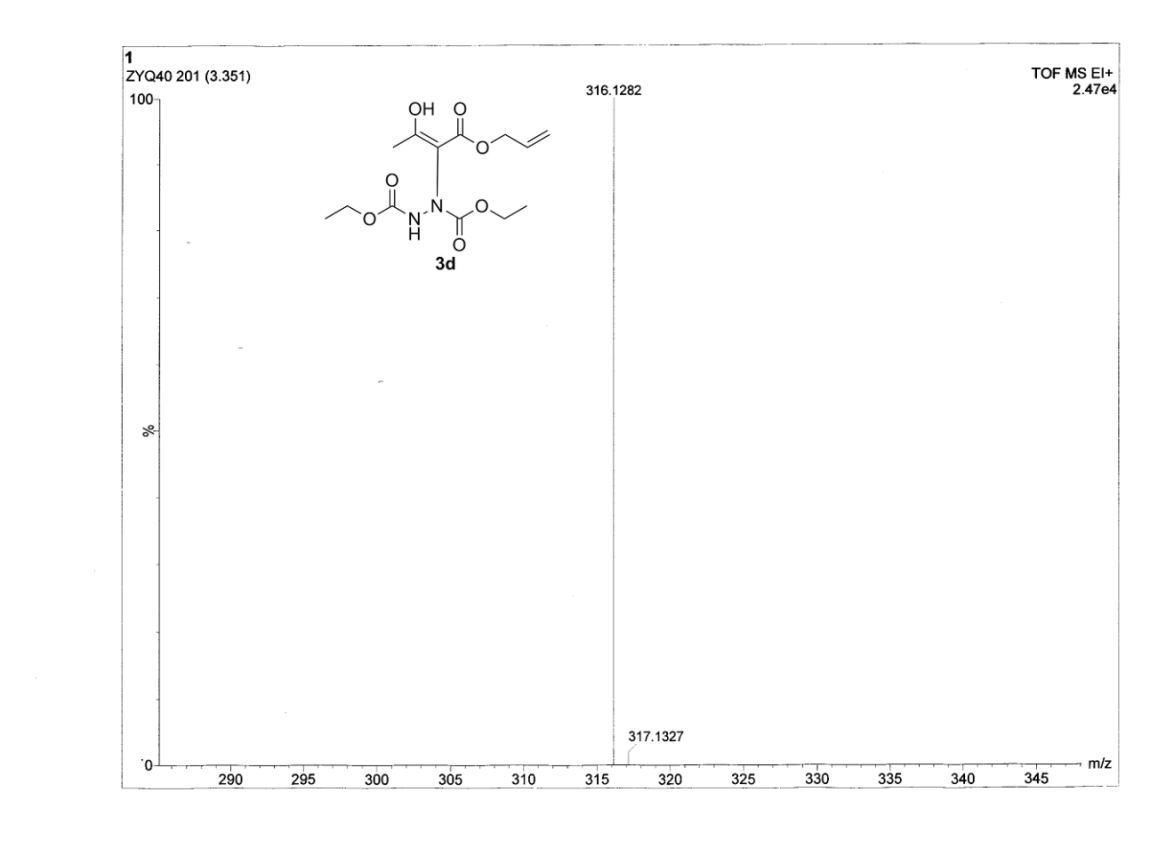


Fig. 43 MS spectrum of product **3d**


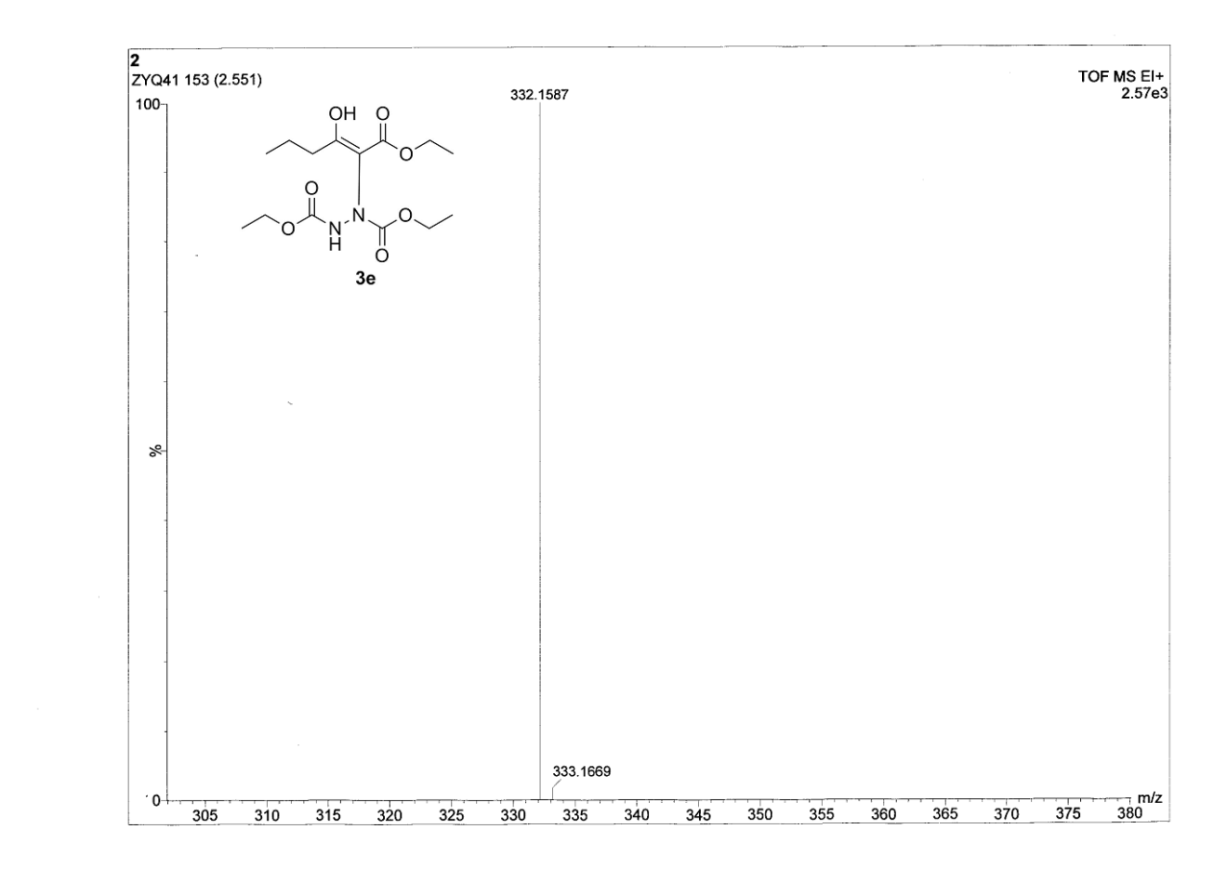


Fig. 44 MS spectrum of product **3e**


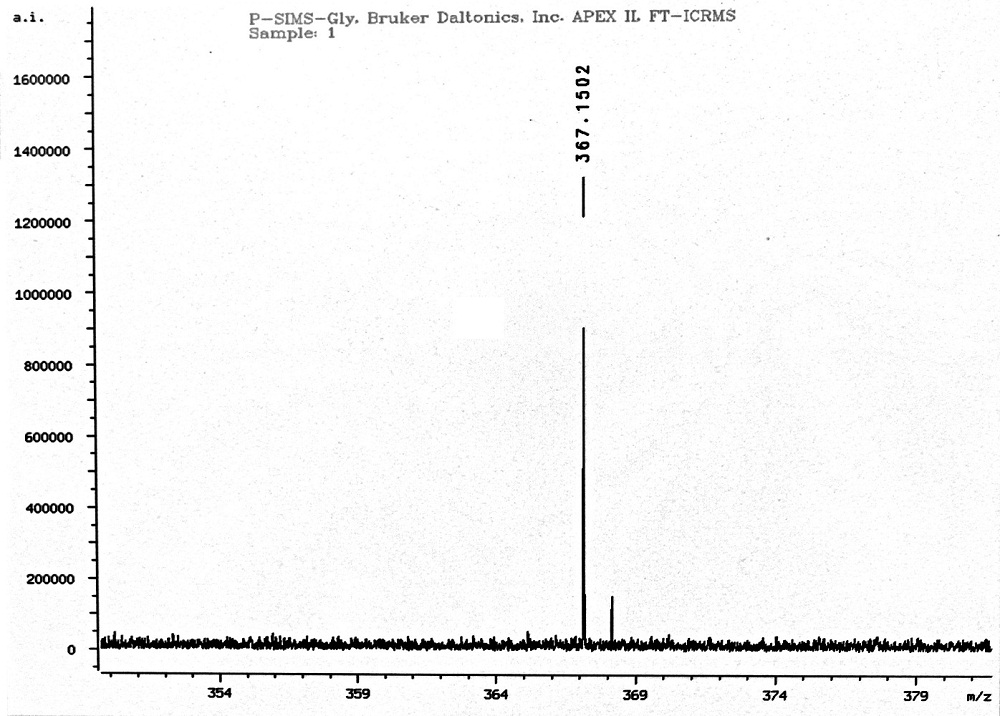


Fig. 45 MS spectrum of product **3f**


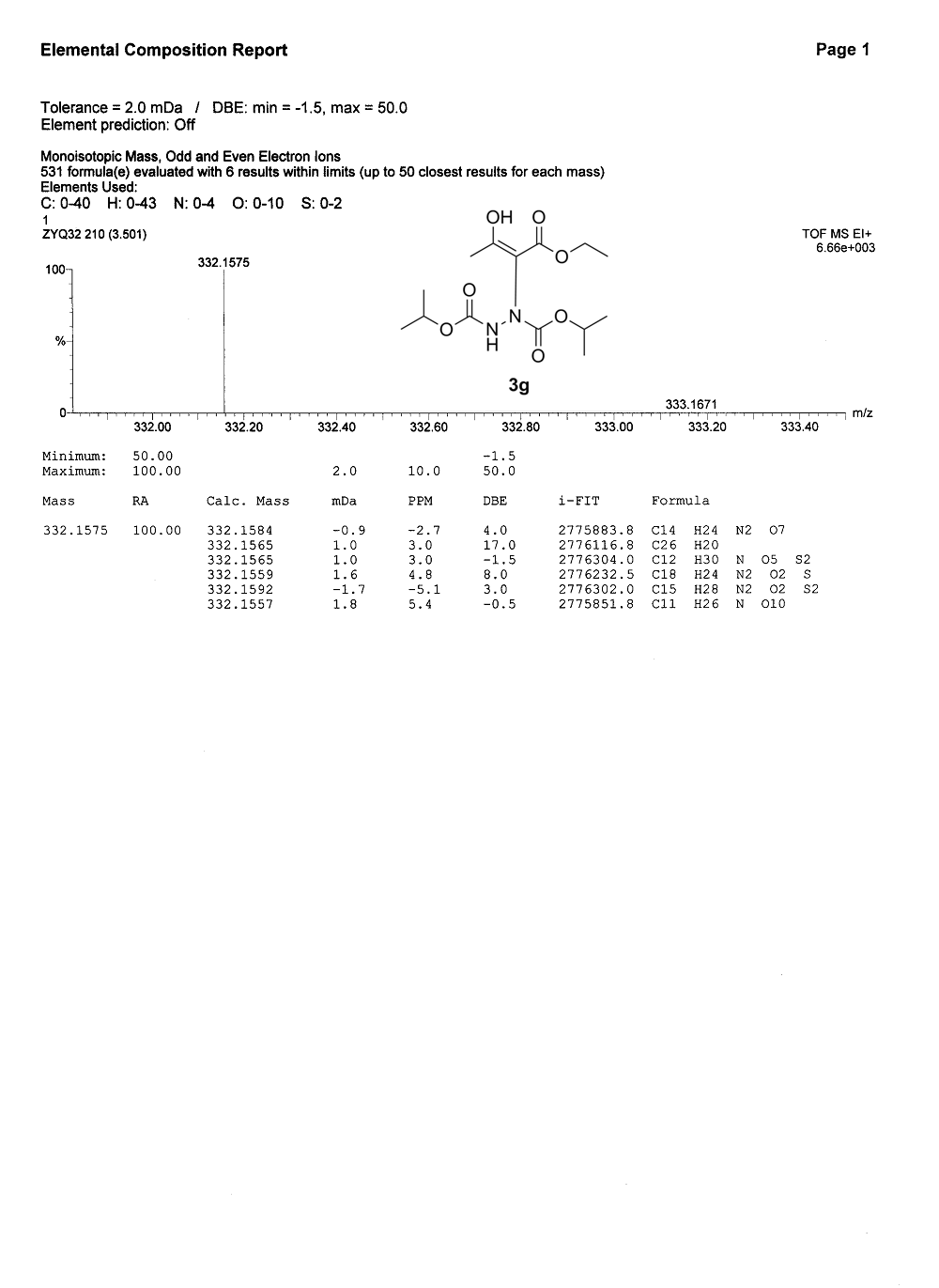


Fig. 46 MS spectrum of product **3g**

**
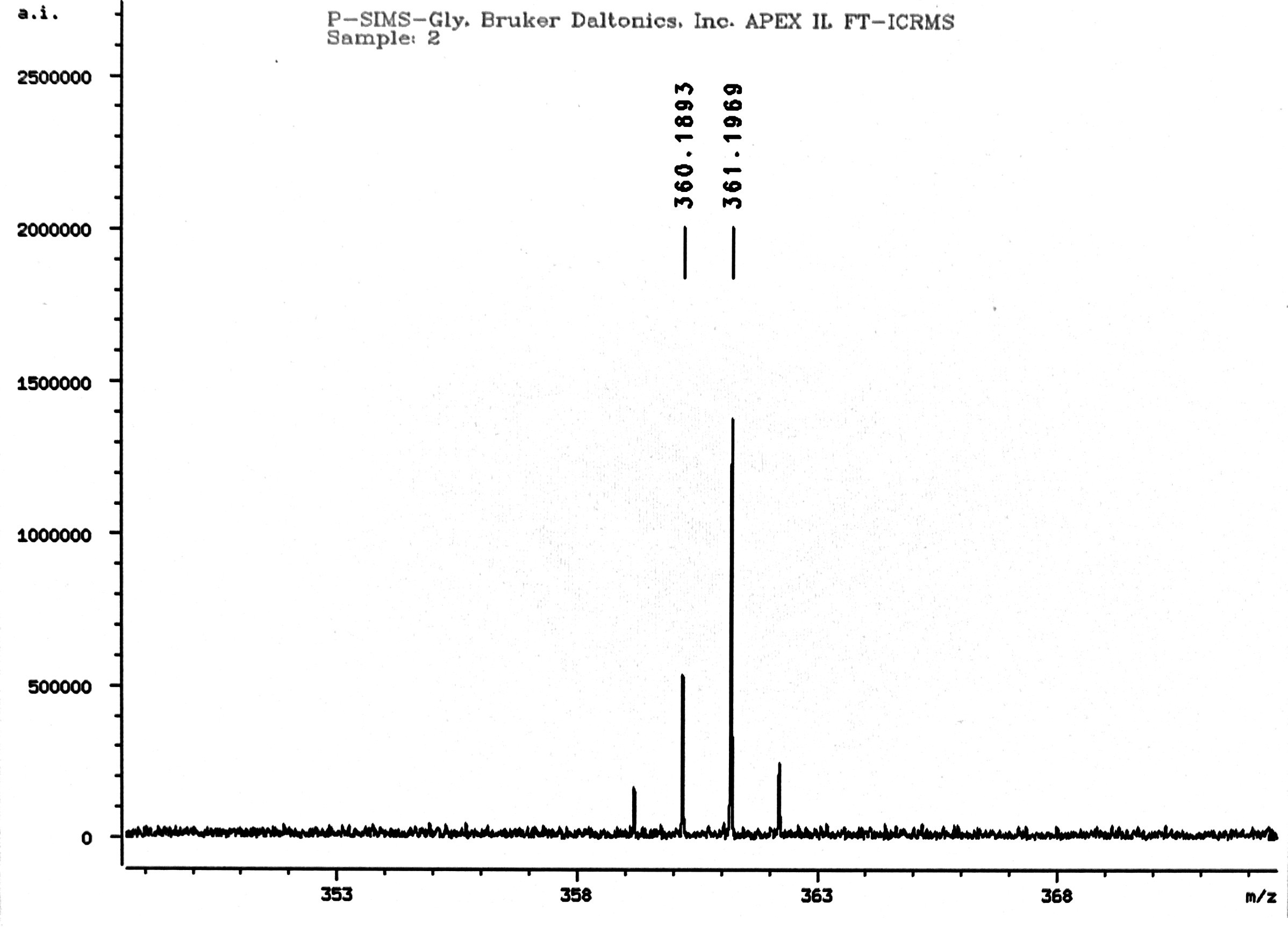
**

Fig. 47 MS spectrum of product **3h**

**
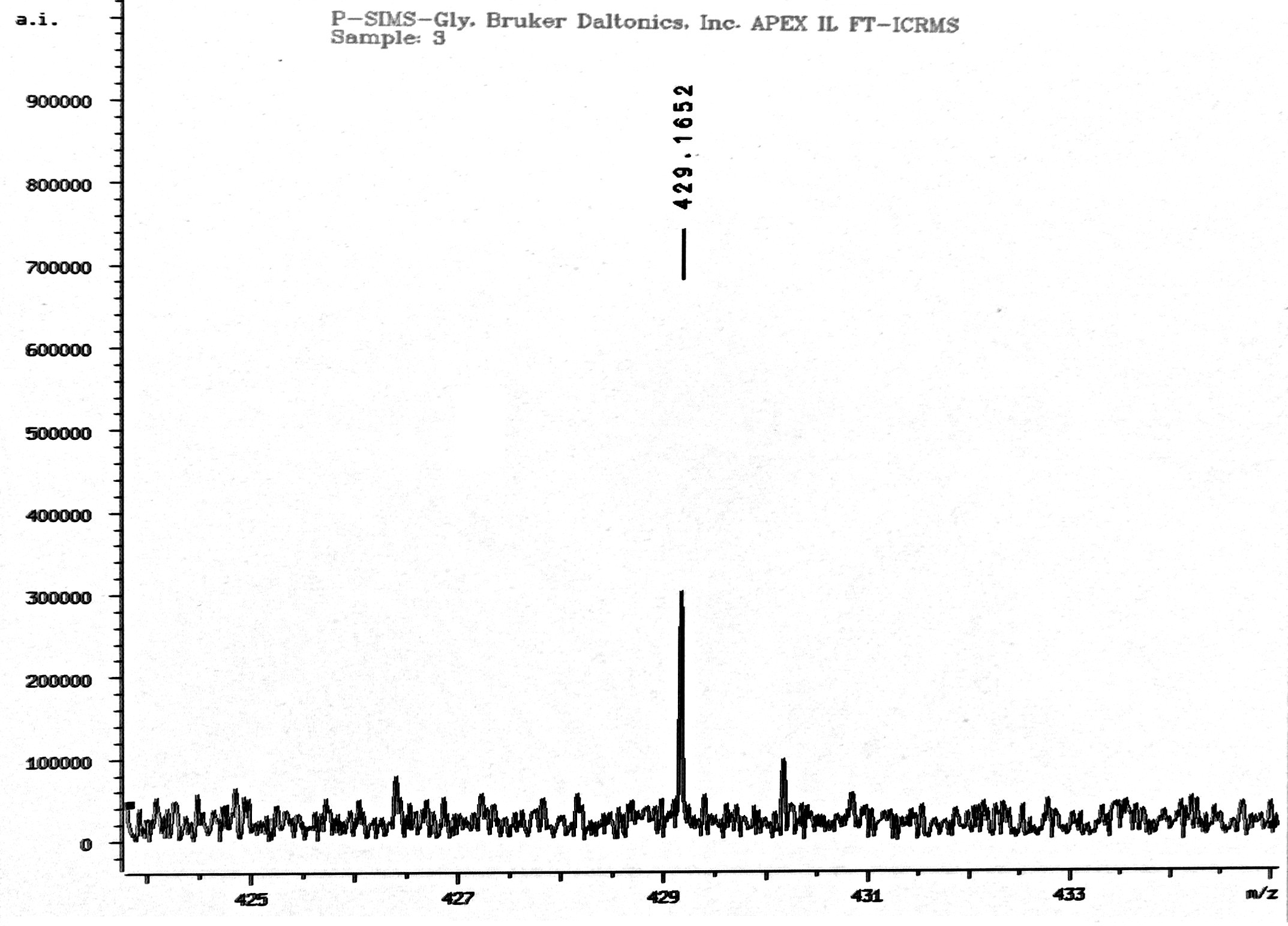
**

Fig. 48 MS spectrum of product **3i**


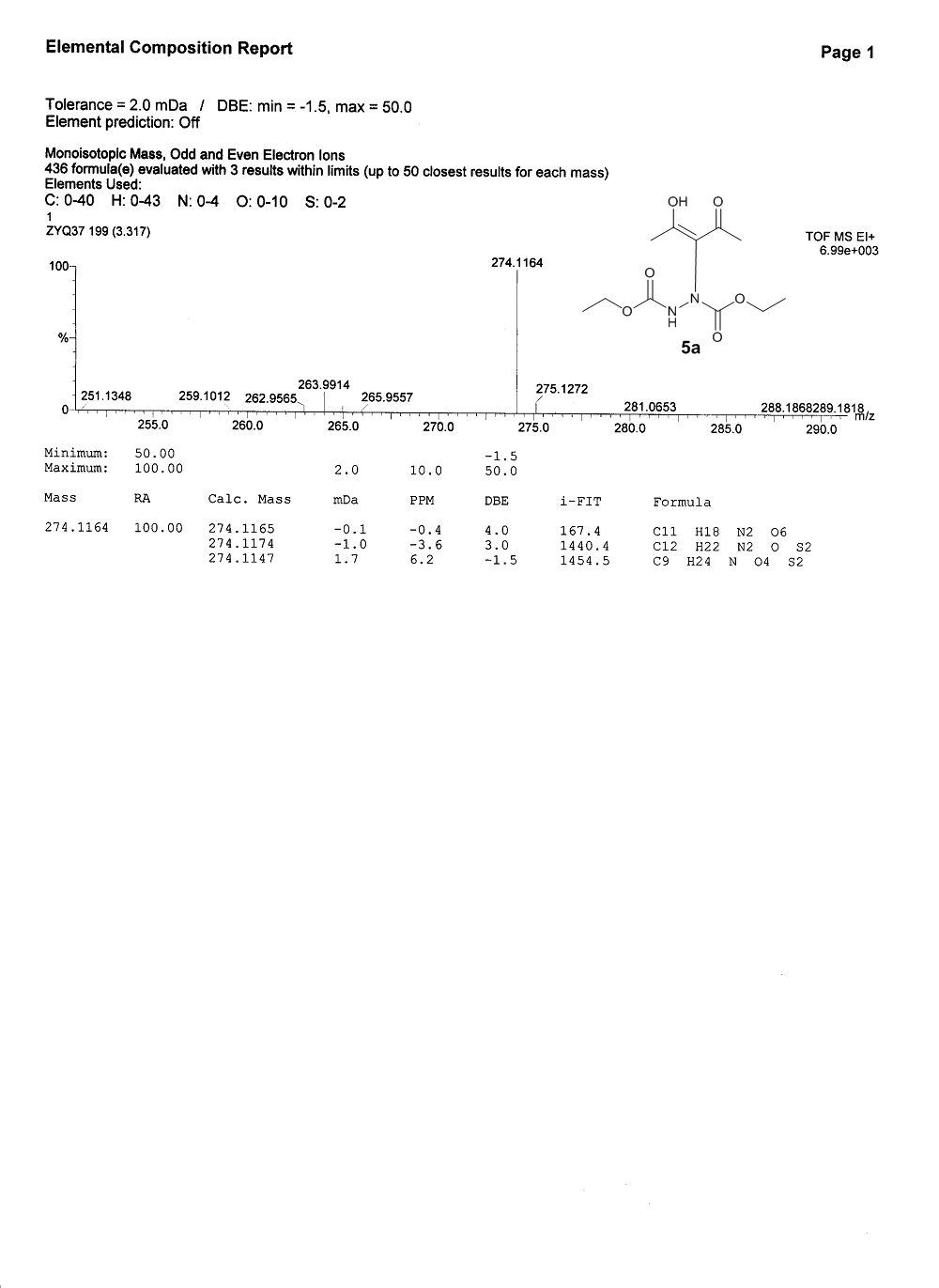


Fig. 49 MS spectrum of product **5a**


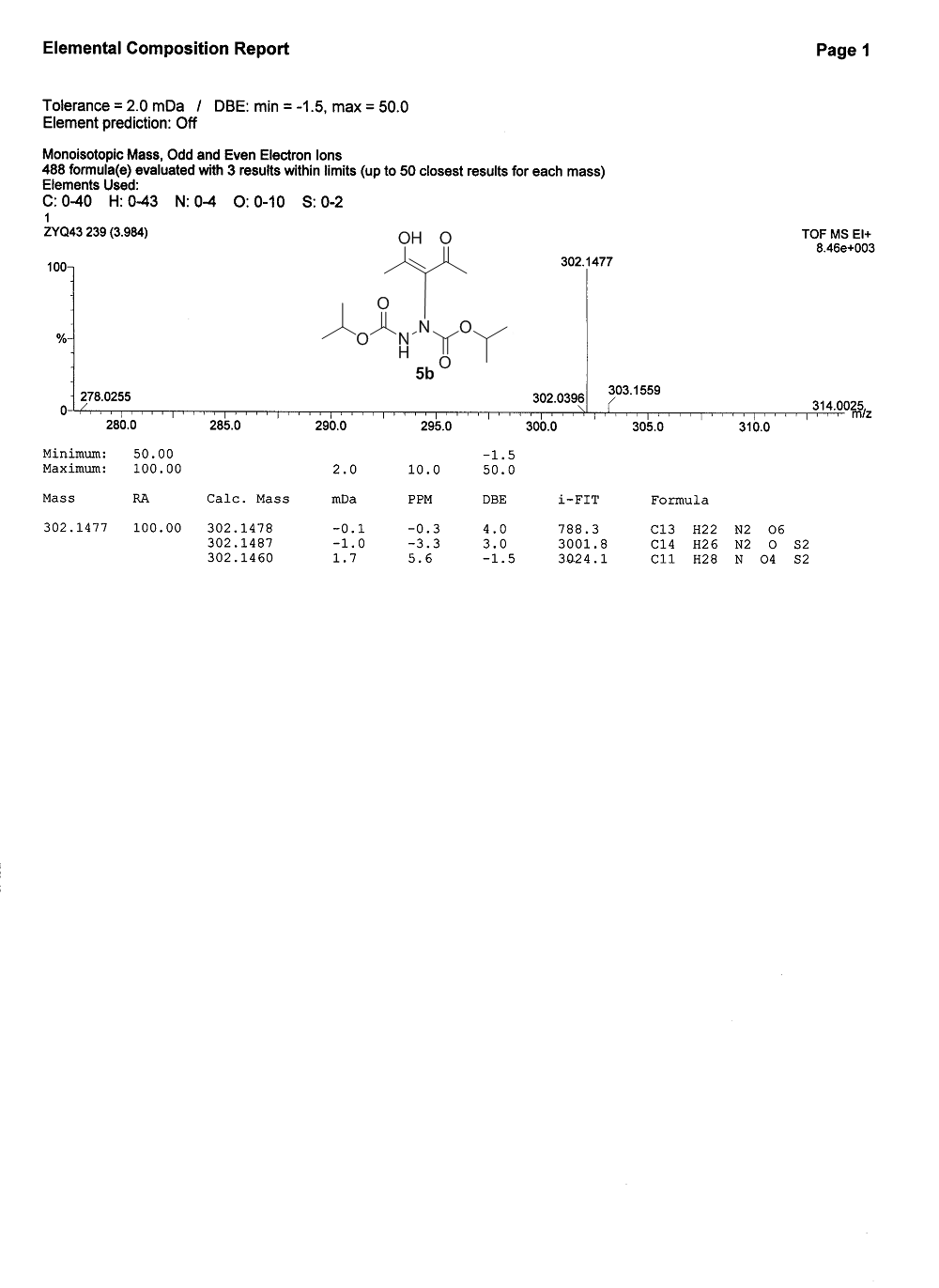


Fig. 50 MS spectrum of product **5b**


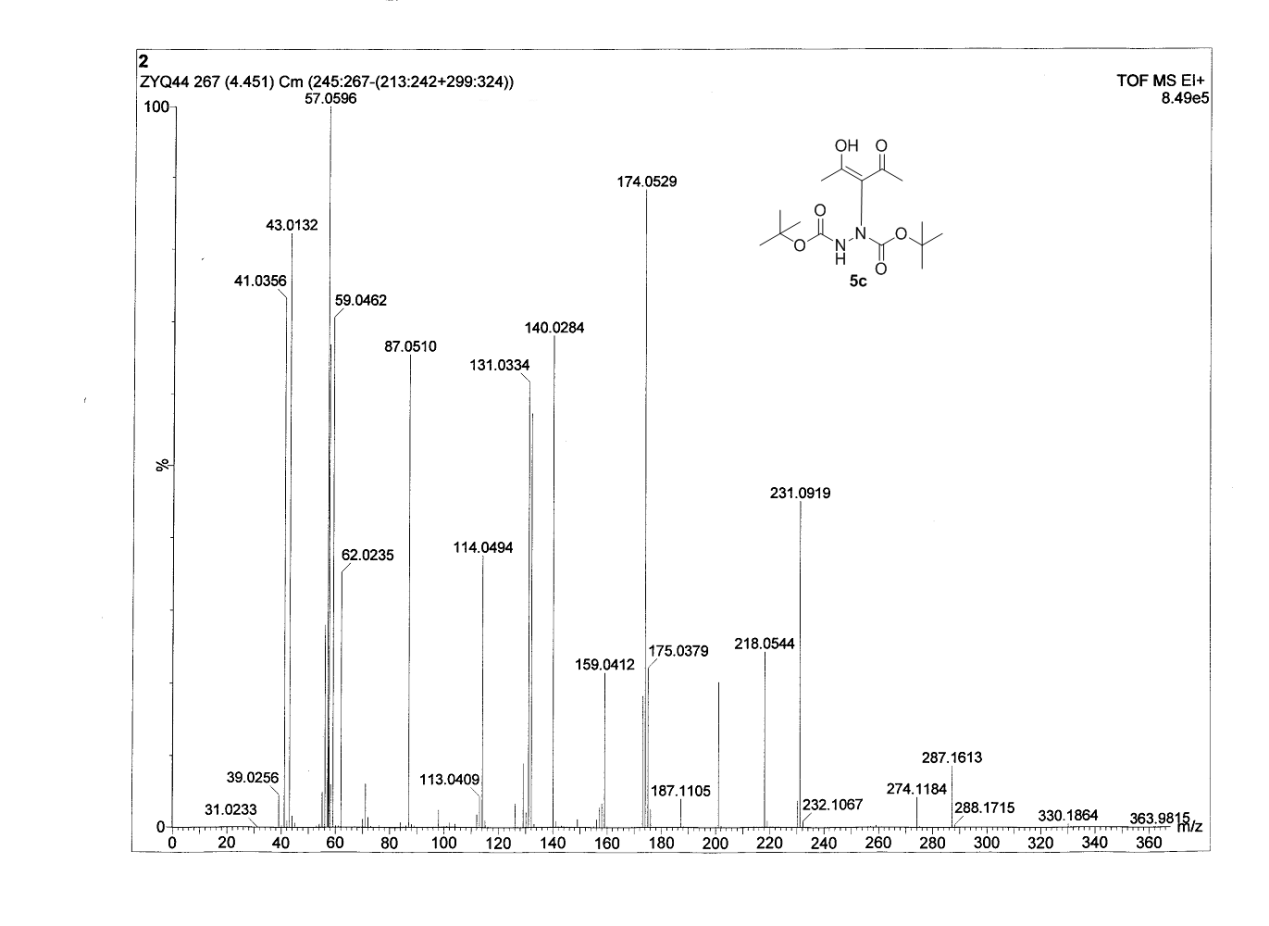


Fig. 51 MS spectrum of product **5c**


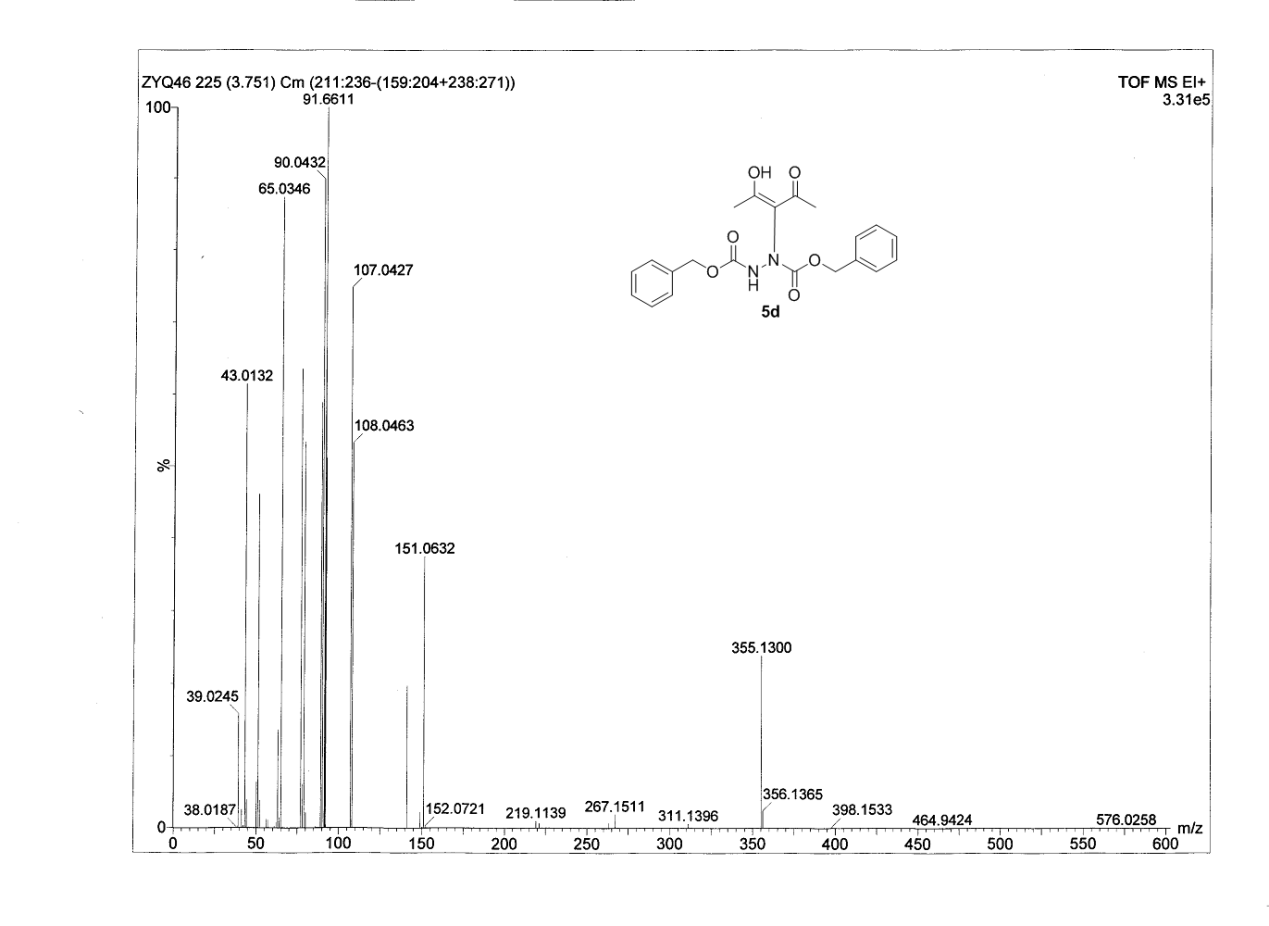


Fig. 52 MS spectrum of product **5d**


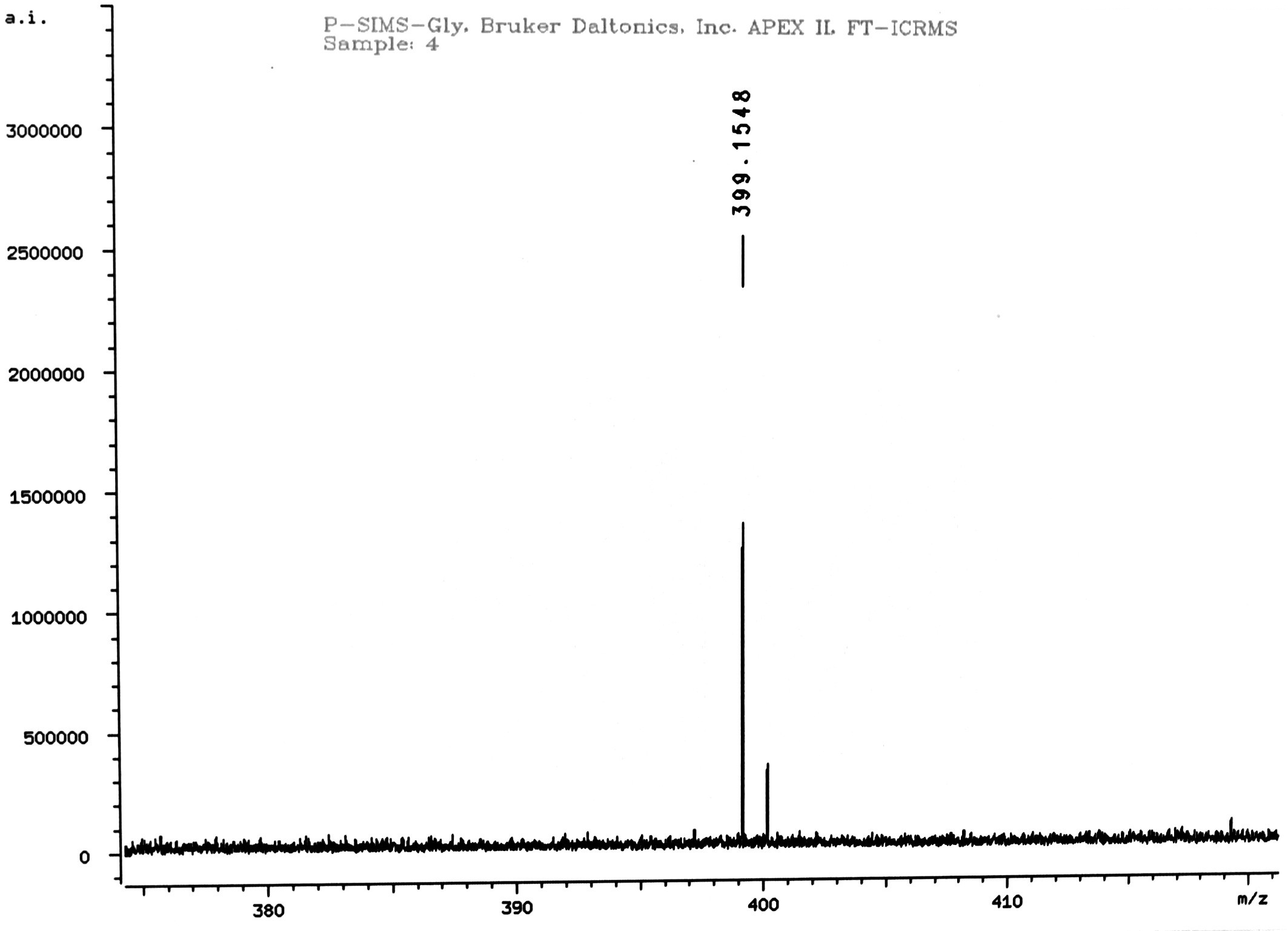


Fig. 53 MS spectrum of product **5e**

**
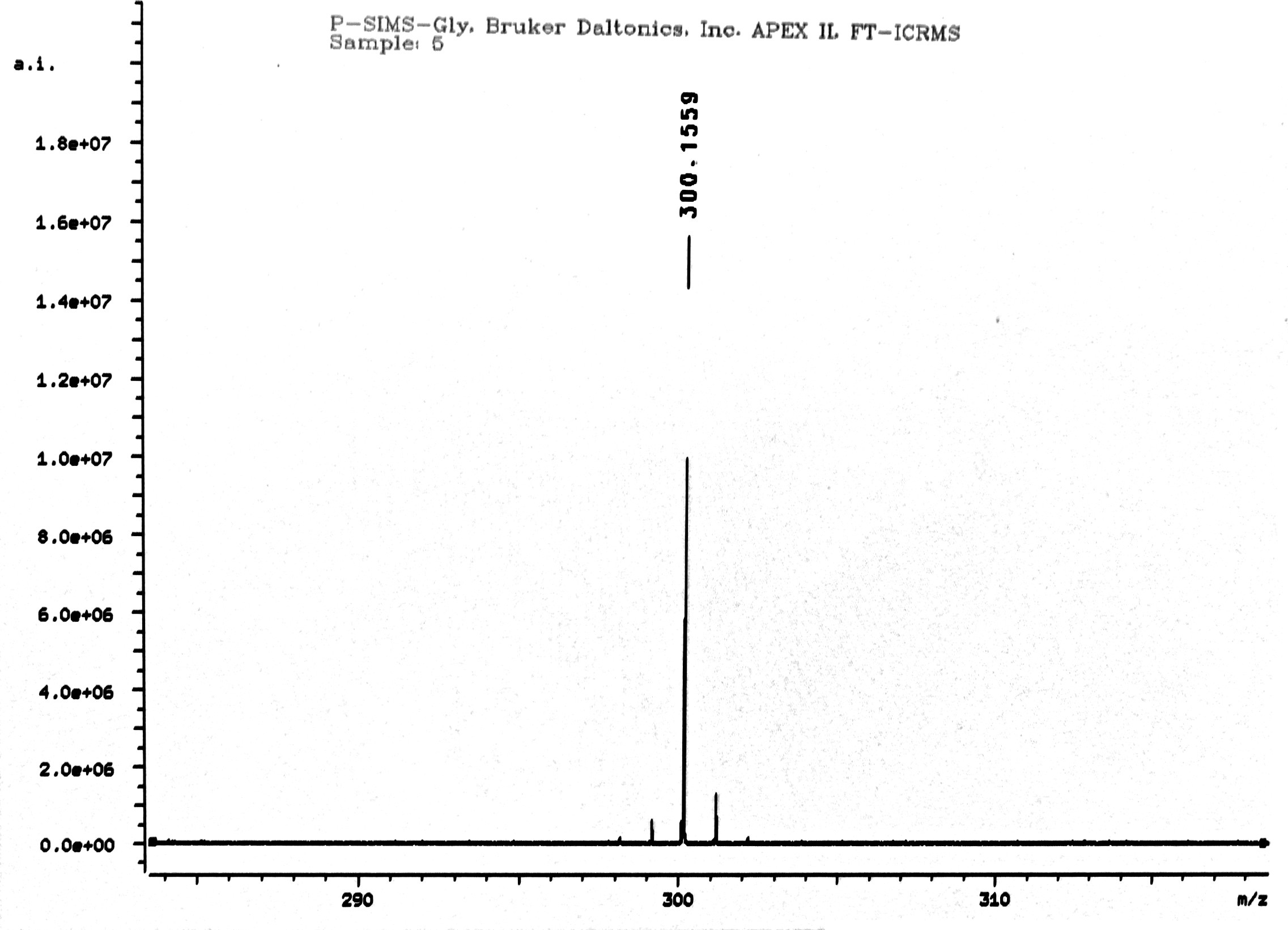
**

Fig. 54 MS spectrum of product **7a**


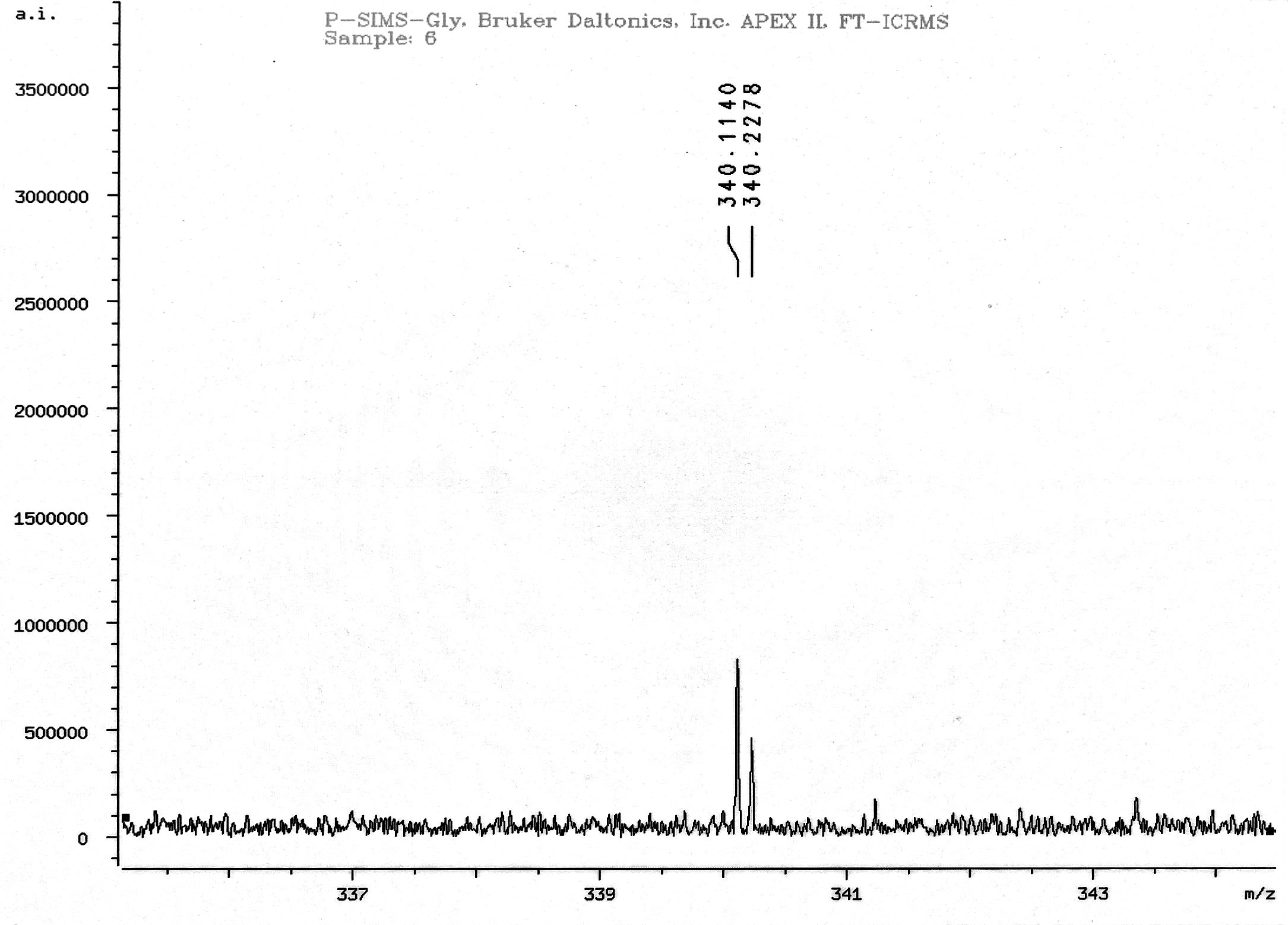


Fig. 55 MS spectrum of product **7b**

**8**. HPLC Spectra for **3a**-**3i**

| Column | CHIRALCEL OD-H(ODH0CE-LA084) |
| --- | --- |
| Column size  Injection  Mobile phase  Flow rate  Wave length  Temperature  Solvents | 0.46cm I.D.*25 cm L  20 µl  *n*-Hexane/2-propanol=95/5 (v/v)  1.0 ml/min  UV 254 nm  25 ^o^C  Hexane, 2-propanol: HPLC grade |

Chromatogram **1:**


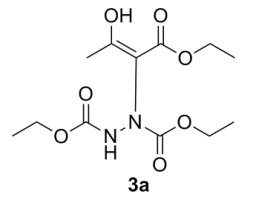

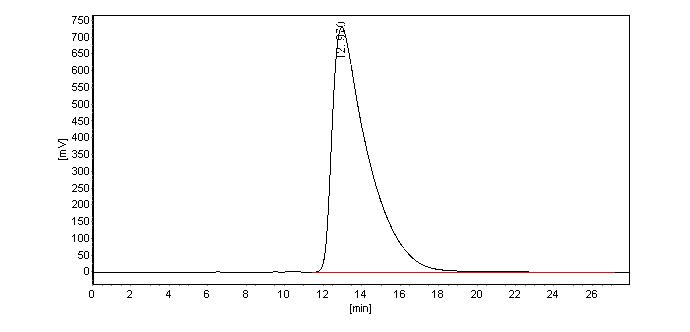


Column performance report for **3a**:

UV 254 nm

| Peak NO. | Time | Height | Area | Area% |
| --- | --- | --- | --- | --- |
| 1 | 12.970 | 730687 | 93742210 | 100 |

| Column | CHIRALCEL OD-H(ODH0CE-LA084) |
| --- | --- |
| Column size  Injection  Mobile phase  Flow rate  Wave length  Temperature  Solvents | 0.46cm I.D.*25 cm L  20 µl  *n*-Hexane/2-propanol=95/5 (v/v)  0.20 ml/min  UV 254 nm  25 ^o^C  Hexane, 2-propanol: HPLC grade |

Chromatogram **2:**


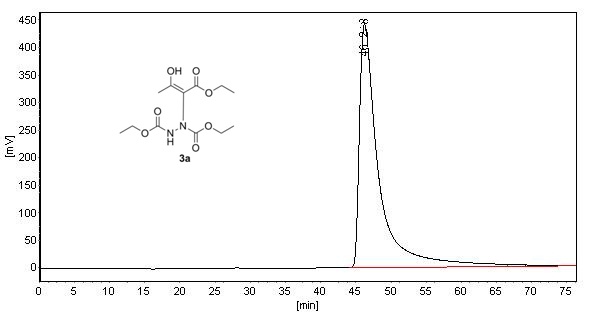


Column performance report for **3a**:

UV 254 nm

| Peak NO. | Time | Height | Area | Area% |
| --- | --- | --- | --- | --- |
| 1 | 46.248 | 441568 | 85069869 | 100 |

| Column | CHIRALCEL OD-H(ODH0CE-LA084) |
| --- | --- |
| Column size  Injection  Mobile phase  Flow rate  Wave length  Temperature  Solvents | 0.46cm I.D.*25 cm L  20 µl  *n*-Hexane/2-propanol=95/5 (v/v)  1.0 ml/min  UV 254 nm  25 ^o^C  Hexane, 2-propanol: HPLC grade |

Chromatogram **3:**


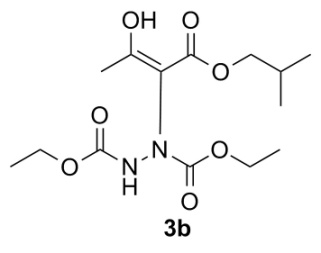

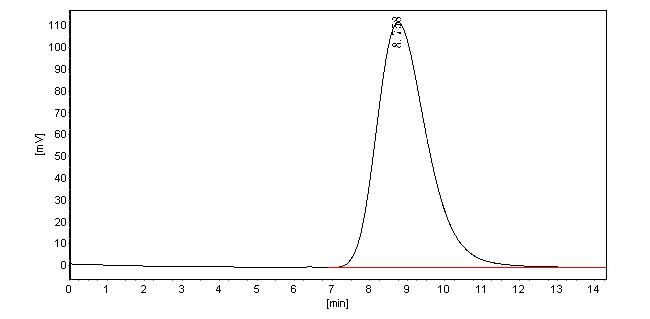


Column performance report for **3b**:

UV 254 nm

| Peak NO. | Time | Height | Area | Area% |
| --- | --- | --- | --- | --- |
| 1 | 8.758 | 112241 | 10557941 | 100 |

| Column | CHIRALCEL OD-H(ODH0CE-LA084) |
| --- | --- |
| Column size  Injection  Mobile phase  Flow rate  Wave length  Temperature  Solvents | 0.46cm I.D.*25 cm L  20 µl  *n*-Hexane/2-propanol=95/5 (v/v)  1.0 ml/min  UV 254 nm  25 ^o^C  Hexane, 2-propanol: HPLC grade |

**C**hromatogram **4:**


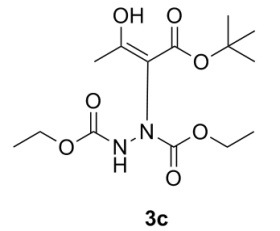

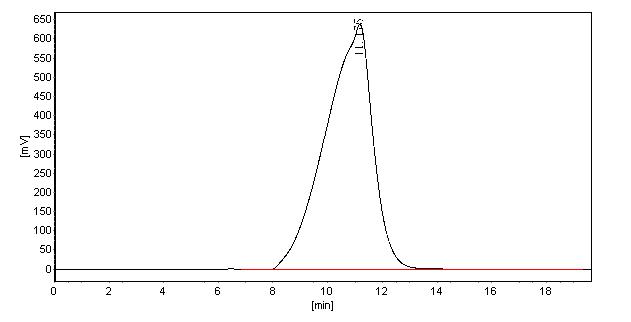


Column performance report for **3c**:

UV 254 nm

| Peak NO. | Time | Height | Area | Area% |
| --- | --- | --- | --- | --- |
| 1 | 11.175 | 639193 | 76986528 | 100 |

| Column | CHIRALCEL OD-H(ODH0CE-LA084) |
| --- | --- |
| Column size  Injection  Mobile phase  Flow rate  Wave length  Temperature  Solvents | 0.46cm I.D.*25 cm L  20 µl  *n*-Hexane/2-propanol=95/5 (v/v)  1.0 ml/min  UV 254 nm  25 ^o^C  Hexane, 2-propanol: HPLC grade |

Chromatogram **5:**


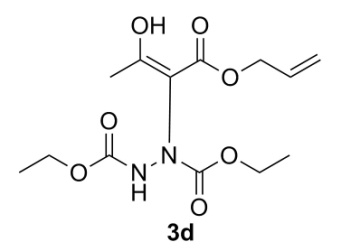

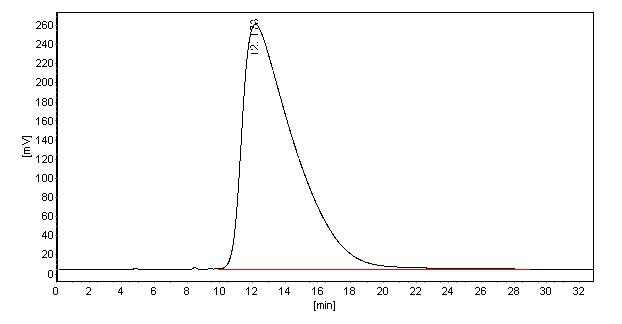


Column performance report for **3d**:

UV 254 nm

| Peak NO. | Time | Height | Area | Area% |
| --- | --- | --- | --- | --- |
| 1 | 12.173 | 256469 | 56538492 | 100 |

| Column | CHIRALCEL OD-H(ODH0CE-LA084) |
| --- | --- |
| Column size  Injection  Mobile phase  Flow rate  Wave length  Temperature  Solvents | 0.46cm I.D.*25 cm L  20 µl  *n*-Hexane/2-propanol=95/5 (v/v)  1.0 ml/min  UV 254 nm  25 ^o^C  Hexane, 2-propanol: HPLC grade |

Chromatogram **6:**


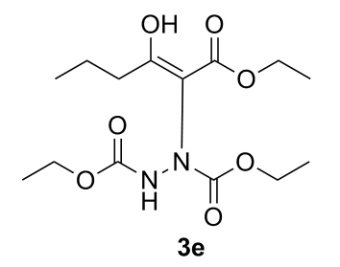

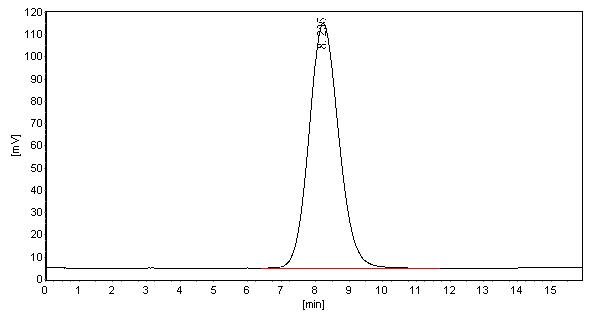


Column performance report for **3e**:

UV 254 nm

| Peak NO. | Time | Height | Area | Area% |
| --- | --- | --- | --- | --- |
| 1 | 8.235 | 110149 | 6749122 | 100 |

| Column | CHIRALCEL OD-H(ODH0CE-LA084) |
| --- | --- |
| Column size  Injection  Mobile phase  Flow rate  Wave length  Temperature  Solvents | 0.46cm I.D.*25 cm L  20 µl  *n*-Hexane/2-propanol=95/5 (v/v)  1.0 ml/min  UV 254 nm  25 ^o^C  Hexane, 2-propanol: HPLC grade |

Chromatogram **7:**


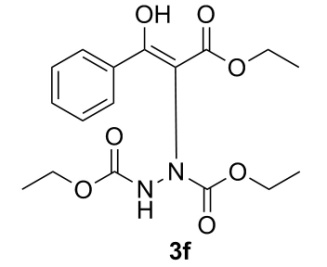

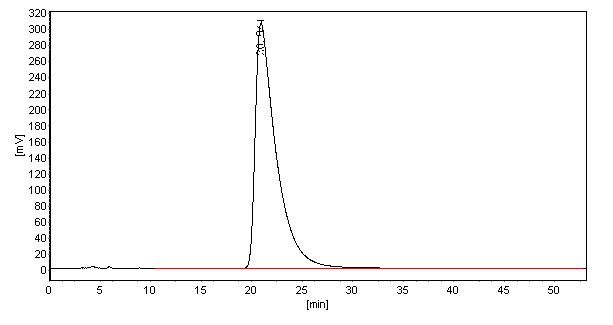


Column performance report for **3f**:

UV 254 nm

| Peak NO. | Time | Height | Area | Area% |
| --- | --- | --- | --- | --- |
| 1 | 20.941 | 304930 | 43950318 | 100 |

| Column | CHIRALCEL OD-H(ODH0CE-LA084) |
| --- | --- |
| Column size  Injection  Mobile phase  Flow rate  Wave length  Temperature  Solvents | 0.46cm I.D.*25 cm L  20 µl  *n*-Hexane/2-propanol=95/5 (v/v)  1.0 ml/min  UV 254 nm  25 ^o^C  Hexane, 2-propanol: HPLC grade |

Chromatogram **8:**


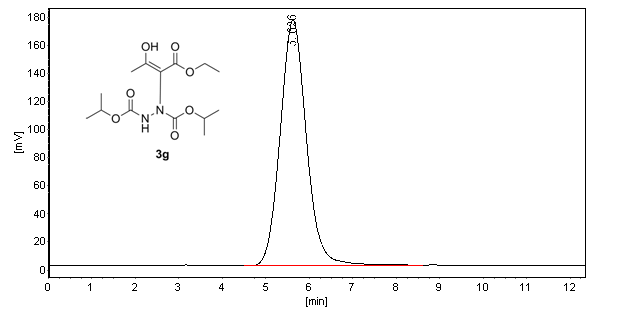


Column performance report for**3g**:

UV 254 nm

| Peak NO. | Time | Height | Area | Area% |
| --- | --- | --- | --- | --- |
| 1 | 5.626 | 174557 | 7302374 | 100 |

| Column | CHIRALCEL OD-H(ODH0CE-LA084) |
| --- | --- |
| Column size  Injection  Mobile phase  Flow rate  Wave length  Temperature  Solvents | 0.46cm I.D.*25 cm L  20 µl  *n*-Hexane/2-propanol=95/5 (v/v)  1.0 ml/min  UV 254 nm  25 ^o^C  Hexane, 2-propanol: HPLC grade |

Chromatogram **9:**


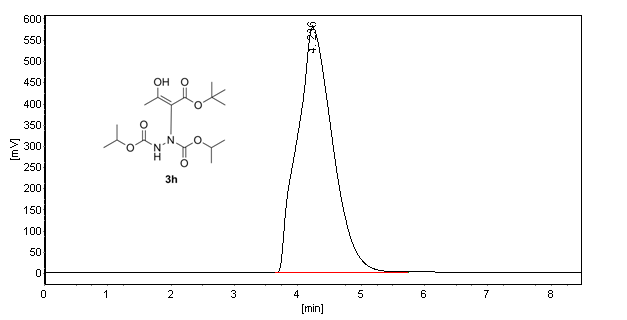


Column performance report for **3h:**

UV 254 nm

| Peak NO. | Time | Height | Area | Area% |
| --- | --- | --- | --- | --- |
| 1 | 4.326 | 577632 | 22180075 | 100 |

| Column | CHIRALCEL OD-H(ODH0CE-LA084) |
| --- | --- |
| Column size  Injection  Mobile phase  Flow rate  Wave length  Temperature  Solvents | 0.46cm I.D.*25 cm L  20 µl  *n*-Hexane/2-propanol=95/5 (v/v)  1.0 ml/min  UV 254 nm  25 ^o^C  Hexane, 2-propanol: HPLC grade |

Chromatogram **10:**


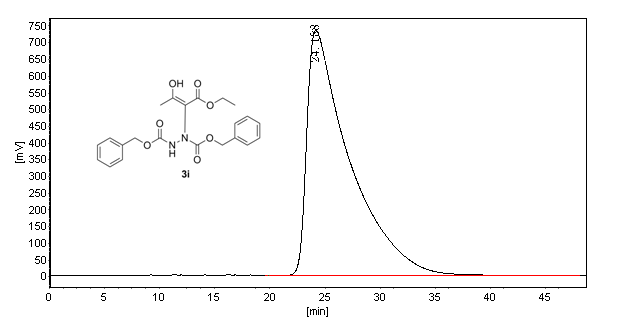


Column performance report for **3i**:

UV 254 nm

| Peak NO. | Time | Height | Area | Area% |
| --- | --- | --- | --- | --- |
| 1 | 24.188 | 733425 | 196620991 | 100 |

| Column | CHIRALCEL OD-H(ODH0CE-LA084) |
| --- | --- |
| Column size  Injection  Mobile phase  Flow rate  Wave length  Temperature  Solvents | 0.46cm I.D.*25 cm L  20 µl  *n*-Hexane/2-propanol=95/5 (v/v)  1.0 ml/min  UV 254 nm  25 ^o^C  Hexane, 2-propanol: HPLC grade |

Chromatogram **11:**


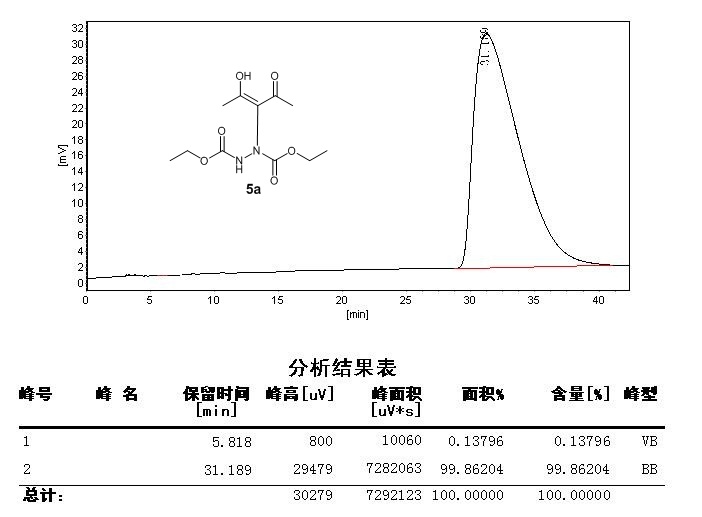


Column performance report for **5a**:

UV 254 nm

| Peak NO. | Time | Height | Area | Area% |
| --- | --- | --- | --- | --- |
| 1 | 31.189 | 29479 | 7282063 | 100 |

| Column | CHIRALCEL OD-H(ODH0CE-LA084) |
| --- | --- |
| Column size  Injection  Mobile phase  Flow rate  Wave length  Temperature  Solvents | 0.46cm I.D.*25 cm L  20 µl  *n*-Hexane/2-propanol=95/5 (v/v)  1.0 ml/min  UV 254 nm  25 ^o^C  Hexane, 2-propanol: HPLC grade |

Chromatogram **12:**


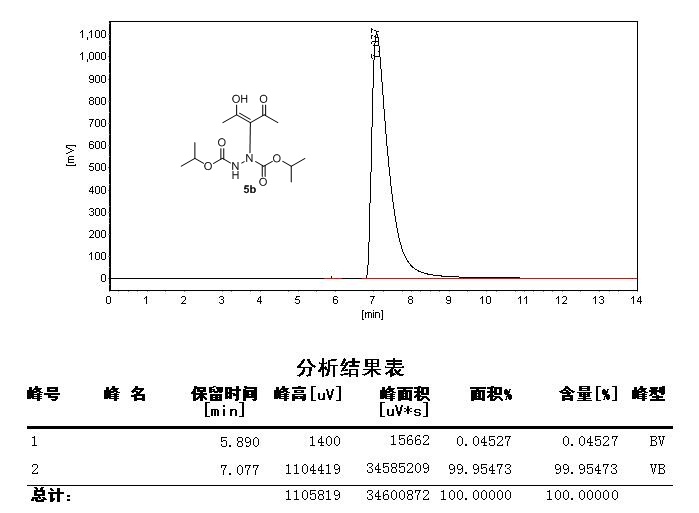


Column performance report for **5b**:

UV 254 nm

| Peak NO. | Time | Height | Area | Area% |
| --- | --- | --- | --- | --- |
| 1 | 7. 077 | 1104419 | 34585209 | 100 |

| Column | CHIRALCEL OD-H(ODH0CE-LA084) |
| --- | --- |
| Column size  Injection  Mobile phase  Flow rate  Wave length  Temperature  Solvents | 0.46cm I.D.*25 cm L  20 µl  *n*-Hexane/2-propanol=95/5 (v/v)  1.0 ml/min  UV 254 nm  25 ^o^C  Hexane, 2-propanol: HPLC grade |

Chromatogram **13:**


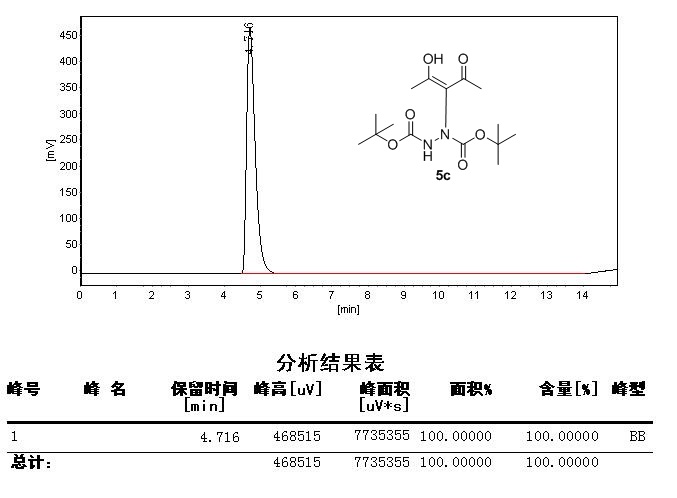


Column performance report for **5c**:

UV 254 nm

| Peak NO. | Time | Height | Area | Area% |
| --- | --- | --- | --- | --- |
| 1 | 4.716 | 468515 | 7735355 | 100 |

| Column | CHIRALCEL OD-H(ODH0CE-LA084) |
| --- | --- |
| Column size  Injection  Mobile phase  Flow rate  Wave length  Temperature  Solvents | 0.46cm I.D.*25 cm L  20 µl  *n*-Hexane/2-propanol=95/5 (v/v)  1.0 ml/min  UV 254 nm  25 ^o^C  Hexane, 2-propanol: HPLC grade |

Chromatogram **14:**


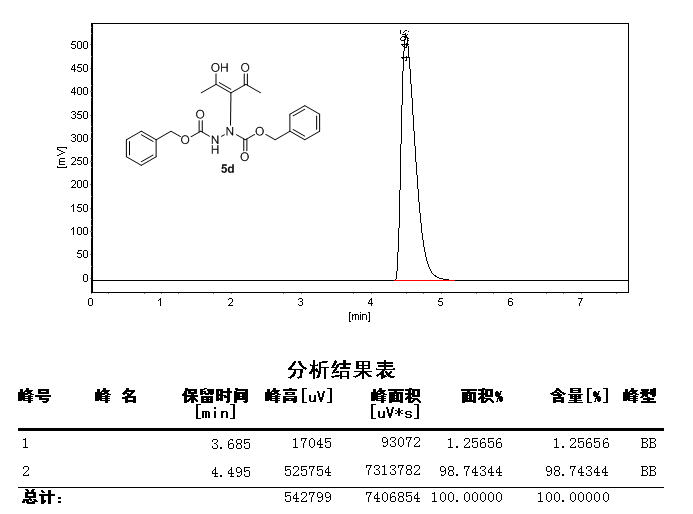


Column performance report for **5d**:

UV 254 nm

| Peak NO. | Time | Height | Area | Area% |
| --- | --- | --- | --- | --- |
| 1 | 4.495 | 525754 | 7313782 | 100 |

| Column | CHIRALCEL OD-H(ODH0CE-LA084) |
| --- | --- |
| Column size  Injection  Mobile phase  Flow rate  Wave length  Temperature  Solvents | 0.46cm I.D.*25 cm L  20 µl  *n*-Hexane/2-propanol=95/5 (v/v)  1.0 ml/min  UV 254 nm  25 ^o^C  Hexane, 2-propanol: HPLC grade |

Chromatogram **15:**


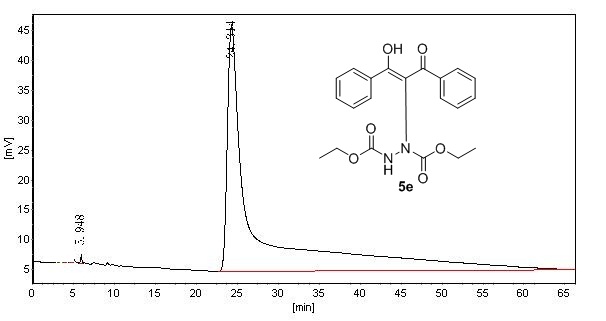


Column performance report for **5e**:

UV 254 nm

| Peak NO. | Time | Height | Area | Area% |
| --- | --- | --- | --- | --- |
| 1 | 5.948 | 1176 | 13992 | 0.15413 |
| 2 | 24.314 | 41060 | 9063983 | 99.84586 |

| Column | CHIRALCEL OD-H(ODH0CE-LA084) |
| --- | --- |
| Column size  Injection  Mobile phase  Flow rate  Wave length  Temperature  Solvents | 0.46cm I.D.*25 cm L  20 µl  *n*-Hexane/2-propanol=95/5 (v/v)  1.0 ml/min  UV 254 nm  25 ^o^C  Hexane, 2-propanol: HPLC grade |

Chromatogram **16:**


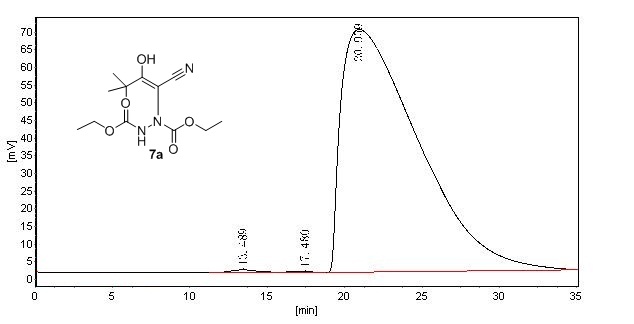


Column performance report for **7a**:

UV 254 nm

| Peak NO. | Time | Height | Area | Area% |
| --- | --- | --- | --- | --- |
| 1 | 13.489 | 836 | 101565 | 0.42242 |
| 2 | 17.480 | 264 | 32439 | 0.13492 |
| 3 | 20.959 | 68755 | 23909363 | 99.44266 |

| Column | CHIRALCEL OD-H(ODH0CE-LA084) |
| --- | --- |
| Column size  Injection  Mobile phase  Flow rate  Wave length  Temperature  Solvents | 0.46cm I.D.*25 cm L  20 µl  *n*-Hexane/2-propanol=95/5 (v/v)  1.0 ml/min  UV 254 nm  25 ^o^C  Hexane, 2-propanol: HPLC grade |

Chromatogram **17:**


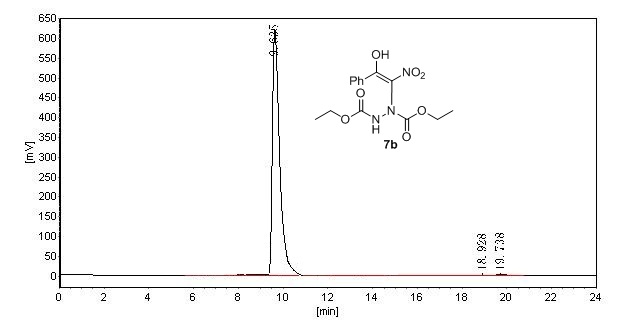


Column performance report for **7b**:

UV 254 nm

| Peak NO. | Time | Height | Area | Area% |
| --- | --- | --- | --- | --- |
| 1 | 9.635 | 618993 | 14776109 | 99.51565 |
| 2 | 18.928 | 384 | 12705 | 0.08557 |
| 3 | 19.738 | 2330 | 59212 | 0.03988 |

| Column | CHIRALCEL AD-H |
| --- | --- |
| Injection  Mobile phase  Flow rate  Wave length  Temperature  Solvents | 20 µl  *n*-Hexane/2-propanol=90/10 (v/v)  2.0 ml/min  UV 254 nm  25 ^o^C  Hexane, 2-propanol: HPLC grade |

Chromatogram **18:**


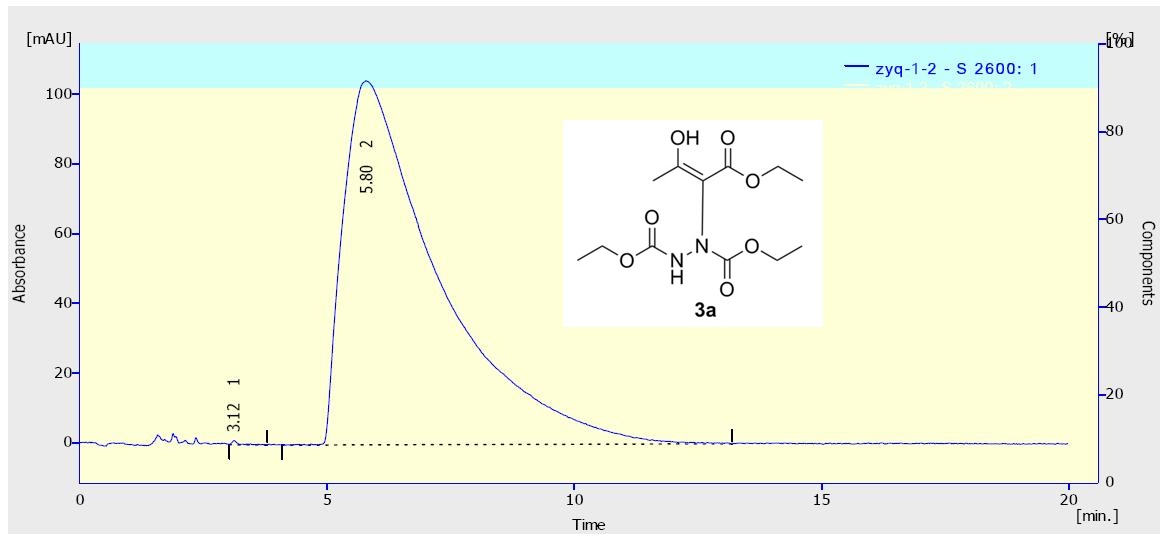


Column performance report for **3a**:

UV 254 nm

| Peak NO. | Time | Height | Area | Area% |
| --- | --- | --- | --- | --- |
| 1 | 3.117 | 1.217 | 13.261 | 0.1 |
| 2 | 5.800 | 104.312 | 14044.808 | 99.9 |

| Column | CHIRALCEL AD-H |
| --- | --- |
| Injection  Mobile phase  Flow rate  Wave length  Temperature  Solvents | 20 µl  *n*-Hexane/2-propanol=90/10 (v/v)  2.0 ml/min  UV 254 nm  25 ^o^C  Hexane, 2-propanol: HPLC grade |

Chromatogram **19:**


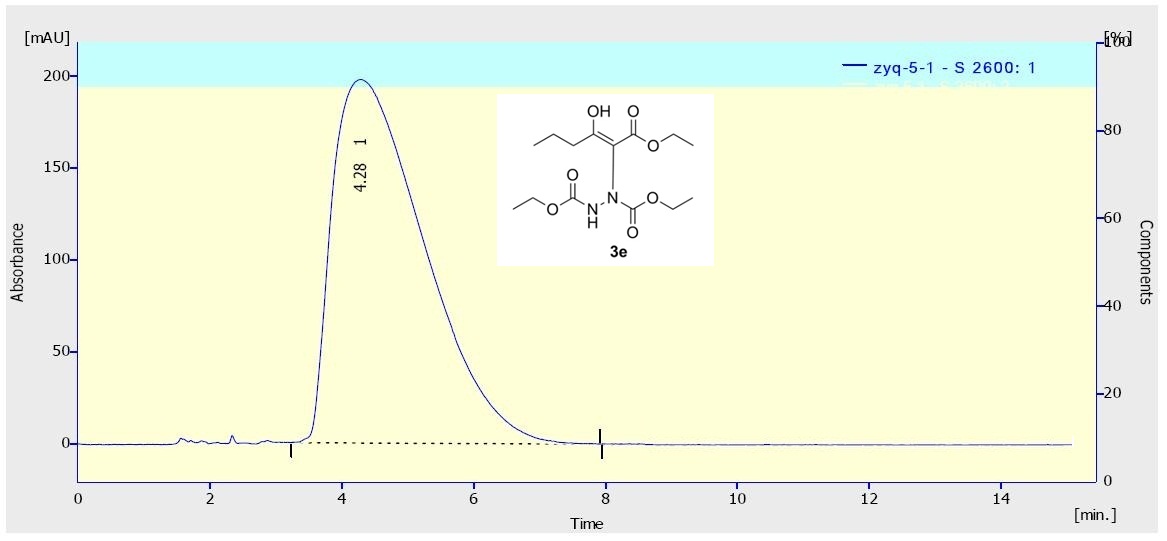


Column performance report for **3e**:

UV 254 nm

| Peak NO. | Time | Height | Area | Area% |
| --- | --- | --- | --- | --- |
| 1 | 4.283 | 197.668 | 19212.705 | 100 |

| Column | CHIRALCEL AD-H |
| --- | --- |
| Injection  Mobile phase  Flow rate  Wave length  Temperature  Solvents | 20 µl  *n*-Hexane/2-propanol=90/10 (v/v)  2.0 ml/min  UV 254 nm  25 ^o^C  Hexane, 2-propanol: HPLC grade |

Chromatogram **20:**


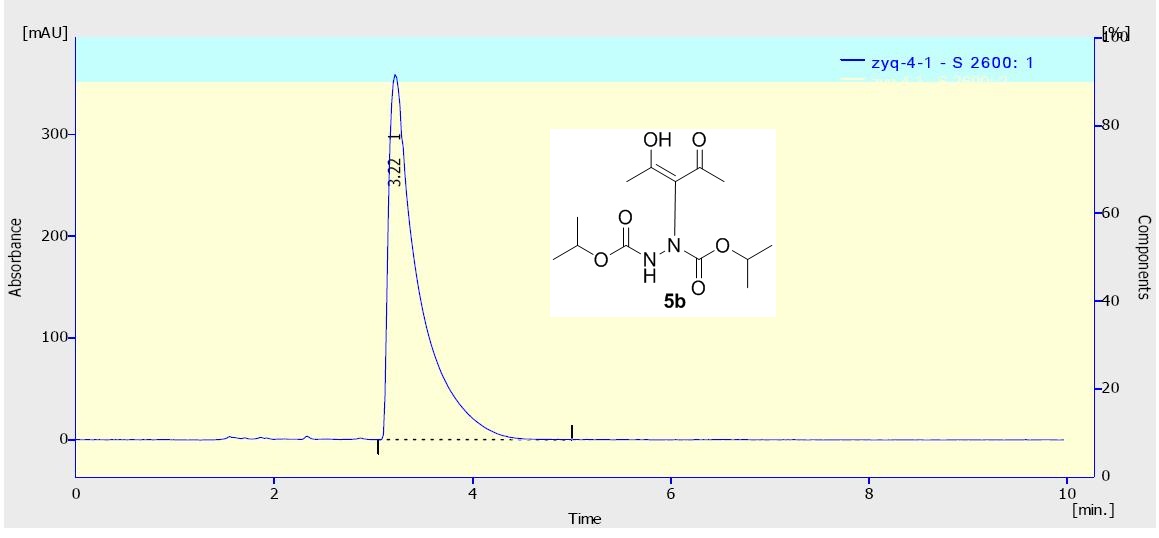


Column performance report for **5b**:

UV 254 nm

| Peak NO. | Time | Height | Area | Area% |
| --- | --- | --- | --- | --- |
| 1 | 3.217 | 359.008 | 7520.690 | 100 |

| Column | CHIRALCEL AD-H |
| --- | --- |
| Injection  Mobile phase  Flow rate  Wave length  Temperature  Solvents | 20 µl  *n*-Hexane/2-propanol=90/10 (v/v)  2.0 ml/min  UV 254 nm  25 ^o^C  Hexane, 2-propanol: HPLC grade |

Chromatogram **21:**


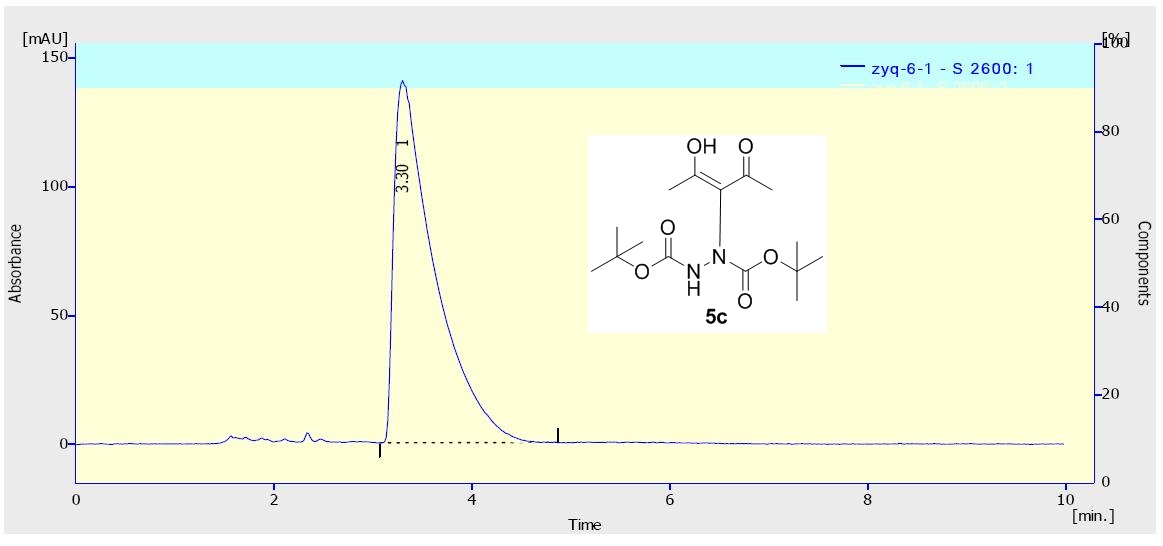


Column performance report for **5c**:

UV 254 nm

| Peak NO. | Time | Height | Area | Area% |
| --- | --- | --- | --- | --- |
| 1 | 3.300 | 140.663 | 4029.974 | 100 |

**9**. Singal crystal X-ray structure of **3a**:

Table 1. Crystal data and structure refinement for **3a**.

Empirical formula C_12_H_20_N_2_O_7_

Formula weight 304.30

Temperature 173(2) K

Wavelength 0.71073 Ǻ

Crystal system, space group Monoclinic, C2/c

Unit cell dimensions a = 19.957(4) Ǻ alpha = 90 deg.

b = 9.858(2) Ǻ beta = 99.02(3) deg.

c = 15.780(3) Ǻ gamma = 90 deg.

Volume 3066.0(11) Ǻ^3^

Z, Calculated density 8, 1.318 Mg/m^3^

Absorption coefficient 0.109 mm^-1^

F(000) 1296

Crystal size 0.57 x 0.52 x 0.50 mm

Theta range for data collection 2.31 to 27.48 deg.

Limiting indices -19<=h<=25, -12<=k<=12, -20<=l<=20

Reflections collected / unique 13194 / 3509 [R(int) = 0.0428]

Completeness to theta = 27.48 99.7 %

Absorption correction Semi-empirical from equivalents

Max. and min. transmission 1.0000 and 0.7302

Refinement method Full-matrix least-squares on F^2

Data / restraints / parameters 3509 / 147 / 202

Goodness-of-fit on F^2 1.215

Final R indices [I>2sigma(I)] R1 = 0.0862, wR2 = 0.2161

R indices (all data) R1 = 0.0941, wR2 = 0.2220

Largest diff. peak and hole 0.513 and -0.315 e. Ǻ^-3^

Table 2. Atomic coordinates ( x 10^4^) and equivalent isotropic

displacement parameters (Ǻ^2^ x 10^3^) for **3a**.

U(eq) is defined as one third of the trace of the orthogonalized

Uij tensor.

________________________________________________________________

x y z U(eq)

________________________________________________________________

O(1) 926(1) 5548(2) -2171(1) 47(1)

O(2) 1313(1) 8004(2) -2354(1) 50(1)

O(3) 929(1) 9595(2) -1531(1) 49(1)

O(4) 290(1) 8336(2) 750(1) 43(1)

O(5) 1110(1) 7038(2) 322(1) 41(1)

O(6) -1026(1) 6962(2) -689(1) 49(1)

O(7) -1359(1) 9164(2) -858(1) 43(1)

N(1) 282(1) 7846(2) -663(1) 36(1)

N(2) -283(1) 8673(2) -878(2) 40(1)

C(1) 272(2) 4948(3) -1105(2) 46(1)

C(2) 608(1) 6022(3) -1543(2) 38(1)

C(3) 619(1) 7354(3) -1334(2) 37(1)

C(4) 982(2) 8326(3) -1789(2) 40(1)

C(5) 1216(2) 10639(3) -2021(2) 66(1)

C(6') 1075(9) 11956(7) -1684(9) 54(3)

C(6) 1369(11) 11827(11) -1496(8) 61(3)

C(7) 549(1) 7795(3) 195(2) 35(1)

C(8) 1412(2) 6889(4) 1222(2) 49(1)

C(9) 2022(2) 6015(4) 1252(2) 67(1)

C(10) -903(1) 8146(3) -798(2) 39(1)

C(11) -2057(2) 8738(4) -904(2) 57(1)

C(12) -2477(2) 9972(4) -940(3) 80(1)

________________________________________________________________

Table 3. Bond lengths [Å] and angles [°] for **3a**.

_____________________________________________________________

O(1)-C(2) 1.342(3)

O(1)-H(1) 0.8400

O(2)-C(4) 1.233(3)

O(3)-C(4) 1.325(3)

O(3)-C(5) 1.456(4)

O(4)-C(7) 1.209(3)

O(5)-C(7) 1.334(3)

O(5)-C(8) 1.460(3)

O(6)-C(10) 1.210(3)

O(7)-C(10) 1.347(3)

O(7)-C(11) 1.447(4)

N(1)-C(7) 1.375(3)

N(1)-N(2) 1.389(3)

N(1)-C(3) 1.426(3)

N(2)-C(10) 1.367(4)

N(2)-H(2) 0.8900

C(1)-C(2) 1.482(4)

C(1)-H(1A) 0.9800

C(1)-H(1B) 0.9800

C(1)-H(1C) 0.9800

C(2)-C(3) 1.354(4)

C(3)-C(4) 1.457(4)

C(5)-C(6) 1.440(7)

C(5)-C(6') 1.448(6)

C(5)-H(5A) 0.9900

C(5)-H(5B) 0.9900

C(6')-H(6'1) 0.9800

C(6')-H(6'2) 0.9800

C(6')-H(6'3) 0.9800

C(6)-H(6A) 0.9800

C(6)-H(6B) 0.9800

C(6)-H(6C) 0.9800

C(8)-C(9) 1.486(5)

C(8)-H(8A) 0.9900

C(8)-H(8B) 0.9900

C(9)-H(9A) 0.9800

C(9)-H(9B) 0.9800

C(9)-H(9C) 0.9800

C(11)-C(12) 1.473(5)

C(11)-H(11A) 0.9900

C(11)-H(11B) 0.9900

C(12)-H(12A) 0.9800

C(12)-H(12B) 0.9800

C(12)-H(12C) 0.9800

C(2)-O(1)-H(1) 104.2

C(4)-O(3)-C(5) 116.6(2)

C(7)-O(5)-C(8) 114.3(2)

C(10)-O(7)-C(11) 115.0(2)

C(7)-N(1)-N(2) 115.9(2)

C(7)-N(1)-C(3) 124.3(2)

N(2)-N(1)-C(3) 118.5(2)

C(10)-N(2)-N(1) 117.7(2)

C(10)-N(2)-H(2) 117.8

N(1)-N(2)-H(2) 119.8

C(2)-C(1)-H(1A) 109.5

C(2)-C(1)-H(1B) 109.5

H(1A)-C(1)-H(1B) 109.5

C(2)-C(1)-H(1C) 109.5

H(1A)-C(1)-H(1C) 109.5

H(1B)-C(1)-H(1C) 109.5

O(1)-C(2)-C(3) 121.8(3)

O(1)-C(2)-C(1) 113.4(2)

C(3)-C(2)-C(1) 124.8(3)

C(2)-C(3)-N(1) 121.3(2)

C(2)-C(3)-C(4) 120.5(3)

N(1)-C(3)-C(4) 118.2(2)

O(2)-C(4)-O(3) 122.9(3)

O(2)-C(4)-C(3) 123.5(3)

O(3)-C(4)-C(3) 113.6(2)

C(6)-C(5)-C(6') 25.1(5)

C(6)-C(5)-O(3) 109.5(5)

C(6')-C(5)-O(3) 108.9(4)

C(6)-C(5)-H(5A) 86.9

C(6')-C(5)-H(5A) 109.9

O(3)-C(5)-H(5A) 109.9

C(6)-C(5)-H(5B) 129.0

C(6')-C(5)-H(5B) 109.9

O(3)-C(5)-H(5B) 109.9

H(5A)-C(5)-H(5B) 108.3

C(5)-C(6')-H(6'1) 109.5

C(5)-C(6')-H(6'2) 109.5

C(5)-C(6')-H(6'3) 109.5

C(5)-C(6)-H(6A) 109.5

C(5)-C(6)-H(6B) 109.5

H(6A)-C(6)-H(6B) 109.5

C(5)-C(6)-H(6C) 109.5

H(6A)-C(6)-H(6C) 109.5

H(6B)-C(6)-H(6C) 109.5

O(4)-C(7)-O(5) 125.5(2)

O(4)-C(7)-N(1) 123.6(3)

O(5)-C(7)-N(1) 110.8(2)

O(5)-C(8)-C(9) 107.5(3)

O(5)-C(8)-H(8A) 110.2

C(9)-C(8)-H(8A) 110.2

O(5)-C(8)-H(8B) 110.2

C(9)-C(8)-H(8B) 110.2

H(8A)-C(8)-H(8B) 108.5

C(8)-C(9)-H(9A) 109.5

C(8)-C(9)-H(9B) 109.5

H(9A)-C(9)-H(9B) 109.5

C(8)-C(9)-H(9C) 109.5

H(9A)-C(9)-H(9C) 109.5

H(9B)-C(9)-H(9C) 109.5

O(6)-C(10)-O(7) 125.3(3)

O(6)-C(10)-N(2) 125.9(3)

O(7)-C(10)-N(2) 108.8(2)

O(7)-C(11)-C(12) 107.5(3)

O(7)-C(11)-H(11A) 110.2

C(12)-C(11)-H(11A) 110.2

O(7)-C(11)-H(11B) 110.2

C(12)-C(11)-H(11B) 110.2

H(11A)-C(11)-H(11B) 108.5

C(11)-C(12)-H(12A) 109.5

C(11)-C(12)-H(12B) 109.5

H(12A)-C(12)-H(12B) 109.5

C(11)-C(12)-H(12C) 109.5

H(12A)-C(12)-H(12C) 109.5

H(12B)-C(12)-H(12C) 109.5

_____________________________________________________________

Table 4. Torsion angles [°] for **3a**.

________________________________________________________________

C(7)-N(1)-N(2)-C(10) -83.4(3)

C(3)-N(1)-N(2)-C(10) 109.4(3)

O(1)-C(2)-C(3)-N(1) -179.7(2)

C(1)-C(2)-C(3)-N(1) -1.5(4)

O(1)-C(2)-C(3)-C(4) -0.1(4)

C(1)-C(2)-C(3)-C(4) 178.1(3)

C(7)-N(1)-C(3)-C(2) 81.9(4)

N(2)-N(1)-C(3)-C(2) -112.0(3)

C(7)-N(1)-C(3)-C(4) -97.7(3)

N(2)-N(1)-C(3)-C(4) 68.4(3)

C(5)-O(3)-C(4)-O(2) 7.4(4)

C(5)-O(3)-C(4)-C(3) -173.4(3)

C(2)-C(3)-C(4)-O(2) -2.8(4)

N(1)-C(3)-C(4)-O(2) 176.9(3)

C(2)-C(3)-C(4)-O(3) 178.0(3)

N(1)-C(3)-C(4)-O(3) -2.4(4)

C(4)-O(3)-C(5)-C(6) -157.0(11)

C(4)-O(3)-C(5)-C(6') 176.4(9)

C(8)-O(5)-C(7)-O(4) 0.1(4)

C(8)-O(5)-C(7)-N(1) -177.8(2)

N(2)-N(1)-C(7)-O(4) 5.7(4)

C(3)-N(1)-C(7)-O(4) 172.1(2)

N(2)-N(1)-C(7)-O(5) -176.3(2)

C(3)-N(1)-C(7)-O(5) -9.9(4)

C(7)-O(5)-C(8)-C(9) 179.4(3)

C(11)-O(7)-C(10)-O(6) -8.3(4)

C(11)-O(7)-C(10)-N(2) 171.7(2)

N(1)-N(2)-C(10)-O(6) -12.8(4)

N(1)-N(2)-C(10)-O(7) 167.2(2)

C(10)-O(7)-C(11)-C(12) 178.3(3)

________________________________________________________________

**10.** Single crystal X-ray structure of **5a**


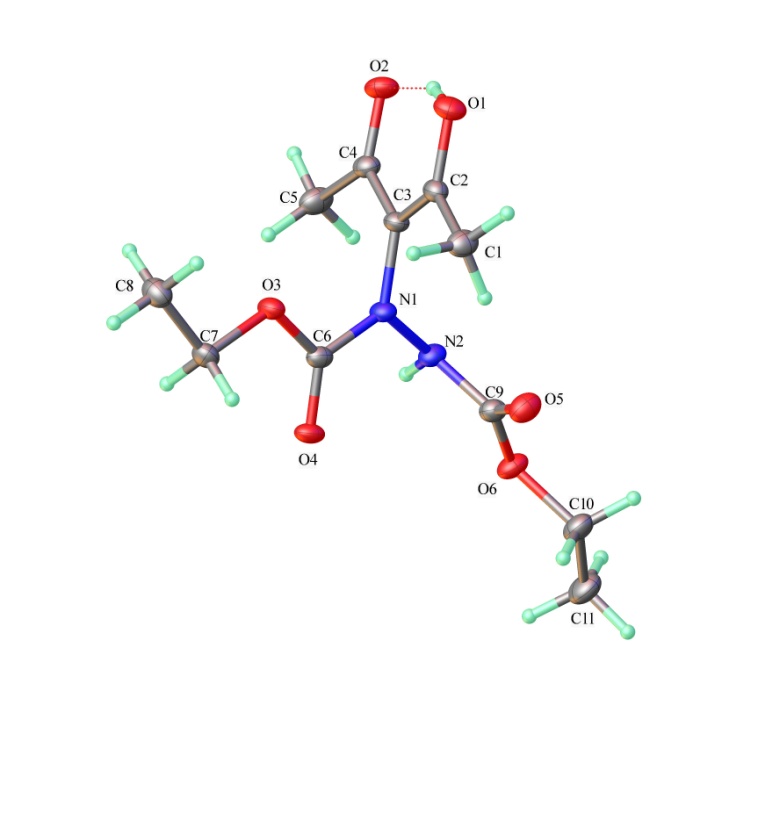


Table 1. Crystal data and structure refinement for **5a**.

Empirical formula C_11_ H_18_ N_2_ O_6_

Formula weight 274.27

Temperature 173(2) K

Wavelength 0.71073 Ǻ

Crystal system, space group Triclinic, P-1

Unit cell dimensions a = 8.6838(17) Ǻ alpha = 80.33(3) deg.

b = 8.8343(18) Ǻ beta = 72.94(3) deg.

c = 10.114(2) Ǻ gamma = 67.07(3) deg.

Volume 681.9(2) Ǻ^3^

Z, Calculated density 2, 1.336 Mg/m^3^

Absorption coefficient 0.109 mm^-1^

F(000) 292

Crystal size 0.17 x 0.12 x 0.06 mm

Theta range for data collection 2.11 to 27.52 deg.

Limiting indices -11<=h<=11, -11<=k<=11, -13<=l<=12

Reflections collected / unique 9173 / 3131 [R(int) = 0.0626]

Completeness to theta = 27.52 99.5 %

Absorption correction Semi-empirical from equivalents

Max. and min. transmission 1.0000 and 0.5418

Refinement method Full-matrix least-squares on F^2^

Data / restraints / parameters 3131 / 0 / 176

Goodness-of-fit on F^2 1.157

Final R indices [I>2sigma(I)] R1 = 0.0653, wR2 = 0.1665

R indices (all data) R1 = 0.0736, wR2 = 0.1736

Largest diff. peak and hole 0.271 and -0.243 e. Ǻ^-3^

Table 2. Atomic coordinates ( x 10^4^) and equivalent isotropic

displacement parameters (Ǻ^2^ x 10^3^) for **5a**.

U(eq) is defined as one third of the trace of the orthogonalized

Uij tensor.

________________________________________________________________

x y z U(eq)

________________________________________________________________

O(1) -225(2) -1849(2) 9697(2) 38(1)

O(2) 2490(2) -2000(2) 10216(1) 40(1)

O(3) 933(2) 1926(2) 6936(1) 32(1)

O(4) 2795(2) 1230(2) 4838(1) 33(1)

O(5) 2324(2) -2399(2) 4697(2) 41(1)

O(6) 5215(2) -3091(2) 3803(1) 37(1)

N(1) 2640(2) -709(2) 6608(2) 28(1)

N(2) 4037(2) -1889(2) 5789(2) 30(1)

C(1) -780(3) -987(3) 7487(2) 39(1)

C(2) 389(3) -1365(2) 8409(2) 30(1)

C(3) 1978(2) -1200(2) 8015(2) 27(1)

C(4) 2976(3) -1472(2) 8991(2) 31(1)

C(5) 4607(3) -1112(3) 8574(2) 43(1)

C(6) 2161(2) 864(2) 6036(2) 28(1)

C(7) 306(3) 3608(2) 6347(2) 35(1)

C(8) -1058(3) 4647(3) 7472(2) 42(1)

C(9) 3707(3) -2436(2) 4741(2) 31(1)

C(10) 5098(3) -3782(3) 2646(2) 42(1)

C(11) 6851(3) -4280(3) 1656(3) 51(1)

________________________________________________________________

Table 3. Bond lengths [Ǻ] and angles [deg] for **5a**.

_____________________________________________________________

O(1)-C(2) 1.322(2)

O(1)-H(1) 0.8400

O(2)-C(4) 1.258(2)

O(3)-C(6) 1.336(2)

O(3)-C(7) 1.457(2)

O(4)-C(6) 1.220(2)

O(5)-C(9) 1.202(2)

O(6)-C(9) 1.347(2)

O(6)-C(10) 1.453(3)

N(1)-C(6) 1.364(2)

N(1)-N(2) 1.400(2)

N(1)-C(3) 1.432(2)

N(2)-C(9) 1.377(3)

N(2)-H(2) 0.8900

C(1)-C(2) 1.483(3)

C(1)-H(1B) 0.9800

C(1)-H(1C) 0.9800

C(1)-H(1A) 0.9800

C(2)-C(3) 1.377(3)

C(3)-C(4) 1.430(3)

C(4)-C(5) 1.497(3)

C(5)-H(5B) 0.9800

C(5)-H(5C) 0.9800

C(5)-H(5A) 0.9800

C(7)-C(8) 1.501(3)

C(7)-H(7A) 0.9900

C(7)-H(7B) 0.9900

C(8)-H(8B) 0.9800

C(8)-H(8C) 0.9800

C(8)-H(8A) 0.9800

C(10)-C(11) 1.499(3)

C(10)-H(10A) 0.9900

C(10)-H(10B) 0.9900

C(11)-H(11A) 0.9800

C(11)-H(11C) 0.9800

C(11)-H(11B) 0.9800

C(2)-O(1)-H(1) 103.0

C(6)-O(3)-C(7) 113.94(15)

C(9)-O(6)-C(10) 115.11(16)

C(6)-N(1)-N(2) 116.28(15)

C(6)-N(1)-C(3) 125.33(15)

N(2)-N(1)-C(3) 117.92(15)

C(9)-N(2)-N(1) 116.75(16)

C(9)-N(2)-H(2) 113.4

N(1)-N(2)-H(2) 117.8

C(2)-C(1)-H(1B) 109.5

C(2)-C(1)-H(1C) 109.5

H(1B)-C(1)-H(1C) 109.5

C(2)-C(1)-H(1A) 109.5

H(1B)-C(1)-H(1A) 109.5

H(1C)-C(1)-H(1A) 109.5

O(1)-C(2)-C(3) 120.58(18)

O(1)-C(2)-C(1) 114.69(17)

C(3)-C(2)-C(1) 124.70(18)

C(2)-C(3)-C(4) 121.42(17)

C(2)-C(3)-N(1) 119.86(17)

C(4)-C(3)-N(1) 118.70(16)

O(2)-C(4)-C(3) 120.57(18)

O(2)-C(4)-C(5) 119.01(19)

C(3)-C(4)-C(5) 120.41(17)

C(4)-C(5)-H(5B) 109.5

C(4)-C(5)-H(5C) 109.5

H(5B)-C(5)-H(5C) 109.5

C(4)-C(5)-H(5A) 109.5

H(5B)-C(5)-H(5A) 109.5

H(5C)-C(5)-H(5A) 109.5

O(4)-C(6)-O(3) 125.04(18)

O(4)-C(6)-N(1) 122.98(18)

O(3)-C(6)-N(1) 111.98(15)

O(3)-C(7)-C(8) 107.80(16)

O(3)-C(7)-H(7A) 110.1

C(8)-C(7)-H(7A) 110.1

O(3)-C(7)-H(7B) 110.1

C(8)-C(7)-H(7B) 110.1

H(7A)-C(7)-H(7B) 108.5

C(7)-C(8)-H(8B) 109.5

C(7)-C(8)-H(8C) 109.5

H(8B)-C(8)-H(8C) 109.5

C(7)-C(8)-H(8A) 109.5

H(8B)-C(8)-H(8A) 109.5

H(8C)-C(8)-H(8A) 109.5

O(5)-C(9)-O(6) 125.78(18)

O(5)-C(9)-N(2) 125.94(18)

O(6)-C(9)-N(2) 108.22(16)

O(6)-C(10)-C(11) 106.97(19)

O(6)-C(10)-H(10A) 110.3

C(11)-C(10)-H(10A) 110.3

O(6)-C(10)-H(10B) 110.3

C(11)-C(10)-H(10B) 110.3

H(10A)-C(10)-H(10B) 108.6

C(10)-C(11)-H(11A) 109.5

C(10)-C(11)-H(11C) 109.5

H(11A)-C(11)-H(11C) 109.5

C(10)-C(11)-H(11B) 109.5

H(11A)-C(11)-H(11B) 109.5

H(11C)-C(11)-H(11B) 109.5

_____________________________________________________________

Symmetry transformations used to generate equivalent atoms:

Table 4. Torsion angles [°] for **5a**.

________________________________________________________________

C(6)-N(1)-N(2)-C(9) -77.1(2)

C(3)-N(1)-N(2)-C(9) 110.34(19)

O(1)-C(2)-C(3)-C(4) -2.8(3)

C(1)-C(2)-C(3)-C(4) 175.29(18)

O(1)-C(2)-C(3)-N(1) 178.63(16)

C(1)-C(2)-C(3)-N(1) -3.2(3)

C(6)-N(1)-C(3)-C(2) 79.1(2)

N(2)-N(1)-C(3)-C(2) -109.1(2)

C(6)-N(1)-C(3)-C(4) -99.4(2)

N(2)-N(1)-C(3)-C(4) 72.4(2)

C(2)-C(3)-C(4)-O(2) 5.8(3)

N(1)-C(3)-C(4)-O(2) -175.64(17)

C(2)-C(3)-C(4)-C(5) -173.44(19)

N(1)-C(3)-C(4)-C(5) 5.1(3)

C(7)-O(3)-C(6)-O(4) 3.2(3)

C(7)-O(3)-C(6)-N(1) -176.52(16)

N(2)-N(1)-C(6)-O(4) 6.2(3)

C(3)-N(1)-C(6)-O(4) 178.09(17)

N(2)-N(1)-C(6)-O(3) -174.06(15)

C(3)-N(1)-C(6)-O(3) -2.2(3)

C(6)-O(3)-C(7)-C(8) 179.94(16)

C(10)-O(6)-C(9)-O(5) 0.7(3)

C(10)-O(6)-C(9)-N(2) 177.94(16)

N(1)-N(2)-C(9)-O(5) -23.4(3)

N(1)-N(2)-C(9)-O(6) 159.42(15)

C(9)-O(6)-C(10)-C(11) 174.20(18)

________________________________________________________________

Symmetry transformations used to generate equivalent atoms:

**11.** Caculation of **3a** and its keto form isomer **3a’**

All calculations were carried out with the Gaussian 09 programs. The geometrical optimizations of all the complexes were performed using M05-2X with the 6-31G** basis set for all atoms. Frequency calculations at the same level were performed to confirm each stationary point to be a minimum. The free energies of solvation in this study were calculated based on the gas phase optimized structures with the polarizable continuum model (PCM) using UA0 radii. The dielectric constant in the PCM calculations was set to 8.93 to simulate dichloromethane (CH_2_Cl_2_), the solvent medium in the experiments. The single point energies were also computed using the M05-2X method with the 6-311++G** basis set for all atoms. The report free energies and enthalpies include zero-point energies and thermal corrections calculated at 298.15K and 1 atm.

Figure 1. The model structure of enol **3a** and its keto form *β*-carbonyl esters **3a'**


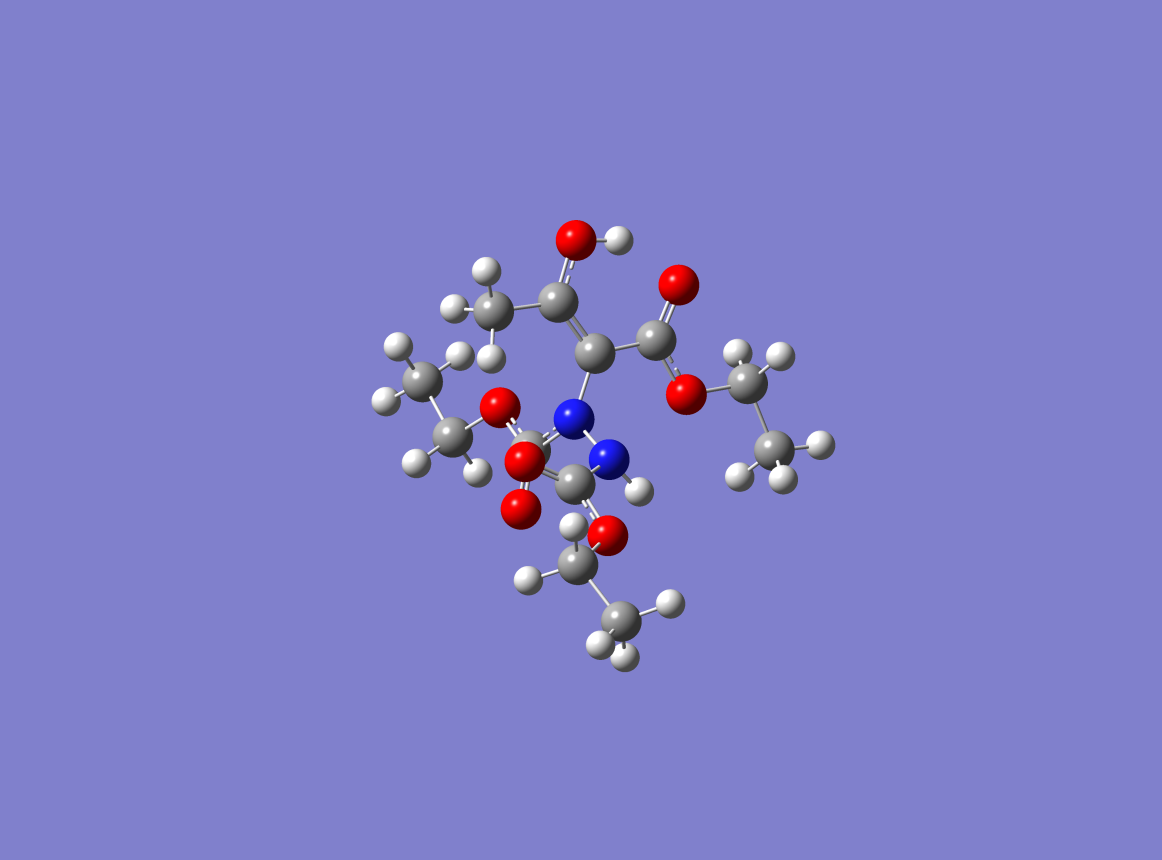


Figure 2. The optimized structure of enol form product **3a**


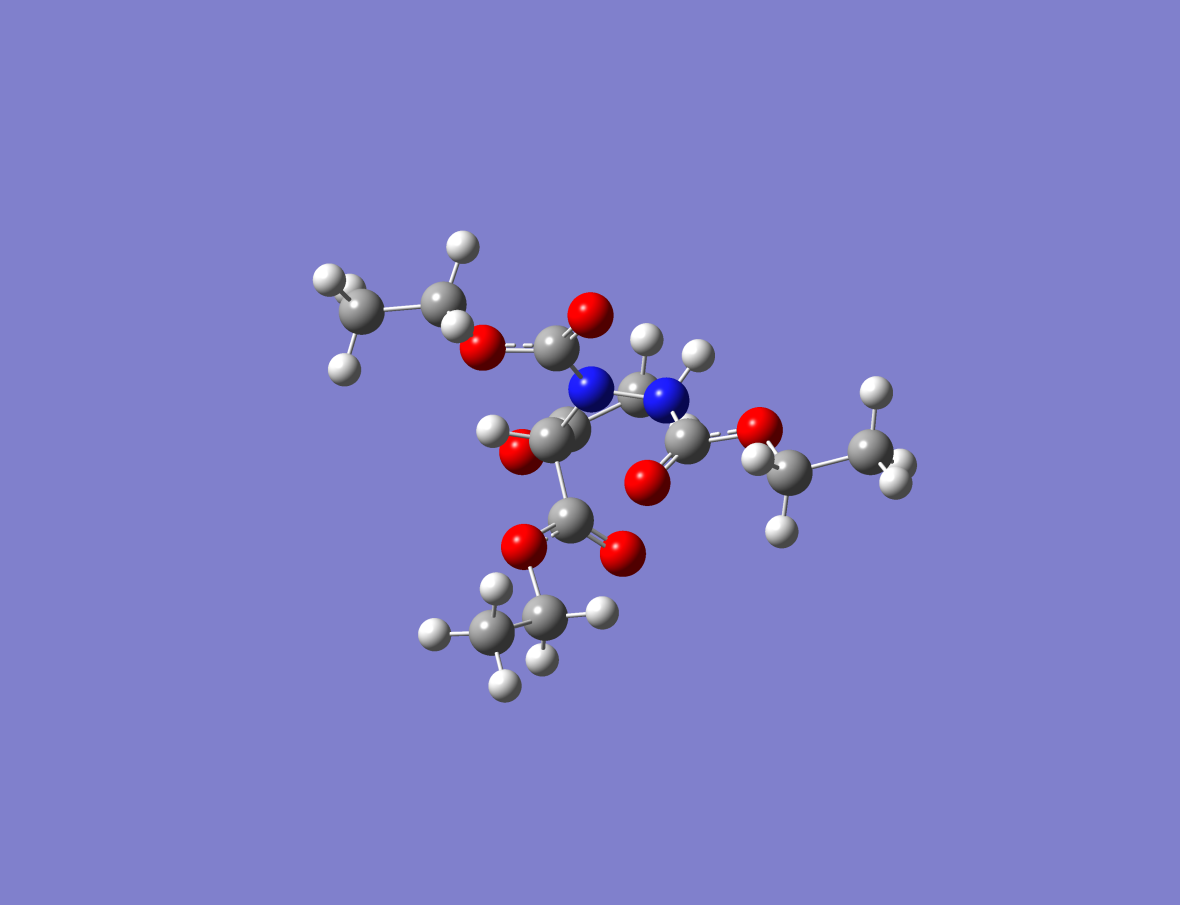


Figure 3. The optimized structure t **3a'**


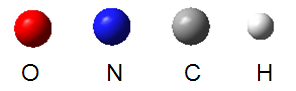


Free energies of gas phase optimized structure:

H_3a_-H_3a_**'** = -9.6Kcal/mol

G_3a_-G_3a_**'** = -8.8Kcal/mol

Free energies of solvational culated based on the gas phase optimized structures with the polarizable continuum model (PCM) using UA0 radii:

H_3a_-H_3a_**'** = -6.9Kcal/mol

G_3a_-G_3a_**'** = -6.2 Kcal/mol

The coordinates (Å), SCF energies, enthalpies at 298K, and Gibbs free energies at 298K for the optimized structures **3a:**

M05-2X SCF energy: -1105.31006693 a.u.

M05-2X enthalpy: -1104.945424 a.u.

M05-2X free energy: -1105.026552 a.u.

M05-2X SCF energy in solution: -1105.62348703 a.u.

M05-2X enthalpy in solution: -1105.258844 a.u.

M05-2X free energy in solution: -1105.339972 a.u.

O -2.34458400 -0.19608800 2.82731400

H -2.68362000 0.68178400 2.51515100

O -2.58479300 1.92164100 1.38610900

O -1.26650900 2.08742700 -0.42842900

O 0.60124300 -1.27751800 -1.89737100

O -1.40533500 -1.69122900 -0.93496900

O 2.32008900 -1.26959200 0.98556400

O 3.39778000 0.42240600 -0.06098000

N -0.00727300 -0.22163400 0.03353700

N 1.17888200 0.47695300 0.00886400

H 1.28831000 1.10314700 -0.77676700

C -0.76798500 -1.88608300 2.30402400

H -0.79331100 -2.04682800 3.38051900

H 0.25118200 -1.93760300 1.92548600

H -1.36691400 -2.66376400 1.82353500

C -1.38035000 -0.56228600 2.00008400

C -1.01686000 0.20265700 0.92689900

C -1.69703200 1.45739500 0.66918900

C -1.91445800 3.34558800 -0.71554100

H -2.97944800 3.16213300 -0.85783000

H -1.79605000 4.00199900 0.14646300

C -1.25644000 3.90268200 -1.95757100

H -0.19296600 4.06804900 -1.78466900

H -1.71653000 4.85449700 -2.22438300

H -1.37135100 3.21311600 -2.79363500

C -0.21345500 -1.08691900 -1.02263400

C -1.66778100 -2.63651600 -1.99298000

H -0.87582400 -3.38538700 -1.99446600

H -1.63770400 -2.10992700 -2.94692500

C -3.02656600 -3.23721200 -1.71263600

H -3.27751600 -3.96183400 -2.48790800

H -3.79174200 -2.46134800 -1.69775000

H -3.02793300 -3.74492200 -0.74802100

C 2.30933500 -0.23750900 0.35722900

C 4.64779900 -0.20933800 0.28196000

H 4.66024800 -1.20924000 -0.15150000

H 4.70265300 -0.30800600 1.36622000

C 5.74846400 0.66835900 -0.26955500

H 6.72017200 0.23005100 -0.03994000

H 5.70119200 1.66353500 0.17221800

H 5.65475300 0.76203300 -1.35132000

The coordinates (Å), SCF energies, enthalpies at 298K, and Gibbs free energies at 298K for the optimized structures **3a':**

M05-2X SCF energy: -1105.29488939 a.u.

M05-2X enthalpy: -1104.930158 a.u.

M05-2X free energy: -1105.012477 a.u.

M05-2X SCF energy in solution: -1105.6125602100 a.u.

M05-2X enthalpy in solution: -1105.247829 a.u.

M05-2X free energy in solution: -1105.330148 a.u.

O -2.30362500 -2.50300800 -1.97723400

O 0.46708300 -2.52120100 -0.05480900

O -0.78120500 -1.49697700 1.51715000

O -0.20945100 2.59681300 -0.44149200

O -2.16899800 1.54297900 -0.04235700

O 1.45953400 0.31659100 1.02164600

O 2.98708500 0.75323000 -0.59236700

N -0.45017700 0.38142000 -0.93235500

N 0.89661500 0.31692700 -1.20797100

H 1.17956300 0.79337000 -2.05130800

C -0.47641900 -1.53455700 -3.17232000

H -0.81368700 -2.18468700 -3.97541800

H 0.53314400 -1.80344800 -2.85641300

H -0.46429900 -0.49482300 -3.50474900

C -1.39590800 -1.70511700 -1.99684400

C -1.16229400 -0.87597000 -0.72736100

C -0.39578400 -1.74233800 0.27122000

C 0.04336300 -2.10272200 2.53900000

H -0.06465500 -3.18558500 2.47062800

H 1.07717000 -1.83415200 2.32897600

C -0.43143600 -1.55237300 3.86385400

H 0.15617000 -1.98316900 4.67534500

H -1.48174600 -1.79326700 4.02792300

H -0.31022400 -0.46960700 3.87957400

C -0.89618800 1.60151400 -0.44417600

C -2.69836500 2.79307900 0.45443600

H -2.63645800 3.53470800 -0.34167700

H -2.07247400 3.12573000 1.28165700

C -4.12288100 2.52293200 0.88122500

H -4.57083400 3.43889300 1.26750000

H -4.14895600 1.76466800 1.66343500

H -4.71632700 2.17402600 0.03636600

C 1.76500000 0.47015700 -0.13676700

C 3.99864000 0.87151600 0.43202700

H 4.05261600 -0.07344800 0.97254700

H 3.69199800 1.64992900 1.13016700

C 5.29506300 1.20623700 -0.26934100

H 6.09565600 1.30503200 0.46427500

H 5.20268600 2.14604000 -0.81309000

H 5.56249200 0.41899100 -0.97377200

H -2.13703300 -0.63917900 -0.31370200

**12.** Caculation of **5a** and its keto form isomer **5a’**

Figure 4. The model structure of enol **5a** and its keto form *β*-carbonyl esters **5a'**

Figure 5. The optimized structure of enol form product **5a**

Figure 6. The optimized structure of **5a'**

Free energies of gas phase optimized structure:

H_5a_ - H_5a_**'** = -9.4 Kcal/mol

G_5a_ - G_5a_**'** = -9.5 Kcal/mol

Free energies of solvational culated based on the gas phase optimized structures with the polarizable continuum model (PCM) using UA0 radii:

H_5a_- H_5a_**'** = -6.5 Kcal/mol

G_5a_- G_5a_**'** = -6.7 Kcal/mol

The coordinates (Å), SCF energies, enthalpies at 298K, and Gibbs free energies at 298K for the optimized structures **5a:**

M05-2X SCF energy: -990.77392552 a.u.

M05-2X enthalpy: -990.4468925 a.u.

M05-2X free energy: -990.5221895 a.u.

M05-2X SCF energy in solution: -991.05494894 a.u.

M05-2X enthalpy in solution: -990.7279159 a.u.

M05-2X free energy in solution: -990.8032129 a.u.

O 2.49564000 -2.01972800 1.66488200

H 2.65875600 -2.57458500 0.81055100

O 2.44142100 -2.89426800 -0.61447900

O -0.39363800 2.02282300 -0.95943600

O 1.70431900 1.52652100 -0.26877000

O -1.89501200 0.25212700 1.41882300

O -3.30969500 -0.17413800 -0.29272500

N 0.15944200 -0.10655600 -0.35641900

N -1.13774400 -0.49535200 -0.63115400

H -1.41705100 -0.34660900 -1.59191500

C 1.22021700 -0.16449800 2.40320600

H 1.33661100 -0.65312000 3.36878800

H 0.20106200 0.18940100 2.26473300

H 1.90412100 0.68674600 2.35850500

C 1.60059600 -1.12688300 1.33153200

C 1.10822900 -1.08405700 0.03982200

C 1.61297800 -2.01531900 -0.93214400

C 0.43186800 1.23091700 -0.55952400

C 2.04450300 2.91924800 -0.44376400

H 1.37400700 3.51940900 0.17080300

H 1.88070900 3.18877700 -1.48711600

C 3.49145400 3.06865300 -0.03229900

H 3.80328300 4.10677000 -0.15052400

H 4.13177000 2.43853800 -0.64896600

H 3.62421200 2.78251100 1.01119900

C -2.10392700 -0.08373500 0.27857000

C -4.41090900 0.18861000 0.56804900

H -4.25712300 1.21135300 0.91118300

H -4.40151700 -0.46865800 1.43737800

C -5.67382900 0.04116000 -0.24930500

H -6.53909400 0.30539200 0.35931400

H -5.78953900 -0.98665000 -0.59250400

H -5.64488500 0.69850100 -1.11795600

C 1.12165500 -1.94167100 -2.35269100

H 0.08062500 -2.26966200 -2.39240800

H 1.16867300 -0.91756000 -2.72628900

H 1.73219700 -2.59844400 -2.96683700

The coordinates (Å), SCF energies, enthalpies at 298K, and Gibbs free energies at 298K for the optimized structures **5a':**

M05-2X SCF energy: -990.75948588 a.u.

M05-2X enthalpy: -990.4318979 a.u.

M05-2X free energy: -990.5070189 a.u.

M05-2X SCF energy in solution: -991.04506587 a.u.

M05-2X enthalpy in solution: -990.7174779 a.u.

M05-2X free energy in solution: -990.7925989 a.u.

O 3.16830600 1.36293900 0.90497100

O 0.22347200 2.82399800 -1.08484200

O -0.19047100 -2.17645100 -0.96414100

O 1.70109300 -1.49064000 0.06638100

O -1.66950800 -0.50561500 1.25786300

O -3.24852800 -0.14405700 -0.33013900

N 0.21547100 0.05751900 -0.59769200

N -1.12207100 0.31770400 -0.80083700

H -1.42549100 0.36392600 -1.75947300

C 2.85893600 0.94752400 -1.43822500

H 3.88076100 1.30881900 -1.52466200

H 2.18226200 1.51941000 -2.07477700

H 2.80842500 -0.09695300 -1.74574700

C 2.42401800 1.07597200 -0.00338600

C 0.91438600 0.98220400 0.28389100

C 0.45127100 2.42713900 0.03310300

C 0.51340100 -1.30360700 -0.52058100

C 2.12993300 -2.86813100 0.16180700

H 2.17532600 -3.28536000 -0.84406300

H 1.37967500 -3.41846400 0.72823800

C 3.47777200 -2.85124300 0.84552000

H 3.84979800 -3.87039600 0.95351100

H 3.39642400 -2.39983300 1.83380200

H 4.19659800 -2.27620400 0.26160000

C -1.99937400 -0.16637500 0.14329000

C -4.25280700 -0.59242400 0.60542800

H -4.22172900 0.05268500 1.48350800

H -4.00449900 -1.60633800 0.91779500

C -5.58161800 -0.52175600 -0.11163100

H -6.37824000 -0.85289600 0.55521800

H -5.57387300 -1.16423500 -0.99172300

H -5.79228600 0.49987800 -0.42712200

H 0.78573800 0.71780500 1.33503700

C 0.40388700 3.28613600 1.26366900

H -0.40782800 2.93595100 1.90643200

H 1.33707000 3.16722100 1.81920800

H 0.24433500 4.32440500 0.98487900
